# What matters most to people living with epilepsy? A rapid review of qualitative research relating to health outcomes

**DOI:** 10.1111/epi.18570

**Published:** 2025-08-06

**Authors:** James W. Mitchell, John Doherty, Rachel Batchelor, Adam Noble, Anthony Marson

**Affiliations:** ^1^ Institute of Systems, Molecular, and Integrative Biology University of Liverpool The Clinical Sciences Centre, Aintree Hospital Site, Lower Lane Liverpool L9 7AL UK; ^2^ Department of Neurology Walton Centre NHS Foundation Trust Liverpool UK; ^3^ Oxford Institute of Clinical Psychology Training and Research University of Oxford Oxford UK; ^4^ Department of Public Health, Policy, and Systems, Institute of Population Health, Policy, and Systems University of Liverpool Liverpool UK

**Keywords:** adult epilepsy, focus groups, health outcomes, in‐depth interviews, qualitative review

## Abstract

At present, the outcomes measured and reported in clinical trials for adults with epilepsy are heterogenous and have often not been selected in consultation with people living with epilepsy. As part of a wider project developing a core outcome set for clinical trials for adults with epilepsy (the EPSET Project), a rapid review of the published qualitative literature was performed. MEDLINE was searched using an established qualitative methodological search filter to identify studies discussing the views of adults with epilepsy and their representatives. Eligible articles included published free‐text verbatim quotations from adults with epilepsy or their representatives discussing potential health outcomes. Data were coded and categorized in line with an established health outcome taxonomy. A total of 614 eligible articles were identified, of which 74 were included as they met the inclusion criteria. In total, the views of more than 2474 adults with epilepsy and 658 caregivers were included from six continents. Of the included studies, 77% used in‐depth interviews and 12% used focus groups. A total of 140 individual health outcomes were identified in the included studies across nine outcome domains: seizure‐related, cognitive–behavioral–neuropsychological, physical functioning–disability, emotional functioning, social role functioning, global quality of life, side effects and adverse events, fertility–pregnancy–offspring, and death. This is the first review of qualitative studies focusing on health outcomes for adults with epilepsy. The findings confirm that living with epilepsy represents more than living with seizures and highlight the impact of neuropsychiatric symptoms in these populations. Importantly, many outcomes identified from this review are not measured and reported in studies assessing the effectiveness and safety of treatments for epilepsy, supporting the development of a lived experience‐informed core outcome set for future effectiveness studies. Furthermore, the findings could be used to inform clinical practice and noninterventional studies across broad geographic, societal, and cultural settings.

**Key points**

- A synthesis of published qualitative literature was undertaken, representing the views of more than 3100 people with epilepsy and caregivers.
- Data about outcomes were extracted, with 140 outcomes identified across nine domains.
- Outcomes in the social and role functioning and "emotional functioning" domains were most frequently reported.
- The results should have wide‐reaching implications, providing new insights on what matters most to people with epilepsy.

## 1 INTRODUCTION

Randomized controlled trials are the gold standard source of evidence about the effectiveness and safety of treatments, informing guidelines and treatment decisions for people with epilepsy (PWE). Alongside continued drug development, there is a need for refining clinical trial design to ensure that the methods and results meet the needs of PWE taking part in trials, the wider epilepsy community, and regulatory agencies. There has, for instance, been much debate about reducing placebo exposure on ethical grounds, improving the external validity of regulatory studies, and ensuring trials are performed for some of the neglected rarer epilepsy syndromes.^1^, ^2^, ^3^, ^4^ Furthermore, and of particular pertinence to the current article, is discussion about the choice of "outcomes" or "endpoints" used in trials assessing the effectiveness and safety of interventions for PWE, particularly given the inconsistency of outcome reporting identified by Cochrane reviews and meta‐analyses.^5^

For other long‐term health conditions, there is an increasing international effort to identify core outcome sets (COSs), to obtain consensus on which outcomes should be reported as a minimum in research or routine clinical practice.^6^, ^7^, ^8^, ^9^ COSs for research facilitate interventional studies that are relevant to patients and health services and help standardize clinical trial methodology. They allow for research that is representative of international perspectives, and standardization of outcome measurement enables more meaningful results to be obtained from systematic reviews and meta‐analyses.^10^

Several consensus exercises have been conducted developing COSs for use in epilepsy populations. These include the COSs for routine practice clinic settings for infants, children, adolescents, and adults with epilepsy by the International Consortium for Health Outcomes Measurement,^11^, ^12^ for research assessing the effectiveness of interventions specifically for children with Rolandic epilepsy,^13^ and for quality‐of‐life outcomes for adults with drug‐resistant epilepsy in English‐speaking settings.^14^ At present, there is no internationally agreed COS for adult epilepsy interventional studies assessing treatment effectiveness. A COS for adult epilepsy effectiveness studies could help address some of the methodological difficulties in epilepsy clinical trial design by defining the minimum set of outcomes to be measured in relevant future research for adults with epilepsy.

The first step in COS development typically involves a review of existing knowledge to inform the consensus process. When developing a COS, it is important to include the patient experience, views of researchers and clinicians, and existing practice to inform the list of outcomes that are used to obtain consensus on which outcomes are essential to measure.^8^ Evidence from comparative studies assessing the use of qualitative methods in outcome set development indicates that including patient and caregiver perspectives leads to the identification of additional outcomes beyond those identified by health professionals alone, ensuring that the final COS is representative of a wider range of stakeholders.^15^, ^16^ Qualitative research exploring the lived experience perspective on what matters most about living with a health condition should therefore inform COS development, in particular by facilitating development of the "long list" of potential outcomes that develop in the information‐gathering phase of the process.

The epistemological and methodological theories underpinning qualitative research often highlight the importance of extensive interaction with research participants, giving weight to the meanings that people attach to their experiences and prioritizing depth of understanding over breadth of coverage.^17^ Therefore, qualitative research is well placed to improve understanding of patient perspectives on outcomes, as these studies allow people to express their views in an open‐ended manner representative of their lived experience, with the inductive approach to enquiry allowing for novel insights to be ascertained.

Primary qualitative research can be extremely resource‐intensive, particularly when considering training of a study team in qualitative research theories and methodology, undertaking patient interviews, transcribing the audio, and interpreting the data. Given that there is already a substantial body of qualitative literature published within the field of epilepsy, a systematic review of published qualitative studies was performed, rather than generating primary data by undertaking interviews or focus groups with people with lived experience. This alternative to conducting primary qualitative research, where suitable published studies are available, has been adopted by previous COS developers in the fields of diabetes mellitus, critical illness, bariatric and metabolic surgery, neonatal care, and tuberculosis.^18^, ^19^, ^20^, ^21^, ^22^ These reviews have all identified outcomes not reported in systematic reviews of clinical trials, further supporting the notion that qualitative evidence is needed to ensure that the outcome "long list" is comprehensive and does not omit outcomes deemed important to those with lived experience.

Here, we report the results of a rapid review, synthesizing the outcomes reported by adults with epilepsy when asked about their lived experience. This work was undertaken as part of a larger study developing a COS for effectiveness studies for adults with epilepsy, the EPSET project (www.epsetproject.org).^23^ Combining the results of this study with the results of a systematic review of outcomes reported in clinical trials will create a long list of outcomes^24^ to be used as the starting point for the consensus process to define the COS. The results should also have wider reaching implications, by providing new insights on what matters most to people living with epilepsy in a diverse range of geographic, societal, and cultural settings, which could additionally be used to influence clinical practice and noninterventional studies.

Please see online Supporting Information Appendix S1 for a schematic overview of the EPSET COS development process.

### 1.1 Review aims

This review aimed to:

- Identify outcomes important to adults with epilepsy from the published qualitative literature.
- Allow for the lived experience perspective to be incorporated into the initial stages of developing a COS for interventional studies for adults with epilepsy, by developing a "long list" using multistakeholder coproduction.

## 2 MATERIALS AND METHODS

### 2.1 Study identification

Rapid review methodology was used to synthesize evidence within a shortened time frame.^25^ This method uses an accelerated form of the traditional systematic review process and can provide an overview of evidence across a broad research area without providing exhaustive coverage of all published primary research.

We searched the single health‐related database, MEDLINE, with no date restrictions, on September 1, 2021. The search terms used are indicated in Table 1 and comprise qualitative methodological filters previously shown to identify qualitative research with high sensitivity and precision.^26^

**TABLE 1:** MEDLINE search strategy for qualitative review.

	((semi‐structured or semistructured or unstructured or informal or in‐depth or indepth or face‐to‐face or structured or guide) adj3 (interview* or discussion* or questionnaire*))	Title or abstract
	OR (focus group* or qualitative or ethnograph* or fieldwork or field work" or key informant)	MeSH terms
	OR interviews as topic/ or focus groups/ or narration/ or qualitative research/	
AND	Epilep*	Title or abstract
AND	symptom* OR treatment* OR living with OR experience*	Title or abstract
AND	patient* OR people with OR person with	Title or abstract

Abbreviation: MeSH, Medical Subject Headings.

Studies reporting qualitative primary evidence of the views and experiences of adults with epilepsy living with their condition and its treatment were eligible for inclusion. Studies focusing on infants, children, and adolescents with epilepsy and people with acute symptomatic seizures were outside of the scope of this review, as the intention was to inform the development of a COS for adults with epilepsy.

### 2.2 Study selection

J.W.M. and J.D. independently screened identified article abstracts, with any disagreements with study selection resolved through discussion with independent coresearcher Guleed Adan as an arbitrator. Full‐text articles were retrieved by J.W.M. and reviewed for studies meeting the following inclusion and exclusion criteria:

*Inclusion criteria*

- Person with epilepsy was age ≥18 years at time of data collection.
- Study focus was epilepsy and not the causative comorbidity (e.g., underling tumor or previous stroke).
- Qualitative methods were used as the primary source of data acquisition.

*Exclusion criteria*

- Review articles with no presentation of original verbatim quotes from people with epilepsy or their representatives.

### 2.3 Data extraction

For each included study, the following were extracted:

- Year of publication.
- Authors.
- Study title.
- Participant data: number in study, age, sex, epilepsy characteristics.
- Geographical location of study participants.
- Qualitative data collection and analysis methods used.
- Verbatim excerpts relevant to outcomes.
- Text, including participants' quotations, extracted verbatim from both the results and discussion section of the relevant papers. These data are available in Appendix S3 in the online Supporting Information.

### 2.4 Data categorization

A content analysis approach was used to synthesize data from the eligible studies. Outcomes were coded and analyzed using NVIVO software, and from the outcome codes produced we initially used the Core Outcome Measurement in Effectiveness Trials (COMET) taxonomy to categorize the verbatim text from the studies. Once granular terms were grouped within the COMET taxonomy; further categorization was performed iteratively, with development of new summary terms for outcomes that did not fit within the existing COMET taxonomy and to ensure an adequate level of granularity was maintained for seizure‐related outcomes. Where possible, terminology close to the language used within original verbatim text was used to describe the outcomes, and it was anticipated that these summary terms would be suitable to take forward to a multistakeholder consensus process to define a COS for treatment effectiveness studies for adults with epilepsy. Risk of bias and quality appraisals for individual studies were not performed, as it was anticipated that a broad range of qualitative research methodologies would be used in the included studies, and quality assessment in qualitative research remains controversial.^27^

### 2.5 Review registration

This review was registered on the PROSPERO database prior to commencement (CRD42020215156).^28^

## 3 RESULTS

### 3.1 Study characteristics

**FIGURE 1:**
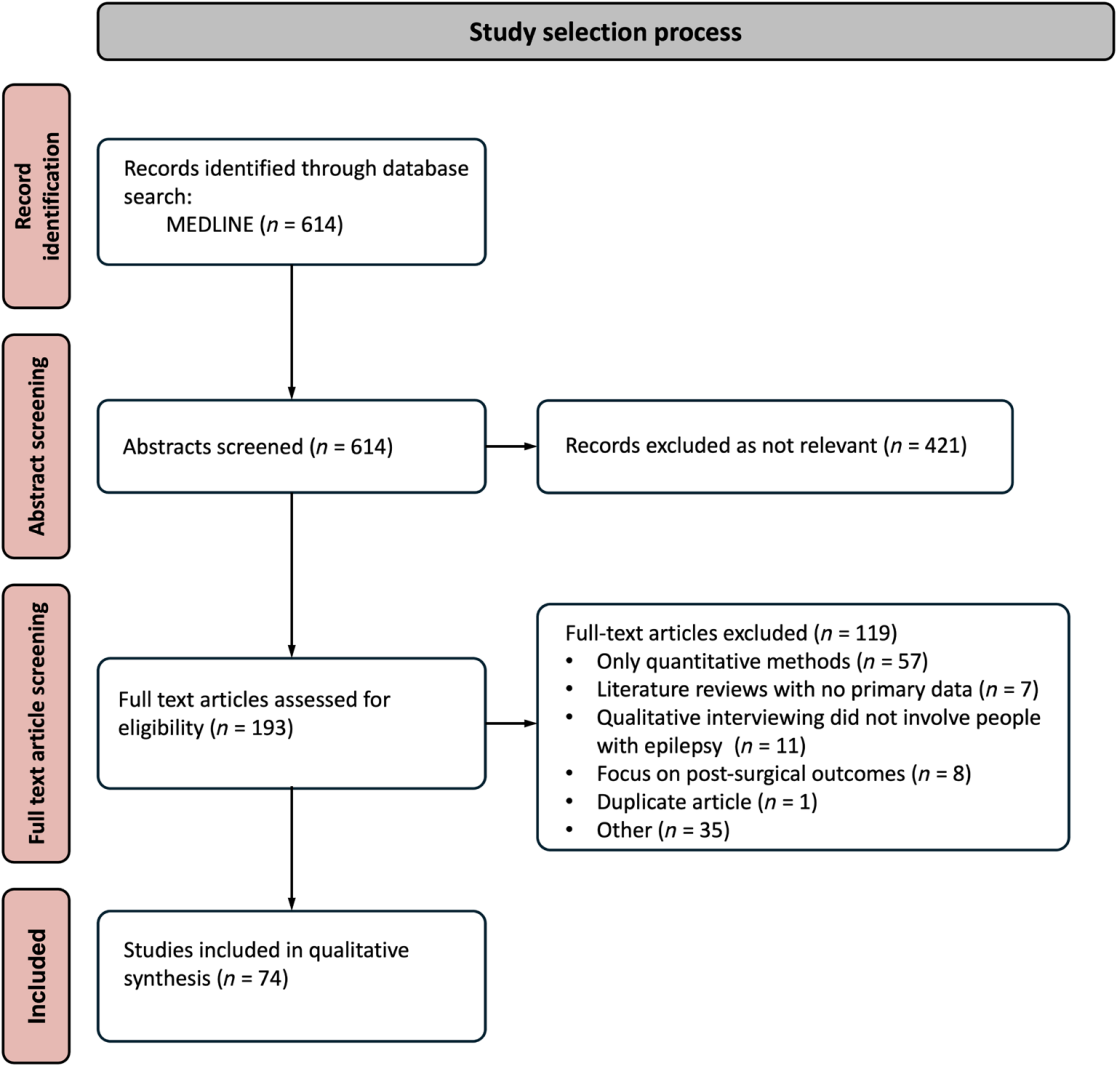
Study identification flow diagram for review of outcomes from qualitative literature.

The search returned 614 articles. Of these, 193 were retained after screening titles and abstracts. Following full‐text review, 119 studies were excluded, as they did not meet the inclusion criteria or presented duplicate data. The study selection process is outlined in (Figure 1).

**FIGURE 2:**
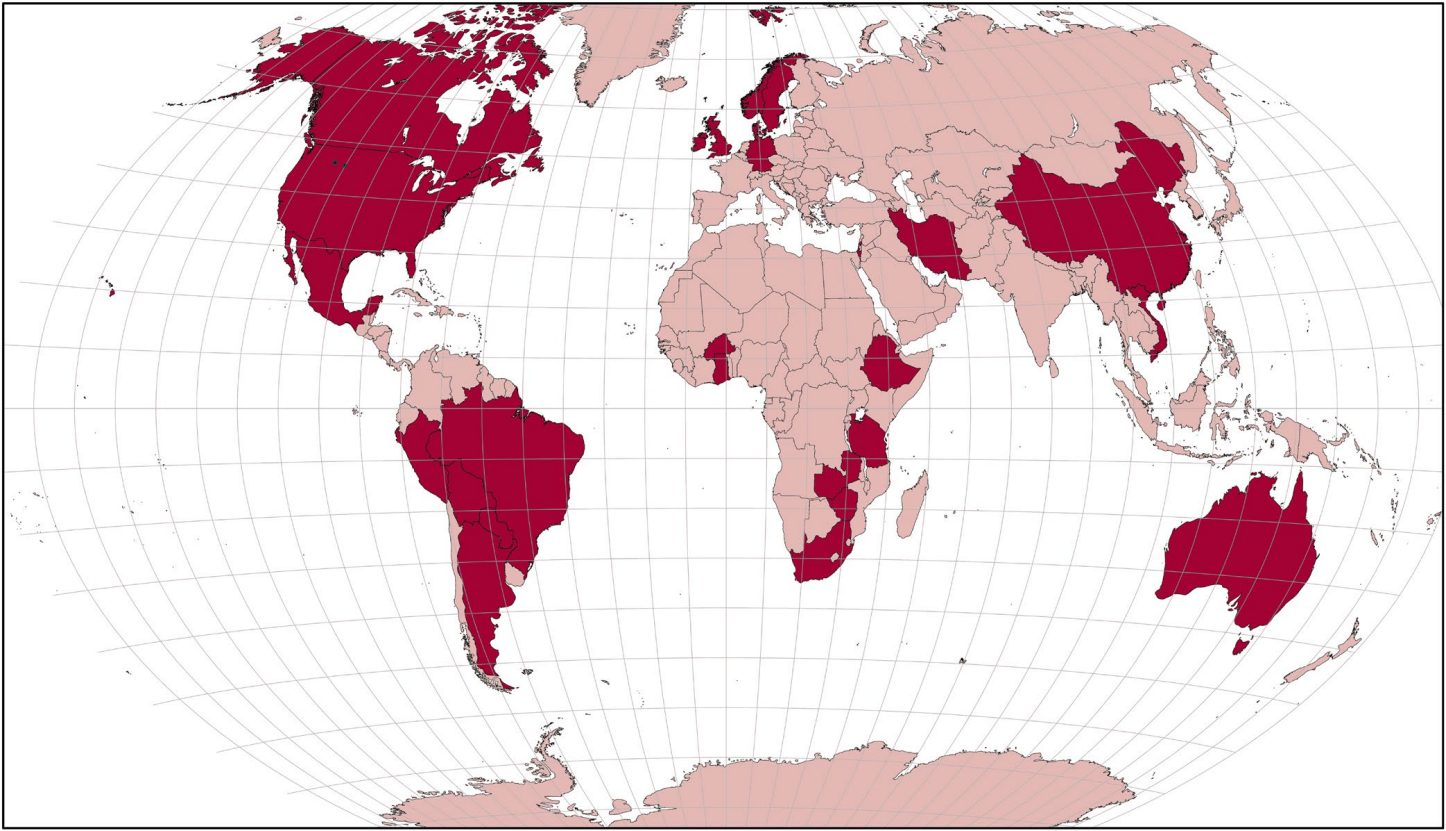
Geographical location of adults with epilepsy in included qualitative studies. Dark red shading represents country of residence of study participants from included studies. Map generated using mapchart.net.

In total, the views of more than 2474 adults with epilepsy and 658 caregivers or significant others were included (median = 20 adults with epilepsy, range = 4–632) from six continents. The geographical distribution of participants in the included studies is outlined in Figure 2.

All studies included PWE, with one study also involving another illness group (multiple sclerosis)^29^ and many including views of health care professionals and caregivers. A range of research enquiries were covered in the included studies, with many examining the impact of living with epilepsy broadly within a particular geographical, cultural, or population context.^30^, ^31^, ^32^, ^33^, ^34^, ^35^, ^36^, ^37^, ^38^, ^39^, ^40^, ^41^, ^42^, ^43^, ^44^, ^45^, ^46^ Others explored the lived experience perspective in more specific settings, such as the experience of visiting an emergency department due to seizures and their consequences,^47^ developing complex educational and behavioral interventions for assessment in randomized controlled trials,^48^, ^49^ as part of a nested study within a randomized controlled trial to provide an in‐depth exploration of the views about a treatment intervention,^50^ or in the item generation phase of health outcome measurement instrument development.^51^ Many studies examined specific aspects of living with epilepsy in society, such as stigma and discrimination,^51^, ^52^, ^53^, ^54^, ^55^ self‐management,^56^ and acceptance of the diagnosis.^57^

Most studies included people with a wide range of epilepsy classifications, using purposive sampling to ensure that views represented a broad range of PWE. Some studies specifically included people with newly diagnosed epilepsy^58^, ^59^, ^60^; others included only those with treatment‐refractory epilepsy.^31^, ^34^, ^49^, ^50^, ^61^ Some exclusively recruited people with epilepsy and intellectual or learning disability,^35^, ^62^ some only included people with focal onset epilepsy,^34^, ^54^, ^56^, ^63^ and others exclusively included people with generalized or genetic epilepsy syndromes.^64^, ^65^ Some studies also explored aspects of living with specific epilepsy syndromes such as tuberous sclerosis complex syndrome.^37^

It was not possible to provide an aggregated summary of participant demographic and clinical characteristics, given that these were not provided for some of the studies. In 14 of the included studies, no information regarding the classification, duration, or severity of epilepsy of its participants was provided in the article. Where demographic data were provided, it was often summarized as categorical rather than continuous variables, with different categories used in different studies, or only summary statistics reported.

A summary of included studies is provided in Appendix S2 in the online Supporting Information.

Of the included studies, 77% used in‐depth interviews as their primary data collection method, 12% used focus groups, 4% used focus groups and supplementary in‐depth interviews, and 7% used mixed methods approaches typically combining quantitative surveys with analysis of free‐text data from open‐ended questions. A range of epistemological and methodological approaches were described, including grounded theory, ethnography, phenomenology, narrative research, and mixed methods research. Where multiple stakeholders were involved in a study representing many conditions or disease processes, only data pertaining to PWE or their caregivers in their own voice were extracted, to ensure the views presented are as representative as possible of the views of the lived experience, without researcher or clinician interpretation.

Analysis of outcomes had been planned to continue across consecutive retrospective studies until five consecutive articles demonstrated outcome saturation, that is, no new outcomes identified. Outcome extraction saturation was never reached, meaning that data from all 74 included studies were used for the outcome synthesis.

### 3.2 Data categorization

A total of 140 individual outcomes were identified from the included studies and categorized according to the summary outcome framework that was informed by the COMET taxonomy, and the Wilson and Cleary conceptual model of health‐related quality of life, which integrates symptom status, functional state, general health perceptions, and the psychosocial aspects of health status.^66^, ^67^ The framework of domains and outcomes is outlined in Table 2. A table of all outcomes with supporting verbatim quotes from all of the included studies is available in Appendix S3 in the online Supporting Information.

**TABLE 2:** Domains and outcomes identified from verbatim quotations from participants in studies.

Domain	Outcome	Number of references
Seizure outcomes	Seizure freedom	15
	Seizure‐related injury	13
	Seizure frequency	10
	Experience of seizures (general)	10
	Experience of auras	5
	Focal onset seizures without generalization	4
	Seizure with impaired awareness, including generalized tonic–clonic seizures	4
	Postictal drowsiness	4
	Seizure duration	4
	Difficulty with breathing during seizure	3
	Loss of motor control during seizures	3
	Postictal symptoms—other	3
	Ability to identify seizure triggers	2
	Duration of postictal recovery	2
	Experience of myoclonic jerks/myoclonic seizure	2
	Seizure‐related loss of awareness	2
	Seizure severity	2
	Status epilepticus/prolonged seizures requiring rescue medication	2
	Unpredictability of seizures	2
	*Other outcomes only identified in one study* Brain injury following prolonged seizures Cognitive symptoms during seizures Emotional distress during/following seizure Experience of sensory phenomena during seizure Motor symptoms of seizure Only focal seizures (without generalization/impaired awareness) Seizure‐related amnesia	
Cognitive, behavioral, and mental health outcomes	Symptoms of depression	11
	Poor memory or concentration	11
	Anxiety—general	8
	Knowledge about epilepsy (satisfaction)	8
	Suicidality	5
	Anxiety symptoms from seizure‐related uncertainty	4
	Sense of hopelessness	4
	*Other outcomes only identified in one study* Safety consequences due to poor memory Word‐finding difficulty Impact of poor memory on medication compliance	
Sleep and related outcomes	Adequate sleep quality and duration	1
Physical functioning, participation, and disability	Independence (or loss of independence)	7
	Ability to go swimming	5
	Ability to exercise	4
	Sexuality and sexual functioning	4

	Ability to do gardening	2
	Ability to exercise alone	2
*Other outcomes only identified in one study* Ability to go shopping Ability to partake in team sports Ability to go do dance Ability to ride bicycle Ability to go hiking/mountaineering	
Emotional functioning	Perceived stigma or discrimination	32
	Ability to disclose diagnosis to others	13
	Acceptance of epilepsy	27
	Fear of seizures—seizure‐related worry	9
	Fear of seizure‐related injury	8
	Perception of being in control	8
	Sense of embarrassment	8
	Sense of normality	8
	Sense of isolation	7
	Perception of lack of control	6
	Self‐confidence	5
	Sense of being defined by epilepsy	5
	Self‐efficacy (regarding uncertainty of seizures)	4
	Self‐resilience or self‐efficacy (general)	4
	Fear of medication side effects	3
	Low self‐esteem	3
	Impact of epilepsy on sense of self	3
	Sense of not being defined by epilepsy	3
	Fear of SUDEP	2
	*Other outcomes only identified in one study* Positive sense of self Absence of seizure‐related worry Acceptance of SUDEP risk Fear of seizure while swimming Feeling of vulnerability Irritability Negative sense of self Anger or frustration with seizures Sense of being helpless	
Social and role functioning	Work status	25
	Driving	20
	Impact on relationships and friendships	17
	Impact on education and schooling	13
	Ability to socialize	12
	Ability to form companionship or get married	11
	Ability to fulfill parental role	9
	Ability to fulfill work role	9

	Psychosocial burden on others	9
	Sense of social exclusion or alienation	8
	Ability to leave the house	7
	Dependence on others	7
	Ability to have children	6
	Change in social behaviors due to seizures	5
	Burden from not driving	4
	Acceptance by others (no stigma)	4
	Exclusion by family	3
	Ability to develop a career/profession	3
	Sense of belonging (others have epilepsy too)	3
	Ability to go on holiday	2
	Impact on gender identify	2
	Ability to make new friends	2
	Burden on caregivers	2
	Burden on friends and family	2
*Other outcomes only identified in one study* Inability to drink alcohol socially Career progression Involvement with law enforcement agency due to seizure‐related behavior	
Life impact outcomes, including global quality of life	Financial impact of medication	7
	Financial security	6
	Financial impact of not working	5
	HRQOL not otherwise specified	3
	*Other outcome only identified in one study* Financial impact of hospital attendance	
Health resource use	Need for hospital or emergency department attendance	8
	Unnecessary hospital attendance	2
	*Other outcome only identified in one study* Need for emergency ambulance	
Side effect outcomes and drug monitoring	Medication side effect (general)	10
	Medication‐related somnolence	9
	Medication‐related weight gain	7
	Medication‐related mood changes	5
	Medication‐related cognitive disturbance	5
	Medication‐related fatigue	4
	Impact of medications on offspring health	4
	Medication‐related weight loss	3
	Medication‐related irritability	3
	*Other outcomes only identified in one study* Medication‐related aggression and impulsivity Medication‐related excess sweating Medication interactions Medication‐related anxiety Medication‐related osteopenia/osteoporosis Medication‐related dizziness Medication‐related alopecia Medication‐related appetite change Medication‐related headaches Medication‐related nausea Medication‐related personality change Paradoxical medication‐related seizures Medication‐related sleep disturbance Medication‐related vomiting	
Pregnancy and offspring outcomes	Ability to get pregnant	3
	Impact of seizure on fetal health and child development (including congenital malformations)	2
	Unintended pregnancy	2
	*Other outcome only identified in one study* Ability to breastfeed	
Death	Mortality	4
	SUDEP	2

Abbreviations: HRQOL, health‐related quality of life; SUDEP, sudden unexpected death in epilepsy.

### 3.3 Seizures

Seizure‐related outcomes were commonly discussed, with "seizure freedom" coded in 15 studies and "seizure‐related injury" as a consequence of seizures in 13 studies. Seizure frequency was the third most frequently coded theme in this domain and highlighted in 10 studies, and some participants specifically discussed how improved seizure frequency often led to improved engagement with work and social functioning:

I only have maybe one seizure a week now … so now I can get almost all my work done during the week and not have to catch up on weekends. [Adult with recently diagnosed epilepsy, interviewed about self‐management for epilepsy]^59^

Participants in four of the included studies discussed how postictal impairments (e.g., drowsiness) could be important given the potential impact on ability to function:

The generalised [seizure], the recovery time as well is excessive for me, I need to sleep for hours, so, if that happens at work or when you are out, it is a, it can just ruin a day you just lose a day basically, which is not good for me. [Woman with recently diagnosed epilepsy, and person of childbearing potential]^68^

Other seizure‐related outcomes included aspects connected to ictal components of seizures, such as experience of auras, whether seizures generalize and therefore lead to loss of awareness, and the duration of seizures, including status epilepticus.

### 3.4 Cognitive, behavioral, and mental health

Cognitive and neuropsychiatric outcomes were also frequently considered important by PWE, with "symptoms of depression" and "memory impairment" both coded in 11 studies. Much of the verbatim text relating to depression was broad, whereas in some instances specific perceived symptoms of depression were highlighted, including reduced self‐esteem, fatigue in the context of low mood, feelings of hopelessness, negative ruminations, and suicidality. Anxiety symptoms were discussed as specific seizure‐related worry in four studies, with participants discussing feeling “nervous about going out [due to seizures],” and discussed in a more general context in eight studies.

The concept of "memory impairment" was discussed by PWE as well as their caregivers, and often the verbatim text referred to the cognitive domains of working memory and attention:

I had a lot of problems with the cognitive impairments. It wasn't possible to study. I couldn't, you know, prepare meals. I couldn't follow a recipe. [Participant from focus group of people with epilepsy discussing what is important for their rehabilitation]^69^

In addition, four participants across two studies specifically discussed the impact of word‐finding difficulty, thought to be a consequence of epilepsy or its treatment.

### 3.5 Physical functioning, participation, and disability

The impact of epilepsy on physical functioning was coded in 18 studies and varied from very specific impairments such as the ability to go swimming unattended or ride a bicycle, to more generic comments about independence and the sense of burden felt by many with epilepsy. Of note, sexual dysfunction due to epilepsy and its treatment was coded from 11 participant quotes from four studies. This included comments about lack of interest and sexual desire, difficulty with arousal and orgasm, and fear of a seizure during sexual intercourse:

It's as important as the medicine, as important as knowing that I have to be careful with swimming or I should stop driving for six months. It's just as important to discuss how my sexuality is affected. Maybe it won't be affected, but it's a possibility and there are ways of treating it. [Man with epilepsy aged 49 years]^29^

You mentioned orgasm … this is something I only started allowing the past couple of years. Because I lose control … I think "what if I suddenly have a seizure?" [Woman with epilepsy aged 39 years]^29^

### 3.6 Emotional functioning

Frequent outcomes categorized within the emotional functioning domain were "perceived stigma and discrimination," which was coded 71 times across 32 studies, and the personal "acceptance of epilepsy," which was coded 38 times across 27 studies. Stigma was discussed by participants from varied geographical regions, and from broad socioeconomic backgrounds. A person with epilepsy from rural Burkina Faso, for example, mentioned “I am not respected. I do not have the right to go to public places. I have been given a separate cup and plate. They do not want me to get married…”^36^ and a young adult with focal epilepsy from Sweden discussed the difficulty with forming new relationships: “Say you meet some new people that you haven't met before and they find out you have epilepsy, well then they take three steps back, sort of.”^70^ "Acceptance of epilepsy" as outcome describes a sense of coming to terms with the diagnosis and often living with seizures and their consequences. Other outcomes coded in this domain included constructs such as a "perception of being in control," a "sense of embarrassment," and "self‐confidence."

### 3.7 Social and role functioning

The most commonly coded outcome within the social and role functioning domain was "work status," coded 43 times across 25 studies, highlighting the significant impact that epilepsy can have on employment. This was impacted by many factors, including transportation difficulties due to not being able to drive and fear of seizure‐related injuries for those working with machinery, as well as stigma that may lead to discrimination:

While my educational background is good, finding a job is difficult; I have tried many times without success, employers do not employ me because of my disease. As I may experience seizures on the job, I disclose my condition to potential employers, but being truthful has affected me negatively. Employers think I would infect other employees with the disease. Besides, they believe I would not perform up to expectation or that being an epileptic, I am incompetent. [Person with epilepsy from Accra, Ghana]^52^

Other outcomes coded within the social and role functioning domain were impact on relationships, friendships, and marriage and the impact of epilepsy on the ability to socialize. Specific reference to the impact of epilepsy and seizures on driving was coded 25 times across 20 studies and was frequently discussed by PWE in the context of their quality of life. Although restrictions for driving for PWE vary across regions, common implications included difficulty with traveling to a place of work, the inability to fulfill some employment roles due to license restrictions, struggling with fulfilling the parental role with providing transportation for children, and a sense of burden on others to help with transportation.

### 3.8 Other impact and global quality of life

Other impact and global quality of life outcomes pertain to generic comments about quality of life, well‐being, or the impact of living with epilepsy that could not be coded in any more granular detail in other sections of the taxonomy. Generic reference to quality of life was coded from three studies.^45^, ^71^, ^72^ This domain also includes outcomes related to the financial impact of living with epilepsy, including costs for treatments.

### 3.9 Side effects or adverse effects from treatments

Outcomes relating to side effects or adverse events were coded 84 times across 29 of the included studies. The most frequently identified adverse outcome relating to treatment was "medication‐related somnolence," which was coded across nine studies and frequently involved discussion of its impact on functioning, for example, engaging with education:

That medication makes me so sleepy … that's why I don't take it if I have to stay up and write a paper or have an early class. [Adult with newly diagnosed epilepsy from the USA]^59^

Other frequently coded outcomes within this domain included medication‐related weight gain in seven studies, medication‐related mood changes in five studies, and medication‐related cognitive disturbance also in five studies. "Impact of medications on offspring health" was coded across four studies, all discussed by women living with epilepsy:

[If] I was actually planning to have a kid, or try and get pregnant and if I did I wouldn't want the Epilim to do anything, because I know there's a high chance that the Epilim would have done something to my child if I had got pregnant. I thought if I do get pregnant at least I'm not going to be damaging my child in any way. [25‐year‐old woman with a 10‐year history of seizures from the UK, discussing medication switching]^73^

Whereas most participants discussed this consequence broadly, some discussed specific effects, including major congenital malformations as well as neurodevelopmental outcomes for their offspring.

### 3.10 Fertility and pregnancy, including offspring outcomes

The "ability to get pregnant and have a healthy pregnancy" was coded across three studies, two of which were specifically designed to ascertain the views of women living with epilepsy.^43^, ^74^ Women also discussed the impact of pregnancy on seizure control and expressed anxieties about the risks of antiseizure medication in pregnancy. One woman from the included studies discussed the difficult balance between obtaining seizure control and minimizing the potential teratogenicity of antiseizure medication:

[With the antiseizure medication] I wanted to reduce the risk of anything happening to the baby. But I didn't want to, you know, like double my chance of having a fit and losing the baby altogether, if you know what I mean, so I sort of had to balance baby over me. [Woman of childbearing potential with established epilepsy]^68^

Specific mention to antiseizure medication reducing the effectiveness of oral contraceptives and therefore increasing the risk of unintended pregnancy as an outcome was coded in two studies.^74^, ^75^ The ability to breastfeed was discussed in just one paper,^43^ and paternal fertility was not specifically identified from the included literature.

### 3.11 Death

Mortality as an outcome was coded across six studies and discussed specifically in the context of sudden unexpected death in epilepsy (SUDEP) in two studies. As one 21‐year‐old woman with epilepsy explained, “It's just quite scary to think that one night we, any one of us, might just go to sleep and never wake up.”^75^

## 4 DISCUSSION

This is the first review of qualitative studies focusing on outcomes for adults with epilepsy. Rapid review methodology and a synthesis of outcomes from the published qualitative literature has been undertaken, providing the foundation of work to develop a COS for treatment effectiveness studies for adults with epilepsy. In total, 74 articles were included, representing a variety of qualitative methodological and epistemological paradigms. The synthesized framework of 140 outcomes and nine domains represents the views of more than 3100 PWE and significant others.

A major strength is that the outcomes identified come from in‐depth interviews, focus groups, and free‐text data across a range of epilepsy classifications and severities, and diverse cultural, geographical, and socioeconomic contexts. The outcomes therefore should provide insights into the full spectrum of health‐related experiences, encountered by people living with epilepsy in a diverse range of cultural contexts.

Outcomes in the "social and role functioning" and "emotional functioning" domains were the most frequently coded across the included studies, with 275 and 271 quotations coded for each domain, respectively. This compares to 129 quotations coded for seizure‐related outcomes. The notion that living with epilepsy represents "more than just seizures" has been long established,^76^ particularly given that psychological constructs including anxiety and depression explain approximately one third of the variance in quantitative assessments of health‐related quality of life across multiple studies in different populations, compared to one fifth of the variance explained by seizure‐related factors.^77^ The high burden of neuropsychiatric "comorbidity" in epilepsy is also reflected in large population‐level studies. For instance, Danish registry data demonstrate that psychiatric comorbidity is 2–3 times higher in people living with epilepsy compared to the general population.^78^ That treatment‐related adverse effects were identified as important from the qualitative literature, as indicated by the large number of codes, aligns with the findings from multiple cross‐sectional studies that demonstrate correlations between the presence of side effects and health‐related quality of life scores in PWE.^79^, ^80^, ^81^, ^82^ These established relationships likely explain why behavioral, psychiatric, and adverse event outcomes and their impact on functional status and well‐being were frequently coded from the in‐depth interview and focus group data. Of note, the outcomes identified from this review of qualitative literature strongly align with the broad range of nonseizure outcomes identified in the recently published COS for children with epilepsy^13^ and adults with treatment‐refractory epilepsy in clinical settings.^14^

Importantly, many novel outcomes identified from this review of qualitative literature were not identified in the associated review of outcomes measured and reported in phase III and IV interventional studies for adults with epilepsy.^24^ These include measuring SUDEP as a specific cause of mortality, specific cognitive impairments including word‐finding difficulty, and many well‐defined functioning status outcomes including the ability to exercise and sexual function. Other novel outcomes included emotional functioning constructs, including acceptance of a diagnosis of epilepsy, a sense of normality, and a sense of isolation. This confirms the importance of including the lived experience perspective in the early stages of developing COSs; otherwise outcomes deemed important by those with lived experience risk being missed when developing the initial list of outcomes for inclusion in the consensus process. The need to include the lived experience perspective at this stage is highlighted by the COS‐STAD (Core Outcome Set‐Standards for Development) consensus on minimum methodological standards,^83^ and similar methods using qualitative reviews to identify outcomes have been successfully adopted by other COS developers.^18^, ^19^, ^20^, ^21^, ^22^

There are several limitations that need to be considered when interpreting the findings of this review. First, due to limited resources, rapid review methodology using only one bibliographic database, MEDLINE, was used, and the gray literature was not searched. Although this may mean that some relevant studies have been missed, the aim was not to provide an exhaustive review of all the published qualitative literature pertaining to the experience and perspectives of adults living with epilepsy, but rather to capture the likely range of outcomes considered important on the basis of lived experience for inclusion in the initial long list of outcomes that forms the basis for subsequent discussions about a COS for effectiveness studies for adults with epilepsy. This review identified outcomes from a greater number of studies compared to other COS developers,^18^, ^19^ including those who performed a systematic review and searched multiple bibliographic databases to identify relevant articles.^21^ This may reflect that epilepsy has a high prevalence and disease burden,^84^ and there has been a rich interest in researching the impact of living with epilepsy using qualitative methods historically.^85^

The evidence was largely concealed within the included studies, with studies only presenting pertinent participant verbatim quotes to justify theory or concepts, rather than providing full transcripts of data generated by in‐depth interviews or focus groups. For example, although some publications provided rich data from in‐depth interviews in Supporting Information,^56^ others did not and included only a few quotations within the main body of the published article.^86^ Not having all primary data available for analysis could mean that certain outcomes were missed by this review. It is a risk of the otherwise efficient method that was used and supports moves by some in the research community to make more available anonymized transcripts for reuse via repositories.^87^ However, the likelihood that this risk was realized in the present study is low, given that such a large number of studies from varied contexts were deemed eligible and therefore included for data extraction and outcome identification. Furthermore, analysis of the outcome list from a lived experience focus group used to develop a COS for routine clinical practice for infants, children, and adults with epilepsy demonstrates that all the granular outcome concepts identified in the focus group were also identified in this review.^11^, ^12^

Another limitation is that many studies were included where the primary aim was not to describe outcomes. The methodological rationale was that given the open‐ended nature of qualitative questioning and data generation, there was likely to be data relevant to outcomes important to adults with epilepsy whenever an experience of living with epilepsy was discussed. It is therefore possible that including studies relating to a specific experience (e.g., understanding patients' accounts of hospital consultations)^73^ or a particular construct (e.g., stigma and epilepsy)^51^, ^52^, ^54^ or intervention (e.g., epilepsy self‐management course as part of a randomized controlled trial)^50^ may have impacted on the outcomes identified. However, given that 74 studies were included for data extraction and that in many of the studies with a more focused scope, data about general perceptions about living with epilepsy were still discussed, it is unlikely that important outcomes have been missed or misrepresented by including these studies.

Finally, the epistemological context of this review should be noted. Verbatim text in the included studies was aggregated into an outcome framework, using a deductive approach based on the existing outcomes taxonomy. This was based on the assumption that mapping of concepts from published interview, focus group, and free‐text survey data was a valid representation of outcomes important to those with lived experience. This differed from many of the included studies, which used inductive approaches to generate new theory, for example, using ethnography and narrative research methods. The risk with this approach is that coding based on an existing outcomes taxonomy may have altered the meaning of the included participants' perspectives. However, the advantage of using an established taxonomy as a starting point for developing the outcome and domain structure was to allow for comparisons to be made with the associated systematic review of outcomes reported in clinical trials,^24^ and to facilitate development of the outcome long list to take forward to the consensus process to develop a COS.

## 5 CONCLUSIONS

Rapid review methodology has been used to identify outcomes important to adults with epilepsy across published qualitative studies assessing the impact of living with epilepsy. In addition to informing the long list of outcomes to enter the consensus process to develop the COS for adults with epilepsy, the findings should have wider reaching implications by providing new insights on what matters most to people living with epilepsy in a diverse range of geographic, societal, and cultural settings. These could additionally be used to inform clinical practice and noninterventional studies.

## AUTHOR CONTRIBUTIONS

**James W. Mitchell:** Conceptualization; data curation; data analysis; methodology; writing—original draft preparation; writing—review and editing. **John Doherty**: data analysis; writing‐review and editing. **Rachel Batchelor:** Data analysis; methodology; writing—review and editing. **Adam Noble:** Conceptualization; data analysis; methodology; writing—review and editing. **Anthony Marson:** Conceptualization; data analysis; methodology; writing—review and editing.

## CONFLICT OF INTEREST STATEMENT

J.W.M. has received fees for consultancy from the International Consortium for Health Outcomes Measurement to facilitate the development of their epilepsy standard sets for routine practice. R.B. has nothing to disclose related to the submitted work. Outside the submitted work, at time of writing, R.B. was a member of the NICE Epilepsies Guideline Committee and was a lived experience representative on the RCPCH Epilepsy Board. The other authors have nothing to disclose related to the submitted work. We confirm that we have read the Journal's position on issues involved in ethical publication and affirm that this report is consistent with those guidelines

## Supporting information


DATA S1.

## Data Availability

The data that support the findings of this study are available from the corresponding author upon reasonable request.
