# Supplementary material for: What matters most to people living with epilepsy? A rapid review of qualitative research relating to health outcomes
Source: Epilepsia. 2025 Aug 6;66(11):4122–38. doi: 10.1111/epi.18570 (PMC12661290; doi:10.1111/epi.18570)
Supplement: Supplementary file 1 — DATA S1. [file EPI-66-4122-s001.docx]

**ONLINE SUPPLEMENTARY MATERIAL - What matters most to people living with epilepsy? A rapid review of qualitative research relating to health outcomes**

**Appendix 1 – Schematic overview of the EPSET Project.**

**Appendix 2 Characteristics of included studies from qualitative review**

| **Study No** | **Title ^Reference^** | **Aim** | **Year** | **Study Location** | **PWE (n)** | **Care-givers (n)** | **PWE age in years** | **PWE gender** | **Epilepsy related characteristics** | **Data collection method** | **Analysis method** | **Unique Outcomes Identified** |
| --- | --- | --- | --- | --- | --- | --- | --- | --- | --- | --- | --- | --- |
| 8 | Trustful communication in the medical encounter: Perspectives of immigrated people with epilepsy.^1^ | Primary aim was to explore experience of communication in the medical encounter from the perspectives of foreign born PWE. However also looked more broadly at patients' experience of living with epilepsy. | 2019 | Sweden | 20 | 0 | median=32 (range 20-62) | M=7 | Epilepsy diagnosis with first seizure >1 year ago | In-depth interviews | Content analysis | 8 |
| 33 | Negotiating the boundaries of the medical model: Experiences of people with epilepsy.^2^ | Explore how PWE explore their healthcare in the context of living with epilepsy | 2020 | UK | 39 | 0 | unclear | M=14 | Heterogeneous group. Duration of epilepsy 1-49 years. 33% seizure free | In-depth interviews | Interpretive phenomenological analysis | 8 |
| 35 | Are patients ready for integrated person-centred care? A qualitative study of people with epilepsy in Ireland.^3^ | Capture the qualitative experiences of PWE in the context of the care that they receive | 2020 | Ireland | 27 | 0 | range 18-55, median not given | M=10 | Heterogeneous group. Duration of epilepsy 1-42 years. 15% seizure free | Focus groups and In-depth interviews | Thematic analysis | 7 |
| 53 | A qualitative study of the communication and information needs of people with learning disabilities and epilepsy with physicians, nurses and carers.^4^ | Primary aim to investigate communication and information needs of people with learning disability and epilepsy. However, includes expressed views relevant to outcomes | 2019 | UK | 15 | 13 | not specified but eligible participants between 16 and 50 | M=6 | Heterogeneous group with mild learning disability | In-depth interviews | Thematic analysis | 6 |
| 58 | 'I hate wasting the hospital's time': Experiences of emergency department admissions of Australian people with epilepsy.^5^ | Examine the reasons for and extent of hospital emergency department (ED) attendance by people with epilepsy. | 2019 | Australia | 120 | unclear | unclear | unclear | Heterogeneous group, 22% seizure free | Mixed methods approach, majority of data quantitative survey items. Only data from free text responses to open ended questions reported here | Content analysis | 11 |
| 62 | A life with seizures: Argentine patients' perspectives about the impact of drug-resistant epilepsy on their lives.^6^ | Understand the experience of living with epilepsy in a developing nation | 2018 | Argentina, Bolivia, Peru, Paraguay | 20 | 0 | mean=32.8 (range 22-52) - raw data available | M=12 | Drug resistant epilepsy, at point of admission for vEEG | In-depth interviews | Thematic analysis | 40 |
| 63 | Epilepsy, identity, and the experience of the body.^7^ | To explore what the experience of adult-onset epilepsy meant for a person's identity. | 2018 | UK | 39 | 0 | unclear | M=14 | Duration of epilepsy 1-49 years, 33% with well controlled epilepsy | In-depth interviews | Interpretive phenomenological analysis | 10 |
| 69 | Experiences and perspectives of stigmatization and discrimination against people with epilepsy in Accra, Ghana.^8^ | Explore experiences and perspectives of stigmatisation and discrimination against PWE | 2018 | Ghana | 14 | 2 | range 20-55 years | M=7 | Duration of epilepsy >20 years | in-depth interviews | Thematic analysis | 12 |
| 74 | A qualitative examination and theoretical model of anxiety in adults with epilepsy.^9^ | Determine what factors may explain why some people with epilepsy develop anxiety and others do not | 2018 | Australia | 26 | 0 | mean=41.1 (SD=13.5, range 21-60) | M=10 | Heterogeneous group. Duration of epilepsy 0.5-54 years. 35% requiring >1 antiseizure medication | In-depth interviews | Grounded theory | 40 |
| 79 | Implementing integrated services for people with epilepsy in primary care in Ethiopia: a qualitative study.^10^ | Explore the perspectives of service users and caregivers on the accessibility, experience and perceived impact of epilepsy treatment | 2018 | Ethiopia | 13 | 3 | unclear | M=11 | Convulsive epilepsy, otherwise not specified. | In-depth interviews | Framework analysis | 21 |
| 84 | Experiences and informational needs on sexual health in people with epilepsy or multiple sclerosis: A focus group investigation.^11^ | Explore experiences and information needs regarding sexual health in PWE and MS | 2018 | Denmark | 15 | 0 | median=47 (range 30-68) | M=7 | Median duration of epilepsy 27 years. Otherwise not specified | Focus groups | Framework analysis | 13 |
| 103 | A longitudinal cohort study on the impact of clobazam shortage on patients with epilepsy.^12^ | Study the impact of clobazam shortage on PWE (mixed methods) | 2017 | Canada | 56 | 15 | unclear | unclear | Median duration of epilepsy 21 years, 34% seizure free, median number of anti-seizure medications of 2, 58% treatment refractory epilepsy | Mixed methods approach, majority of data quantitative survey items. Only data from free text responses to open ended questions reported here | Phenomenological approach | 8 |
| 115 | How can we enhance the sense of self-efficacy in epilepsy individual responses from 2 qualitative case reports.^13^ | Perception of mindfulness-oriented intervention for PWE and impact on well-being. | 2017 | Germany | 9 | 0 | unclear | unclear | Focal onset epilepsy | In-depth interviews | Unclear | 38 |
| 120 | Individual resilience as a strategy to counter employment barriers for people with epilepsy in Zimbabwe.^14^ | Explore employment experiences of PWE | 2017 | Zimbabwe | 8 | 0 | unclear (range 26-48) | M=4 | 4 childhood onset, 4 adult onset | In-depth interviews | Unclear | 12 |
| 136 | Becoming comfortable with "my" epilepsy: Strategies that patients use in the journey from diagnosis to acceptance and disclosure.^15^ | Explore how PWE reach a stage of being comfortable with epilepsy | 2017 | Ireland | 49 | 0 | unclear (categorical variables reported) | M=25 | Duration of epilepsy from <5 to >20 years, 33% seizure free | in-depth interviews | Grounded theory | 28 |
| 137 | The experience of epilepsy in later life: A qualitative exploration of illness representations.^16^ | Explore how older PWE appraise their condition through their lived experience. | 2017 | UK | 10 | 0 | mean=72 (SD=4.2) | M=3 | Heterogeneous group. | In-depth interviews | Interpretive phenomenological analysis | 28 |
| 139 | Mind the gap: exploring information gaps for the development of an online resource hub for epilepsy and depression.^17^ | Identify information needs of PWE and depression, particularly relating to resource access and resource gaps. | 2017 | UK | 10 | 0 | unclear | M=3 | Not specified, but all had comorbid depression. | In-depth interviews | Content analysis | 7 |
| 140 | The individual with epilepsy: perceptions about the disease and implications on quality of life.^18^ | Evaluate perceptions of PWE about epilepsy and impact on QoL | 2017 | Brazil | 30 | 0 | mean=45 (range 18-59) | M=13 | Refractory temporal lobe epilepsy | Mixed methods approach. Only data from in-depth interviews reported here | Content analysis | 11 |
| 144 | Developing and assessing the acceptability of an epilepsy fist aid training intervention for patients who visit UK emergency departments: a multi-method study of patients and professionals.^19^ | Overall study to develop complex intervention (epilepsy first aid training for patients visiting the Emergency Department). Here we report only findings from focus groups of PWE about their needs. | 2017 | UK | 13 | 10 | unclear | M=7 | unclear | Focus groups | Unclear | 10 |
| 146 | People with epilepsy obtain added value from education in groups: results of a qualitative study.^20^ | Perception of PWE about complex educational intervention | 2017 | UK | 20 | 0 | mean=44 | M=10 | Poorly controlled epilepsy (>2 seizures per year on medication) | In-depth interviews | Framework analysis | 8 |
| 151 | 'Epileptic', 'epileptic person' or 'person with epilepsy'? Bringing quantitative and qualitative evidence on the views of UK patients and carers to the terminology debate.^21^ | Assess preferences of terminology when referring to PWE. Some responses more generally map to patient outcomes. | 2017 | UK | 638 | 331 | median=39 (IQR 28-49) | M=149, | Heterogeneous group | Mixed methods approach, majority of data quantitative survey items. Only data from free text responses to open ended questions reported here | Thematic analysis | 6 |
| 156 | SUDEP: What do adult patients want to know?^22^ | Assess views about how and when to discuss SUDEP with PWE. Some responses more generally map to patient outcomes. | 2016 | Canada | 23 | 0 | median 33 | M=7 | Unclear | in-depth interviews and focus group | Content analysis | 7 |
| 158 | Sometimes, it just stops me from doing anything": A qualitative exploration of epilepsy management in people with intellectual disabilities and their carers.^23^ | Investigate experiences of living with epilepsy in PWE and intellectual disabilities | 2016 | UK | 14 | 14 | PWE mean=43 (SD 15.3) | M=6 | Heterogeneous group with intellectual disability | In-depth interviews | Thematic analysis | 28 |
| 169 | The psychosocial impact of exercising with epilepsy: A narrative analysis.^24^ | The psychosocial impact of exercising with epilepsy: A narrative analysis. | 2016 | UK | 4 | 0 | range 23-38 | M=1 | Heterogeneous group | In-depth interviews | Narrative analysis | 19 |
| 185 | "The others look at you as if you are a grave": a qualitative study of subjective experiences of patients with epilepsy regarding their treatment and care in Cape Town, South Africa.^25^ | Explore experience of living with epilepsy. | 2016 | South Africa | 12 | 0 | unclear | M=8 | Unclear | In-depth interviews | Thematic analysis | 23 |
| 188 | Does the concept of resilience contribute to understanding good quality of life in the context of epilepsy?^26^ | Understand how resilience impacts on quality of life for PWE | 2016 | UK | 67 | 0 | mean=46 (range 24-65) | M=32 | Heterogeneous group | In-depth interviews | Thematic analysis | 24 |
| 200 | Treatment and challenges with antiepileptic drugs in patients with juvenile myoclonic epilepsy.^27^ | Treatment and challenges with antiepileptic drugs in patients with juvenile myoclonic epilepsy. | 2019 | Norway | 10 | 0 | unclear | unclear | Diagnosis of JME | In-depth interviews | Unclear | 11 |
| 204 | Development and psychometric evaluation of the Perceived Social Stigma Questionnaire (PSSQ-for adults with epilepsy): A mixed method study.^28^ | Main aim to develop a patient questionnaire assessing percieved social stigma. Only data from qualitative interviews relevant to outcomes reported here. | 2019 | Iran | 20 | 0 | unclear | unclear | Heterogeneous group, at least 1 year since diagnosis, without co-morbid diagnoses | In-depth interviews | Content analysis | 12 |
| 205 | The experiences of Iranian patients with epilepsy from their disease: A content analysis.^29^ | Investigate experiences of patients with epilepsy in iran regarding stigma. | 2019 | Iran | 22 | 0 | mean=27 (SD 6.6) | M=12 | People with tonic-clonic seizures, and without significant intellectual disabilities | In-depth interviews | Content analysis | 9 |
| 207 | What really matters? A mixed methods study of treatment preferences and priorities among people with epilepsy in the UK.^30^ | Mixed methods approach to examine the views and experiences of PWE about a range of treatment options for epilepsy. | 2019 | UK | 56 | 0 | mean=40 (range 20-76) | M=29 | Heterogeneous group, 41% newly diagnosed | In-depth interviews | Thematic analysis | 32 |
| 208 | Knowledge, attitudes, and practices related to epilepsy in rural Burkina Faso.^31^ | Assess knowledge, beliefs, attitudes and practices regarding epilepsy and neurocysticercosis in the rural areas of Burkina Faso. | 2019 | Burkina Faso | 6 | 0 | unclear | M=21 | Unclear | In-depth interviews | Content analysis | 12 |
| 216 | 'We've made the best of it. But we do not have a normal life': families' experiences of tuberous sclerosis complex and seizure management.^32^ | Explore families' positive and negative experiences and attitudes towards TSC, epilepsy and medical management of seizures. Only data relevant to adults with TSC extracted here. | 2019 | UK | 7 | 6 | unclear | unclear | Patients with epilepsy and Tuberous Sclerosis, all received treatment with mTOR inhibitor | In-depth interviews | Framework analysis | 25 |
| 221 | Views of People With Epilepsy About Web-Based Self-Presentation: A Qualitative Study.^33^ | Whilst main focus aimed to examine web-based media use and self-presentation in a group of PWE, some data relevant to outcomes. | 2018 | UK | 14 | 0 | median=50 (range 33-73) | M=7 | Heterogeneous group, median 25 years since diagnosis (range 13-63), 50% experienced tonic-clonic seizures | In-depth interviews | Thematic analysis | 10 |
| 230 | PRO-based follow-up as a means of self-management support - an interpretive description of the patient perspective.^34^ | Main aim to explore how PWE experience the use of PRO-based follow up. Only data relevant to outcomes reported here. | 2018 | Denmark | 29 | 0 | unclear | M=14 | unclear | In-depth interviews | Interpretive description | 9 |
| 238 | Determining the disease management process for epileptic patients: A qualitative study.^35^ | Determine the disease management process in patients with epilepsy in Iran. | 2016 | Iran | 32 | 4 | range 18-61 | unclear | unclear | In-depth interviews | Grounded theory | 27 |
| 253 | Care for chronic illness in Australian general practice - focus groups of chronic disease self-help groups over 10 years: implications for chronic care systems reforms.^36^ | Examine experiences of chronic illness before and after introduction of incentives for longer consultations. Only data from PWE relevant to outcomes reported here | 2009 | Australia | 10 | 0 | unclear | M=5 | unclear | Focus groups | Grounded theory | 14 |
| 262 | Self-management for people with poorly controlled epilepsy: Participants' views of the UK Self-Management in epILEpsy (SMILE) program.^37^ | Examine the views and experiences of participants in self-management programme. Only data relevant to outcomes presented here. | 2015 | UK | 10 | 0 | range 21-60 | M=4 | Poorly controlled epilepsy (on AED and >1 seizure in past 12 months) | in-depth interviews and focus group | Thematic analysis | 10 |
| 276 | Epilepsy services in Ireland: 'A survey of people with epilepsy in relation to satisfaction, preferences and information provision'.^38^ | Mixed methods study reporting views of people with epilepsy with regards to health services delivery. | 2014 | Ireland | 102 | 0 | mean=37 (SD 12.9) | M=32 | unclear | Mixed methods approach, majority of data quantitative survey items. Only data from free text responses to open ended questions reported here | Thematic analysis | 15 |
| 280 | "It's good to know": experiences of gene identification and result disclosure in familial epilepsies.^39^ | Explore experience of receiving a genetic result in people with familial epilepsy. Only data relevant to outcomes in PWE presented here. | 2015 | Australia | 13 | 7 | unclear | unclear | Genetic epilepsies | In-depth interviews | Thematic analysis | 12 |
| 285 | A qualitative study of the reactions of young adults with epilepsy to SUDEP disclosure, perceptions of risks, views on the timing of disclosure, and behavioural change.^40^ | Examine young adult's responses to information about SUDEP. | 2014 | UK | 27 | 0 | range 18-29 | M=12 | Heterogeneous group, 30% seizure free over prior year | In-depth interviews | Thematic analysis | 5 |
| 286 | Perceptions and experiences of epilepsy among patients from black ethnic groups in South London.^41^ | Explore perceptions and experiences of epilepsy among black African and Caribbean people in South London | 2014 | UK | 11 | 0 | range 22-79 | M=5 | Heterogeneous group of people with established epilepsy (>1 year) | In-depth interviews | Thematic analysis | 21 |
| 288 | 'If you're gonna die, you're gonna die': Young adults' perceptions of sudden unexpected death in epilepsy.^42,43^ | To explore the views and experiences of young adults with epilepsy on the risks associated with, and information giving in relation to sudden unexpected death in epilepsy (SUDEP). | 2014 | UK | 27 | 0 | mean=22 (range 18-29) | M=11 | Heterogeneous group, epilepsy duration 1-27 years, 30% seizure free over prior year | In-depth interviews | Thematic analysis | 5 |
| 294 | Perspectives of adults with epilepsy and their support persons on self-management support. | To examine the type of support provided to PWE and its influence on self-management. | 2014 | USA | 22 | 16 | range 21-59 | M=7 | Heterogeneous group | In-depth interviews | Grounded theory | 14 |
| 310 | Exploring loss and replacement of loss for understanding the impacts of epilepsy onset: a qualitative investigation.^44^ | Explore the utility of the concept of loss and loss replacement as a means of gaining a fuller understanding of the implications of epilepsy diagnosis and overall QOL. | 2014 | UK | 67 (same as study 188) | 0 | mean=46 (range 24-65) | M=32 | Heterogeneous group | In-depth interviews | Thematic analysis | 34 |
| 317 | Stigma experience of people with epilepsy in Mexico and views of health care providers.^45^ | Explore the experience of epilepsy stigma. Mixed methods study, only data from qualitative interviewing presented here. | 2014 | Mexico | 10 | 10 | range 26-50 | M=5 | People with temporal lobe epilepsy, onset between 4 and 18 years of age | In-depth interviews | Unclear | 12 |
| 320 | Problems, needs, and useful strategies in older adults self-managing epilepsy: implications for patient education and future intervention programs.^46^ | Determine perceived self-management problems and needs encountered since diagnosis, as well as strategies used to address problems and needs. | 2014 | USA | 20 | 0 | mean=70 (range 60-80) | M=8 | Heterogeneous group, epilepsy onset after 60 years of age | In-depth interviews | Content analysis | 26 |
| 328 | The importance of the experiences of initial diagnosis and treatment failure when switching antiepileptic drugs.^47^ | Understand the issues that lead from the need to change AED and how this may influence someone's feelings regarding swapping to another drug. | 2013 | UK | 14 | 0 | mean=40 (range 17-68) | M=6 | Those within 4 months after changing anti-seizure medication, 71% with focal epilepsy | In-depth interviews | Thematic analysis | 23 |
| 332 | Patients' perception of epilepsy and threat to self-identity: a qualitative approach.^48^ | Explore perception of epilepsy of PWE in Iran, with focus on psychosocial impacts of diagnosis. | 2013 | Iran | 21 | 5 | unclear | unclear | unclear | In-depth interviews | Content analysis | 29 |
| 339 | What is important in rehabilitation for persons with epilepsy? Experiences from focus group interviews with patients and staff.^49^ | Identify issues experienced as essential in rehabilitation for PWE. | 2013 | Sweden | 17 | 0 | mean=40 (range 25-69) | M=3 | Heterogeneous group, 3-46 years since epilepsy diagnosis, 88% with focal epilepsy, 18% seizure free, 71% on anti-seizure medication polytherapy | Focus groups | Content analysis | 18 |
| 351 | A nurse-led self-management intervention for people who attend emergency departments with epilepsy: the patients' view.^50^ | Nested study, reporting the perceived support needs of patients who have attended a emergency department for epilepsy and benefits of nurse-led self-management intervention. Only data relevant to outcomes presented here. | 2012 | UK | 16 | 0 | median 38 (range 21-91) | M=7 | Heterogeneous group, 1->10 years since diagnosis, 50% with focal epilepsy, 50% with generalised epilepsy | In-depth interviews | Thematic analysis | 18 |
| 358 | Explanations given by people with epilepsy for using emergency medical services: a qualitative study.^51^ | Describes why PWE use emergency medical services in the UK. | 2012 | UK | 19 | 0 | median=39 (range 21-91) | M=9 | Heterogeneous group, 3-56 years since diagnosis, 10 with focal epilepsy | In-depth interviews | Thematic analysis | 13 |
| 369 | "The brain is such a delicate thing": an exploration of fear and seizures among young people with epilepsy.^52^ | Explore the emotional experience of young people with epilepsy. | 2012 | UK | 37 | 0 | range 16-28 | M=14 | Heterogeneous group, 1-25 years since diagnosis | In-depth interviews | Unclear | 23 |
| 384 | Living with epilepsy accompanied by cognitive difficulties: young adults' experiences.^53^ | Explore the experience of living with epilepsy and subjectively impaired cognition | 2011 | Sweden | 14 | 0 | range 18-35 | M=7 | Heterogeneous group, including childhood onset epilepsy | Focus groups | Content analysis | 47 |
| 397 | Patients' perceptions of living with epilepsy: a phenomenographic study.^54^ | Describe how PWE perceive living with epilepsy | 2010 | Sweden | 19 | 0 | unclear | M=7 | Heterogeneous group, duration of epilepsy 1-49 years, 26% with tonic-clonic seizures, 47% taking two or more anti-seizure medications | In-depth interviews | Phenomenographic method | 24 |
| 402 | Social-cultural aspects of epilepsy in Kilimanjaro Region, Tanzania: knowledge and experience among patients and carers.^55^ | Explore the experience of living with epilepsy in a broad social context. | 2010 | Tanzania | 32 | 19 | unclear | M=26 | 85% taking medication, otherwise unclear | In-depth interviews | Content analysis | 16 |
| 403 | The health care journeys experienced by people with epilepsy in Ireland: what are the implications for future service reform and development?.^56^ | Understand health care journey from symptom onset to first interaction with epilepsy service | 2010 | Ireland | 19 | unclear | unclear | M=9 | Newly diagnosed epilepsy, 63% on monotherapy | In-depth interviews | Interpretive Phenomenological Analysis | 19 |
| 429 | Multiple impacts of epilepsy and contributing factors: findings from an ethnographic study in Vietnam.^57^ | Understand the impact of epilepsy and attitudes towards PWE in Vietnam | 2009 | Vietnam | 32 | 27 | range 14-82 | M=14 | Heterogeneous group | In-depth interviews | Unclear | 31 |
| 439 | Understanding routine antiepileptic drug decisions: a qualitative analysis of patients' accounts of hospital consultations.^58^ | Describe the patient experience of decision making in the clinical encounter. Only data relevant to outcomes presented here | 2008 | UK | 47 | 0 | median 38 (range 15-68) | M=18 | Heterogeneous group, 9-35 years since diagnosis, 21% seizure free at time of interview | In-depth interviews | Grounded theory | 9 |
| 440 | Knowledge gaps and uncertainties about epilepsy: findings from an ethnographic study in China.^59^ | Main focus to identify knowledge gaps and uncertainty about living with epilepsy for PWE and their family in China | 2009 | China | 48 | 48 | range 15-69 | M=24 | unclear | In-depth interviews | Ethnography | 20 |
| 441 | Hybrid concept analysis of self-management in adults newly diagnosed with epilepsy.^60^ | Primary aim to define the concept of self-management for adults recently diagnosed with epilepsy. Only data relevant to outcomes presented here | 2008 | USA | 4 | 0 | mean=29 (range 20-28) | M=2 | Newly diagnosed (within 12 months) | In-depth interviews | Content analysis | 30 |
| 449 | Women's experiences living with epilepsy in Zambia.^61^ | Describe women's experiences of living with epilepsy in Zambia, with focus on experience of stigma, discrimination or deprivation. | 2008 | Zambia | >48 | 0 | unclear | M=0 | unclear | Focus groups | Content analysis | 26 |
| 451 | The use of biomedicine, complementary and alternative medicine, and ethnomedicine for the treatment of epilepsy among people of South Asian origin in the UK.^62^ | Main focus to study the use of biomedicine, complementary and alternative medicine and ethnomedicine in a sample PWE of South Asian origin | 2008 | UK | 30 | 16 | range 18-68 | M=15 | unclear | In-depth interviews | Framework analysis | 8 |
| 453 | The experience of discontinuing antiepileptic drug treatment: an exploratory investigation.^63^ | Explore patients' experience of discontinuing medication following a seizure-free period of 2 years | 2007 | UK | 12 | 1 | range 15-56 | M=5 | Patients with epilepsy and seizure freedom for 2 years, planning drug withdrawal | In-depth interviews | Phenomenological approach | 30 |
| 455 | 'What really annoys me is people take it like it's a disability', epilepsy, disability and identity among people of Pakistani origin living in the UK.^64^ | Explore attitudes towards PWE and impact on their lives from the perspective of people living in the UK of Pakistani Muslim heritage | 2008 | UK | 20 | 10 | unclear | unclear | People with epilepsy, without learning disability | In-depth interviews | Framework analysis or similar | 16 |
| 456 | Meanings of epilepsy in its sociocultural context and implications for stigma: findings from ethnographic studies in local communities in China and Vietnam.^65^ | Investigate beliefs about causes, course and treatment of epilepsy and QOL | 2008 | China and Vietnam | 81 | 74 | range 14-82 | M=39 | unclear | in-depth interviews and focus group | Thematic analysis | 15 |
| 460 | Living with epilepsy: ordinary people coping with extraordinary situations.^66^ | Study the experience of growing up with a chronic condition, with epilepsy being used as an example | 2007 | Israel | 14 | 0 | range 15-24 | M=3 | Heterogeneous group | In-depth interviews | Grounded theory | 20 |
| 494 | Stigma and safe havens: a medical sociological perspective on African-American female epilepsy patients.^67^ | Investigate knowledge, attitudes and beliefs associated with epilepsy among African American patients. | 2005 | USA | 10 | 0 | range 29-58 | M=0 | Age of onset 26-49 years, uncontrolled epilepsy in 60% | In-depth interviews | Unclear | 12 |
| 501 | Many people with epilepsy want to know more: a qualitative study.^68^ | Explore the information needs of PWE | 2005 | UK | 35 | 3 | unclear | M=15 | Heterogeneous group | In-depth interviews | Thematic analysis | 21 |
| 502 | Religious beliefs about causes and treatment of epilepsy.^69^ | Explore the influences of spiritual and religious beliefs on explanation of the cause of epilepsy, and the choice of treatment in people of South Asian origin | 2005 | UK | 30 | 16 | unclear | M=15 | unclear | In-depth interviews | Framework analysis | 8 |
| 528 | A 'real puzzle': the views of patients with epilepsy about the organisation of care.^70^ | Evaluate the views of PWE regarding their experience of healthcare services and healthcare needs | 2003 | UK | 19 | 0 | mean=44 (SD 13.3) | M=12 | People with epilepsy without learning disability | Focus groups | Unclear | 15 |
| 531 | Patient attitudes about treatments for intractable epilepsy.^71^ | Understand patient attitudes about the treatment of medically intractable epilepsy | 2003 | USA | 20 | 4 | unclear | M=12 | >1 seizure/month on one or more antiseizure medication, and decline in function over 6 months | Focus groups | Content analysis | 17 |
| 532 | How can a nurse intervention help people with newly diagnosed epilepsy? A qualitative study of patients' views.^72^ | Describe patients' views of the challenges posed by a new diagnosis of epilepsy and their assessment of a nurse intervention | 2001 | UK | 22 | 0 | unclear | unclear | Newly diagnosed epilepsy | In-depth interviews | Unclear | 13 |
| 567 | Quality of epilepsy treatment and services: the views of women with epilepsy.^73^ | Describe experiences of and satisfaction with care and treatment in both primary and secondary care | 1998 | UK | 18 | 0 | mean=37 (range 24-66) | M=0 | Heterogeneous group, 22% post-surgical, 22% seizure free at time of focus group, all on anti-seizure medication, 27% on monotherapy | Focus groups | Content analysis | 18 |
| 586 | Living with epilepsy: a qualitative study investigating the experiences of young people attending outpatient clinics in Leicester.^74^ | Investigate the experiences of young PWE attending outpatient clinics | 1996 | UK | 24 | 4 | range 13-25 | M=9 | Heterogeneous group, including people with learning disability | In-depth interviews | Thematic analysis | 9 |

**Appendix 3 Coded verbatim data relating to treatment outcomes from qualitative review**

A - SEIZURE OUTCOMES

| OUTCOME CODE | VERBATIM PARTICIPANT TEXT |
| --- | --- |
| Seizure freedom | **Article 062** “I was seizure free for one year, so I stopped taking my medication.”  **Article 069** “The seizures stopped, but I had a relapse a few months after I had started learning a trade. I was sent to another pastor for prayers, but the seizures did not stop.”  **Article 079** “They will give me the drug and, as I told you, they told me the disease will vanish one day in the future.”  **Article 115 supplement** “That I will be seizure free eventually.”  “Remained seizure free ‘for almost 20 years without medication, without anything.’”  **Article 151** “I haven’t had a seizure for several years, so I do not like to class myself as being ‘epileptic’ because it is controlled.”  **Article 200** “I have been seizure free for some months with lamotrigine and topiramate.”  **Article 207** “Once I am free [of seizures] for a few weeks, a couple of months, then probably [I can] start getting back into the old way of life and just not having to worry all the time…”  “Kind of take most side effects if it means that I can get my licence back and become seizure free and stop that worry… about fitting and, the side effects of tiredness and mood swings and irritability pale into insignificance compared to worrying about making an idiot of yourself in the town centre, by collapsing on the floor, or worrying about your daughters hurting themselves or not being able to drive.”  **Article 208** “I am currently undergoing treatment by a doctor. It is nearly 10 months I have not had any seizure.”  **Article 328** “I was fit free then they said they were trying to get women of childbearing age off Epilim, yeah, onto Lamictal or lamotrigine or whatever, so under the advice of the doctor at the Epilepsy Unit. I said, well fine, you know, as long as it doesn’t affect me.”  “Wanna change them I wanna come off them. I haven’t been happy ever since I’ve started taking them. I wanna come off them. I don’t think they’re the ones for me. I went to see xxx and said that I wanted to come off them. He said, ‘Do you want me to put you on a’.”  “There’s definitely been an improvement which is why perhaps I would with persuasion increase the new one to stop them altogether again I’m feeling okay at the moment.”  **Article 397** “Periods when I did not have seizures… three months could pass, half a year… then I was living… and then I thought well now the seizures have ceased, they will not return, it’s over…”  **Article 439** “I think everybody wants, well those who have got epilepsy, we want the end of it, I mean we don’t want to go through where one minute you’re fine, the next you’re not, especially with me having these children so young.”  **Article 441** “The medicine keeps me from having seizures, so I don’t have those times when I can’t concentrate after a seizure as often.”  “The medicine makes me a little groggy, but I’m not having nearly as many seizures, which totally wipe me out, so I feel so much more energetic than before. As long as I’m not having seizures, I can get a lot done.”  **Article 502** “And actually there was this lady who was giving me taweez and I went better for 1 year. I didn’t have a fit.”  **Article 528** “I haven’t seen a doctor for five years. I’m terrified that they’ll change things or start mucking around with me. As it is now, I’ve nothing and that’s how I want to keep it. I don’t want to go back to a fit every six or seven months.”  “I insisted I went there. The tonic-clonic seizures stopped. I was still getting queer sensations. They told me to take one more tablet and I’ve been seizure free for four years. I feel angry that I put up with someone who wasn’t an epilepsy specialist for 20 years.”  **Article 531** “We had about a month with no seizures. You think you have found a medicine that will stop the seizures. Then the honeymoon is over. Boom, back with a vengeance.” |
| Seizure injury | **Article 062** "Sometimes, when it comes, I hurt myself. This last time I hurt myself a lot. Here I have a bruise, see? I was taking a shower and it came. And I fell." "I hurt people, I kicked them because they didn’t know what to do." "Because I hurt myself so badly, here in the back of my head."  **Article 063** "He’d say, 'what have you done now?', 'I’ve scalded myself.'"  **Article 115 supplement** "it is a disease for which I don’t need to be taken to the hospital every time and because of which I am [usually] not injured."  **Article 137** "I ended up with a cracked vertebra and a fractured skull."  **Article 144** "Dealing with an injury as well as dealing with the seizure can be difficult."  **Article 216** "He has osteoporosis due to long term use of epileptic drugs, and has fractures during seizures. He has broken vertebrae and ribs during seizure-related falls."  **Article 285** "I’m more worried about like getting hit by a car during a seizure than I am actually having like an overloaded seizure."  **Article 310** "I used to have three jobs at that time, and as soon as epilepsy hit me I gave them all up — thought no, I can’t work, I am terrified in case I have a fit… because I mean when I fell on the concrete I split all my head open and I had to have stitches inside and outside, I mean, I could have died on that floor... I gave up all my three jobs, I had to give up my bike — and yes it was really… I don’t know really how I did cope really, because I felt so isolated." "And I’d wake up from another fit, on the settee or in bed, you know, an ankle plastered in hospital through falling, dislocated fingers where I probably held on to something or pushed back, and it was, it was like a cloud, black, black cloud."  **Article 351** "…I was in the bathroom in the morning… fell down… hit my head and [wife’s name] came and said ‘oh, you know, that’s a hospital job’… first time in a long, long time."  **Article 429** "I have heard about another boy in the village with epilepsy who had a seizure by the river, fell in and drowned."  **Article 440** "I could not control myself and got hurt sometimes."  **Article 455** "A couple of times I’ve been feeding my baby daughter and I’ve had a fit whilst feeding her and she’s fallen on the floor and hurt herself."  **Article 567** "‘Absolutely black and blue from head to toe.’" |
| Seizure frequency | **Article 216** "Seizure management has given us a bit of family life back. I can now have holidays. I can have a relationship with my partner. His sibling can have time with me now … It’s been completely life-changing. We are now a functioning family as opposed to a family in a state of constant medical crisis."  **Article 317** "With my meds I’m, let’s say, 'ok,' I rarely have seizures, and many of them are not generalized, but what I need is them to see me all right, that they see me healthy. What happens, is that all of them already know me, and they have fear of my seizures all the time."  **Article 339** "Thanks to this new approach, I feel calmer, I have experienced fewer seizures, feel much … I’m more stable, I ﬁnd."  **Article 369** "But I mean I’m twenty-two now, it’s been pretty much the same, since I was fourteen, ﬁfteen, but it’s been, it went from, on average to, every two weeks to every week and it can be anything from [em], half an hour, where I’ve woken up after one and gone into another, only happened twice though. From, if you like that, six days to two weeks [em] so after a week if I go for another week or two without the seizure."  **Article 384** "I have seizures fairly often so I simply don’t dare be on my own with my children." "It’s unbelievable sometimes. You’ve lain there shaking … and you’re exhausted afterwards. You’ve had so many."  **Article 402** "After starting treatment and medication at KCMC, the frequency of seizures has gone down from two or three a day to one or two a week." "I am very thankful for the medication because I have seen a great improvement."  **Article 441** "When I ﬁnally started on the medicines and began having fewer and fewer seizures, I just took a big sigh of relief." "I only have maybe one seizure a week now ... so now I can get almost all my work done during the week and not have to catch up on weekends."  **Article 456** "If she takes medicine regularly, she only has a seizure once a week or two weeks. Without the medicines, she would not be recovered and would have seizures more frequently."  **Article 502** "You’ll have three and you think, what was the point of taking that, it hasn’t made any difference."  **Article 531** "The medication doesn’t make me feel like myself. It makes me schizophrenic, basically. Taking the medication did not stop me from having the seizures. I am having just as many seizures then as I am now." |
| Experience of seizures (NOS) | **Article 063**  "Understand when a seizure takes place, you basically fall to the ground. That’s the situation. Erm, as I say, the muscles’ll jerk involuntary."  **Article 238**  "Being afraid of somebody seeing me having seizure, I take the medication."  **Article 294**  "… one of the biggest things now is her sleeping, not getting enough sleep, and because if that gets out of control, then that’ll cause more seizures … If I see that she’s forgetting to do something, that’s when I’ll step in, but for the most part, I try to let her do everything herself."  **Article 328**  "Tiredness and obviously the increase in the seizures … again its tiredness but … I gained a lot of weight."  **Article 397**  "…but then I had a major seizure when I was at a disco and was taken to the hospital by ambulance… and they thought at ﬁrst that I was using drugs… just think…"  **Article 403**  "the waiting list is crazy … I was housebound for a year and a half having drop outs and seizures and dropping conﬁdence … in my GP waiting room… in front of people."  **Article 441**  "I’m terriﬁed I’ll have a seizure in front of people, and who knows what I’d do during it. It would be so embarrassing."  **Article 449**  "had a seizure on the way [to clinic]. My children had to go back to the village for help and my family had to come and collect me in a scotch cart. My husband says he won’t divorce me as this problem started after we were already married, but one day he will tire of this and leave."  **Article 451**  "Then, after two or three years I was in that previous state when I used to keep on having fits again and again, I thought they weren’t working so I just took them off, just stopped using them."  "Well, you know, you take it (hakim’s prescribed medication) and you don’t have any fits for a week and then the next week you’ll have three and you think, 'What was the point of taking that, it hasn’t made any difference?'"  **Article 453**  "I’d rather take it (MEDICATION) than have the problems that seizures can cause." |
| Experience of seizure auras | **Article 062**  "I know it is coming, in general. What I am not able to do is to inform it."  **Article 074**  "I’m having auras, and… you know anxiety triggers auras, auras trigger anxiety."  **Article 115 supplement**  "I have the feeling that something is crawling up my back over my head and then into the inside of my head."  "This aura [‘the crawling up from behind and any images of my mother’] has no longer happened."  **Article 286**  "If I had a feeling that epilepsy is coming on, and I have thirty seconds…then I could sit down, but no warning… to say that it is coming now…that’s the worst feeling."  **Article 384**  "And then I had feelings of déjà-vu at a crossroads and I didn’t dare tell my husband in case he’d think I was an idiot." |
| Focal onset seizures without generalisation / impairment of awareness | **Article 158**  "Obviously not pleasant while she’s having them, especially because she’s aware, unless she’s had the full tonic clonic, and er, and so obviously when she’s having them we just talk her through it….mmm just keep trying, and obviously keep saying it’s not her fault."  "I know what happens, what happens to me, I go very quiet and I look down at my feet and I won’t be able to talk, I can hear you, but I can’t respond. And that’s when I know it’s…when I’m having a ﬁt."  **Article 169**  "Sometimes I think mentally you can control it anyway. I don’t know. ’Cause I think sometimes with my ﬁts, I might go like, I might feel it. Other times I′m not, I might just ignore it and don’t concentrate on it, and therefore, it’s not, I can’t feel it as much? My mind is preoccupied with something else."  **Article 369**  "Since then, I’ve had all the [um] partial seizures where my [um] arm shoots up and eye wanders and I feel very shaky for a couple of minutes, sometimes it rumbles or sometimes it’s just quick like that sort of thing."  **Article 384**  "And then I had feelings of déjà-vu at a crossroads and I didn’t dare tell my husband in case he’d think I was an idiot." |
| Seizure with impaired awareness, including generalised-tonic clonic seizures | **Article 062**  "But then I had a big seizure and I ended up in an ambulance."  **Article 115 supplement**  "I have not had any Grand mal ever since and so I am of the opinion that things are moving forward."  **Article 207**  "The generalised seizures are by far the most, the ones that have the most impact on my ability to function on a daily basis, the partials you can pass off sometimes as just depending on how long they go for… the generalised one, the recovery time as well is excessive for me, I need to sleep for hours, so, if that happens at work or when you are out, it is a, it can just ruin a day you just lose a day basically, which is not good for me."  "It’s looking down towards the surgery route now… the medications I have tried, you know, I have been up to and around and actually above the maximum dosages… the medications just don’t seem to have had an impact on the grand mal seizures, the full seizures… actually [I’m] quite pleased…"  **Article 369**  "So these are big diﬀerence in the types of seizure that can happen. [um] It was a big step for me, mild seizure to a tonic clonic." |
| Post-ictal drowsiness | **Article 062**  "When it comes, I want to sleep. Then I wake up and I can’t bend over, it’s terrible. I sleep, I sleep. That is my problem. I want to sleep."  **Article 158**  "She’s usually very confused, you know, she gets confused after, when she’s coming out of it and she does have like rasping, breathing, that kind of thing."  "And that’s when I know it’s…when I’m having a ﬁt, and when I’ve come out of it I’m really tired."  "It’s not difﬁcult ’cos I know what to do for it, I don’t like having epilepsy because it makes me have seizures and it makes me feel horrible after, I feel groggy, tired, and I can’t focus, I can’t focus when, when I’ve had epilepsy I need quietness, I can’t stand noise when I come out. And it’s horrible."  **Article 207**  "The generalised seizures are by far the most, the ones that have the most impact on my ability to function on a daily basis, the partials you can pass off sometimes as just depending on how long they go for… the generalised one, the recovery time as well is excessive for me, I need to sleep for hours, so, if that happens at work or when you are out, it is a, it can just ruin a day you just lose a day basically, which is not good for me."  **Article 441**  "For 24 hours after a seizure, I’m so exhausted I’m paralyzed and I can’t even concentrate and I get confused … I can’t get anything done, so I have to cancel everything for that day."  "The medicine keeps me from having seizures so I don’t have those times when I can’t concentrate after a seizure as often." |
| Seizure duration | **Article 115 supplement**  "Back then it used to last for a quarter of an hour and now it is maybe just about three minutes. So, (laughs), you have to be cool about it, that I have learnt then you get over these three minutes."  **Article 280**  "Yeah it’s not a nice thing, especially when it’s uncontrollable. . . when it goes on for a minute, a minute and a half, you honestly think you’re gonna die. . ."  **Article 369**  "But I mean I’m twenty-two now, it’s been pretty much the same, since I was fourteen, ﬁfteen, but it’s been, it went from, on average to, every two weeks to every week and it can be anything from [em], half an hour, where I’ve woken up after one and gone into another, only happened twice though. From, if you like that, six days to two weeks [em] so after a week if I go for another week or two without the seizure."  **Article 384**  "Seizures are really nasty, they last 10 minutes maybe 15 and then it’s over." |
| Difficulty with breathing during seizure | **Article 063**  "I cannot breathe, my muscles are twisted, em… I’m gasping for breath and that’s just before I lose consciousness."  **Article 158**  "When she’s coming out of it and she does have like rasping, breathing, that kind of thing."  **Article 369**  "I’m, it feels like I can’t breathe, [um] my lips turn to blue, and I sweat you know quite a lot and [um], yes so those are the two main things I can remember at the moment." |
| Loss of motor control during seizures | **Article 115 supplement**  "You know exactly everything that is going to happen now, you will likely not be able to control."  **Article 137**  "It was the oddest sensation because I was here while the incident was going on. But it wasn’t me that was laid on the ﬂoor."  **Article 369**  "I was, but I felt like I [um], couldn’t control my limbs, I felt like sort of my body almost became, almost became a dead weight, and I [um], it felt like my insides sort of turned literally upside down, and this feeling like I was just going to die, and this sort of weird, this is the bit that’s really hard to explain because I don’t really remember afterwards." |
| Post ictal symptoms – other | **Article 137**  "The worst part of having a seizure is not the seizure itself, that’s the bit that’s alright, it’s afterwards (...) everything is a bit like a balloon that’s burst."  **Article 158**  "She’s usually very confused, you know, she gets confused after, when she’s coming out of it and she does have like rasping, breathing, that kind of thing."  "It’s not difﬁcult ’cos I know what to do for it, I don’t like having epilepsy because it makes me have seizures and it makes me feel horrible after, I feel groggy, tired, and I can’t focus, I can’t focus when, when I’ve had epilepsy I need quietness, I can’t stand noise when I come out. And it’s horrible."  **Article 216**  "One of the greatest worries for us are the after-effects of the seizures – she is unable to communicate and is in high state of tension afterwards for 24 hours. She is not able to eat, sleep and relax during this time." |
| Ability to identify seizure triggers | **Article 115 supplement**  "The search may be successful, but […] once I have the result ‘That could have been the trigger’ it is not always that easy to change it so that it does not happen again."  **Article 294**  "… sometimes we try to figure out stuff and see what may have, like I said, triggered something. And most of the time we can take it back, you know, to something that may have happened or if, you know, I forgot to take medicine, something like that." |
| Post-ictal duration | **Article 062**  "It’s like I’m lost in time and space. I don’t know. I have a hard time to come to my senses."  **Article 207**  "The generalised seizures are by far the most, the ones that have the most impact on my ability to function on a daily basis, the partials you can pass off sometimes as just depending on how long they go for… the generalised one, the recovery time as well is excessive for me, I need to sleep for hours, so, if that happens at work or when you are out, it is a, it can just ruin a day you just lose a day basically, which is not good for me." |
| Experience of myoclonic jerks / myoclonic seizure | **Article 310**  "But even them [seizures] just the changing from the before seizures to, like, the myoclonic jerks — that made a difference as well because I wasn’t quite as scared, I felt more that if this was happening, well, this was alright, I could sort of deal with this."  **Article 369**  "It was, they [myoclonic seizures] were just both scary." |
| Seizure related loss of awareness | **Article 062**  "This is the thing. I don’t know what I do. Everything I do [during the seizures], someone tells me about it."  **Article 063**  "The very worst thing is the feeling that you have absolutely no control over, your mind, your body that’s bad enough, but the fact that you are totally unable to control what’s happening in your head is absolutely terrifying." |
| Seizure severity | **Article 136**  "I was probably in my mid-thirties, but they were very minimal so I didn’t recognise them as seizures, and neither did my doctor… In the meantime, the little seizures grew very much more frequent and probably even more deﬁnite till one morning my husband woke up and my eyes were rolling in my head…"  **Article 369**  "It’s been a quick jump from a small seizure to a very big seizure." |
| Status epilepticus | **Article 058**  "I stayed in status epilepticus for 2 hours when I had my meds with me in my purse."  **Article 369**  "But oh I’ve also got, I also go into status (epilepticus) a lot, so that’s not a good thing to be doing, you know, I would go into seizures and I would need to go into hospital to be brought out of them, [um], and quite often I wouldn’t be able to maintain my own airway, so that’s all a bit scary too." |
| Unpredictability of seizures | **Article 286**  "If I had a feeling that epilepsy is coming on, and I have thirty seconds…then I could sit down, but no warning… to say that it is coming now…that’s the worst feeling."  **Article 332**  "A patient with epilepsy may face an uncontrollable situation without knowing the outcome. For instance, at any moment I could have a convulsion in public and suddenly fall on the ground where people gather around me and witness the event. I hate it when people say mean things to me." |
| Brain injury following prolonged seizures | **Article 369**  "A vegetable basically. And I wasn’t, wasn’t afraid of dying really, I was afraid of that because I always thought if I have a seizure and I die I don’t know about it, that’s it, but if I’m in a wheelchair or I don’t want my life ruined by it."  "That very much concerned me at the time, was the fact that with every seizure that is had, [um], it [um] something is diminished, destroyed, in the brain, as in progressive, progressive fault, harm to the brain."  "I didn’t get this under control I really wondered where it would leave me, [um], in a few years’ time. I don’t want to say, ‘vegetable state’. But the brain is such a delicate thing." |
| Cognitive symptoms during seizures | **Article 115 supplement**  "Here, I already know: It depends, if you allow it, the speech loss will then come. And then I am determined not to allow it." |
| Emotional distress during / after seizure | **Article 369**  "I don’t think it lasts for any longer than maybe 30 seconds itself, as well, but at ﬁrst you sort of feel very frightened and you think, ‘Oh, what is happening?’ because it’s all visual." |
| Experience of sensory phenomena during seizure | **Article 369**  "I was, but I felt like I [um], couldn’t control my limbs, I felt like sort of my body almost became, almost became a dead weight, and I [um], it felt like my insides sort of turned literally upside down, and this feeling like I was just going to die, and this sort of weird, this is the bit that’s really hard to explain because I don’t really remember afterwards."  "And I sort of get [um], kind of a vision almost, kind of, almost hallucinates, and [um] the, the things I see are always really terrifying."  "And I feel very afraid and like I want someone to help me, and [um], just this really sinister feeling, and then, very suddenly my sort of insides turn the right way up again and I feel like I’m coming out of it."  "I don’t think it lasts for any longer than maybe 30 seconds itself, as well, but at ﬁrst you sort of feel very frightened and you think, ‘Oh, what is happening?’ because it’s all visual." |
| Motor symptoms of seizure | **Article 115 supplement**  "Everything still works […]. I think normally, I can move my body absolutely normally, except my face as it pulls to the left and it feels as though you cannot breathe properly and that produces such hissing noises." |
| Only focal seizures (without generalisation / impaired awareness) | **Article 317**  "With my meds I’m, let’s say, ‘ok,’ I rarely have seizures, and many of them are not generalized, but what I need is them to see me all right, that they see me healthy. What happens, is that all of them already know me, and they have fear of my seizures all the time." |

B. BEHAVIOURAL AND PSYCHIATRIC OUTCOMES

| OUTCOME CODE | VERBATIM PARTICIPANT TEXT |
| --- | --- |
| Symptoms of depression | **Article 136**  "There were feelings of anger, but they became feelings of depression."  **Article 139**  "It’s really difﬁcult to get motivated to do anything, or to even take your medication when you’re depressed."  **Article 188**  "How I’m feeling in myself at that moment, yes. If I am alright, and sometimes I am really depressed, you know what I mean, it’s like how I am feeling about myself and how I would be if I didn’t have epilepsy."  **Article 216**  "Our child now realises the extent of the impact that TSC has had on not only our child’s life but also the lives of everyone else, and because of this has had a few psychotic episodes, becoming very depressed during these times."  **Article 310**  "I think the other thing with the seizures, it’s the side effect is you don’t feel so confident within yourself, it affects your perception of yourself. I was very negative, I got very depressed, well when I say very depressed, I don’t mean so I became a depressive, but I didn’t know when I was going to have a fit, so I was always thinking about that, it was always on my mind."  **Article 317**  "And the attacks, the visits to the doctors, the meds, everything. I became so depressed that I didn’t want to go to high school anymore. At jobs it’s the same…what lowers my mood is to try many times, and again and again, and sooner or later you get ﬁred from all. For me, it’s enough!"  **Article 320**  "I now am not real able to do what I need to do. I could sit here… and not move. I’m exhausted and depressed."  "I told my [friend] I didn’t know how long I could go on…tired and depressed. She said let’s call your doctor…now I have anti-depressants."  **Article 328**  "I’m better in that it has the jerking thing has got less … [but] generally [the] depression is getting worse."  **Article 369**  "Kind of develop more and more paranoia, depression, all that sort of thing that goes with it as to ‘come on, you know, just have the ﬁt so I can get back to normal lifestyle’."  **Article 403**  "Tortured (by co-workers) and felt suicidal … everything was getting on top of me. He ‘credits’ the Epilepsy Association for ‘helping me overcome it’."  **Article 441**  "I’ve been so depressed since I found out, even though it was nice to have a name to it." |
| Poor memory or concentration | **Article 062**  "Memory… I don’t have memory any more. I don’t remember things. If someone tells me, ‘do you remember this?’ No. ‘Do you remember this person?’ No."  **Article 115 supplement**  "Before I had epilepsy, I could have sworn that I do not need a calendar. But now I check all the time […]: Is there anything that I missed?"  **Article 188**  "Apart from the definite effects it [epilepsy] has on memory, it really hasn’t made a fantastic difference."  "I was worried because my memory did get really worse very quickly … not being able to remember the kids’ names is a big problem. You know, I ask them their names and then 10 minutes later I have forgotten it and so you can’t let on you are forgetting… My quality of life would go up, my happiness would go up by reducing the stress."  **Article 207**  "Next I would put problems remembering things, because that really does affect work and it affects home life as well—you know, when I am supposed to do things, just everyday things, and I forget to do it, it has a huge impact… if I forget things in work it’s serious… God, something could go wrong, and something bad could happen… and all because I forgot to do something. So that does worry me…"  **Article 320**  "The worst thing has been my memory. I can’t keep track of anything…. It makes everything embarrassing."  **Article 332**  "Because of epilepsy I thought my memory was failing and I was having amnesia. In the ﬁrst and second year of school, I had the highest grade point average (GPA), but later my average declined and I graduated with a 14/20 GPA on my diploma—all because of having epilepsy. I still worry that I may forget all the information in my brain because of convulsions and I fear this will happen."  **Article 339**  "The thing I ﬁnd almost the worst is my memory problems. Because epilepsy is a little more concrete—you have to rest a bit after a seizure. But going around with memory problems all day long takes a huge amount of energy; it drains your energy, I think."  "I had a lot of problems with the cognitive impairments. It wasn’t possible to study. I couldn’t, you know, prepare meals. I couldn’t follow a recipe."  **Article 384**  "Yes, my memory is pretty bad. So … it’s not much fun. That’s what has been bad at school, so I was also given, also given one of those extra guys to help me."  "If you tell me two things, I can remember the ﬁrst. But my wife sends me to buy this, that and the other at ICA. If I don’t make a list, that’s it, I have to call and ask."  "I’ve found it hard to concentrate. Mmm, if I’m at a meeting for example, I can’t concentrate for more than maybe 5 minutes if it’s something I really have to pay attention to because I have to report back to others and so I sit there with my notepad and I start to write and then just, no, and I start doodling, drawing stuff, ﬂowers."  "[E]h my memory … it’s a little scary because things can happen."  "I’m thinking about my memory. It’s becoming worse and worse and worse."  "Fun things like phoning a friend who I’ve promised to phone, disappear too. ‘Call me tonight, yes, I’ll call.’ And then two days later suddenly … of course, I was supposed to call her. That’s happened more than once when someone asks me to call them or do something. My memory has really been affected recently especially."  **Article 429**  "My daughter passed all her exams but her memory is not as good - when she answers the phone she cannot remember anything about the conversation."  **Article 532**  "My mind isn’t as clear as it ought to be."  **Article 540**  "My mind isn’t as clear as it ought to be." |
| Anxiety - general | **Article 074**  "But I always worry, I clock-watch at night."  "So although you have epilepsy, the big thing that’s with you every day is the fear."  "Umm, I get anxious. I don’t take stress very well. Anxiety and stress will increase my seizures."  "It was horrible because there was always this constant, underlying fear."  **Article 103**  "When it got to that point I thought about killing myself. The anxiety got that bad."  **Article 115 supplement**  "[My husband] says I clench my thumbs which indicates that I am tense once again."  **Article 136**  "Feelings of anxiety, feelings of isolation."  **Article 310**  "The anxiety bit is the biggest problem I have had with all of this, and then I went on this workshop with the lady at [Medical centre] … it was absolutely fantastic … the strategies and the relaxation techniques, to try and curb these sort of panicky anxiety things, was massively helpful."  **Article 320**  "Basically, he said that people…with seizures have anxiety. It’s part of it and I have to try to relax."  **Article 460**  "I thought epilepsy was going to complicate my life over the years. I felt anxiety every day and I was afraid to lose control."  **Article 528**  "That’s the vital time. I remember saying I was frightened and he said, ‘Everybody is!’ It was a bit of brush-off." |
| Satisfaction with knowledge about epilepsy | **Article 156**  "I think just like the knowledge to know really and to be prepared almost I guess."  **Article 079**  "They haven’t asked us how we are doing and about our health status. They just gave us the medication and we went to our home. There may be a problem on this.… It would have been good if they had given us [advice]. …We can protect ourselves. We can tell other patients who haven’t accessed the service here about the illness and other things."  **Article 084**  "I believe that knowledge obliges, and that knowledge … I wonder why they don’t inform us as soon as possible in our disease process."  **Article 136**  "So, I would always advise people to just get a book, do a bit of research, talk to somebody who has it and who has dealt with it well over the years and just get a bit of support to talk about it so that you can very quickly learn to accept it."  "When I started having the seizures, then I kind of thought, yea I’ll, I’ll look into it; kind of to see what I have to do to kind of not do it (have a seizure). To see to it that, I kind of stay away from certain things. So that’s kind of what I had to do."  **Article 137**  "I don’t know, I don’t know how it all works."  "When you come to terms with epilepsy you come to understand it, investigation gives you better understanding of it, so I can say I could spend two hours on a computer screen reading up epilepsy."  **Article 144**  "The consultants they just presume that you know [about epilepsy]…but for all the years he got put on tablet or tablets…you didn’t see or hear any of this [information presented by course]."  **Article 146**  "The programme has helped me to understand more about epilepsy and medication. I take my treatment more seriously now."  **Article 158**  "Have you ever been kind of given any kind of information about epilepsy, not the book, anything before that? Have you ever had any leaflets about epilepsy? Not really, no. No, so no easy read or anything?" |
| Suicidality | **Article 079**  "And I was thinking like, why don’t I strangle myself rather than falling down into an abyss and being eaten by a hyena... ehh… or rather than drowned in water… ehh… I said it’s better to strangle myself."  **Article 103**  "When it got to that point I thought about killing myself. The anxiety got that bad."  **Article 238**  "I am often thinking of marriage and the fact that I am suffering from epilepsy, whether I get married or not, whether my secret is exposed or not weighs heavy on my mind. At least three or four times I have attempted suicide. I am very tired of life. I often beat myself."  **Article 332**  "I went on the roof with intent to jump down and kill myself."  "I wanted to jump off the roof and end my life when suddenly someone took my hand and did not let me jump."  **Article 403**  "Tortured (by co-workers) and felt suicidal … everything was getting on top of me. He ‘credits’ the Epilepsy Association for ‘helping me overcome it.’" |
| Anxiety symptoms from seizure related uncertainty (seizure worry) | **Article 136**  "I had no idea what it was, so it terriﬁed me, I was really scared, I was quite upset."  **Article 216**  "It makes me scared to go out. It is very hard. I don’t know when I am going to have a seizure – they come out of the blue. I don’t even want to go and get a cup of tea."  **Article 328**  "It never used to bother me really … but now I feel a bit more nervous about going out and doing things by myself."  **Article 332**  "A patient with epilepsy may face an uncontrollable situation without knowing the outcome. For instance, at any moment I could have a convulsion in public and suddenly fall on the ground where people gather around me and witness the event. I hate it when people say mean things to me." |
| Sense of hopelessness | **Article 074**  "It feels sort of so hopeless that I can’t really articulate it. The future won’t be remarkable."  **Article 238**  "Sometimes I get very tired and sometimes I am discouraged about life, because I have not recovered from this disease."  **Article 320**  "I feel useless now. I sometimes feel like I don’t have anything to give back."  **Article 397**  "… then I must say that I gave up… and what is the point of living and so on you know… then I somehow lost faith…" |
| Safety consequences due to poor memory | **Article 207**  "Next I would put problems remembering things, because that really does affect work and it affects home life as well—you know, when I am supposed to do things, just everyday things, and I forget to do it, it has a huge impact… if I forget things in work it’s serious… God, something could go wrong, and something bad could happen… and all because I forgot to do something. So that does worry me…"  **Article 384**  "It is scary when you switch on the cooker or the iron. There have been times when I’ve left the iron too close to something, like a plastic spray bottle and, of course, that melts!" |
| Word finding difficulty | **Article 074**  "When you almost have the word on the tip of the tongue, it’s like that constantly and it’s horrible. I was at work yesterday and […] I couldn’t think of the word glove, I probably looked like an idiot."  "I can explain it 100% but I just can’t think of the word. It doesn’t happen all of the time, it may happen once every two or three days, if that. And then when I’m doing it I’m like ‘ohhhh’ (clicks ﬁngers), so now it’s like a game, and people help me out, they jump in with a word, and I’m like ‘oh yeah that’s it!’"  **Article 384**  "This thing that words disappear, talking about something, if you skip a word in a sentence, I, like, can’t think of the word but I know what I actually want to say. Have to almost explain around the word so that the other person can say which word I want to say. And that’s really hard."  "Forget words, I ﬁnd that really hard, also, what does the word mean? … Worst of all is really the words I always know … which I’ve always known, so to speak." |
| Impact of poor memory on medication compliance | **Article 384**  "And then I run out of medication and I go and order more on Monday and know it’ll arrive on Wednesday and still I forget to collect it on Wednesday."  "The medicine, every day at 8, but when, on a day I have got stuff to do I take it at 7 instead, but then at 8 I think oh my God, haven’t I taken it, and I take another one. It becomes a complete mess."  "But I’m almost senile so I put two in each compartment so I’ve got enough for, say, 4 weeks. And then you forget which strategy you’re using to empty the compartments. That’s not good." |

C. SLEEP OUTCOMES

| OUTCOME CODE | VERBATIM PARTICIPANT TEXT |
| --- | --- |
| Adequate sleep quality and duration | **Article 294**  "… one of the biggest things now is her sleeping, not getting enough sleep, and because if that gets out of control, then that’ll cause more seizures … If I see that she’s forgetting to do something, that’s when I’ll step in, but for the most part, I try to let her do everything herself." |

D. PHYSICAL FUNCTIONING AND DISABILITY OUTCOMES

| OUTCOME CODE | VERBATIM PARTICIPANT TEXT |
| --- | --- |
| Independence (or loss of independence) | **Article 062** "Then, when I was in primary school, in second grade, they said I had to go to another school because I didn’t have high grades, because I was distracted..."  **Article 208** "I am excluded from what happens in the family. I have even been forbidden from managing my income on the ground that I am unable to do it."  **Article 294** "It kind of doesn’t let me be on my own." "I get aggravated because [my mother is] always around, helping me, and I just want to do everything on my own. But I understand that I can’t now, and she’s there for me. But, yeah, I get aggravated sometimes. I get tired of her always helping me, but I know it will get better."  **Article 310** "I was getting up to 11 months [seizure-free] and then I had another one. That was so depressing because I couldn’t drive, that is like, if you can’t drive, well to me that was my independence. I couldn’t go nowhere, that is the way I felt, you know."  **Article 328** "It never used to bother me really … but now I feel a bit more nervous about going out and doing things by myself." "I just never really settled with them and when I had the big fit then I thought God I can’t really trust myself to go out on my own you know."  **Article 449** "This disease has caused me a lot of problems. I can’t walk around freely. I can’t cook because of the fire. I am not free to do things since at any time I could have an attack. I am not even free in my own home. I can’t go to draw water alone. I can’t bathe alone. All diseases hurt, but this one has really affected my heart."  **Article 453** "... like I’m going on holiday and I’ve put off coming off them again until I come back because I’ll have to get watched in the swimming pool, and everything." |
| Ability to go swimming | **Article 074** "I’ve always just gone and seen the lifeguards and said ‘I’m epileptic’ and swum between the flags."  **Article 136** "If I was letting it control me, I wouldn’t have been swimming, I wouldn’t have done anything like that."  **Article 169** "I hate that I can’t just drive and just get home. The same from swimming…. If I could get to swimming events on my own …. But I don’t, I have to rely on everyone."  **Article 286** "I couldn’t go swimming; I couldn’t play computer games, bike riding. I couldn’t go too far because my parents were worried about where I was going and if I had a fit."  **Article 332** "With him as much as possible because I must keep my focus and attention on him. It is difficult! He does not go hiking and swimming often, and the last time he went swimming with his father, he had a fit in the pool, and his father did not notice it. Another time we went hiking together, as we often do, we did not let him climb the mountain alone." |
| Ability to exercise | **Article 074** "And yeah, avoid doing things like that. So I try not to exercise. I don’t even go for a walk on the beach anymore, which sucks because it could probably help me." "I’m doing exercise walking to the bus and train station and then to work."  **Article 115 Supplement** "[T]his one dance school does […] a dance evening […]. And then you unwind […]. That works."  **Article 169** "It’s very important to me to keep fit. It always has been. When I was at school, I was really, really, really… fit!…. Like 2, 2 years ago…it’s a bit more…. 'If you don’t do exercise, that’s fine.' But now, I′m so, so keen to keep fit ’cause I′m in my late 30s, and I really don’t want to [laughs], not do anymore exercise." "I′ve started to push myself too much in the gym, and I will feel like really tired afterwards but then feel a bit better later. But then mostly, exercise makes me feel better straight away, a bit more energized … and I never actually felt twitchy in my head during exercise." "It’s my epilepsy that seems to be making my decisions when I can exercise. Which I find very, very frustrating. I′d love to say I′m going out for a run tomorrow, but you know, if I have a seizure, I can’t do that. I don’t know whether I will be well tomorrow, that’s what I feel frustrated about. I can’t make any plans…" "Umm, like everyone has told me to stop. And it’s annoying. It’s like, I can’t [long pause]. I′ve loved sport since I was a kid, and I′d always played football, and always played tennis, and always been part of team sports, and if it’s a new thing, I′d want to take part in it. And being told not to isn’t [laughs], just isn’t … [something I′m prepared to do]." "My sports, when I was younger, were BMXing and downhill mountain biking, and now, it’s gone to lifting a weight in a bedroom. It’s kinda like… but, if that’s what it has to be, that’s what it has to be until there’s a cure …. My stress levels have gone up because I′m not exercising." "I think I′d struggle if I was told to stop exercising. I probably would exercise anyway, to be honest … I don’t know…"  **Article 188** "I have had everything there is to have, kids that I have done a lot with, sport, everything else, and I don’t really think you can have a better quality of life, to be quite honest." "If you listen to everybody who says don’t do this, don’t do that, you would stay in the house and call it a day. And, I am not willing to go out like that, so I do weights, walk every day with my dog, walk most places, and I go out a lot." |
| Sexuality and sexual functioning | **Article 084** "It’s just as important to discuss how my sexuality is affected. Maybe it won’t be affected, but it’s a possibility and there are ways of treating it." "I didn’t know that epilepsy could affect my sexuality." "Today I don’t just throw my wife on the kitchen table. Something holds me back … or I lack something …" "That’s why we are still together. She tells me that it is me and not my penis she cares about." "I need to be strong for my wife. So she can feel that I can carry her if it is necessary, and ‘do’ her. And about having an erection …" "I’m honest with my boyfriend about being too tired for sex." "You mentioned orgasm … this is something I only started allowing the past couple of years. Because I lose control … I think ‘what if I suddenly have a seizure?’"  **Article 185** "For long, I told them about my manhood problem… I have since given up on them… They did not care…" "I wanted to know what causes that. If I want to meet with her [has sexual desire for his wife] … then the lights turn off first very quickly [has difficulty in maintaining an erection] … it wasn’t like this before…"  **Article 230** "When you write that you have lost your libido and you have gained weight, then what happens?"  **Article 449** "When you are having seizures, you don’t have desire for a man." |
| Ability to do gardening | **Article 188** "But you do it [working on allotment] because you enjoy it, it’s fresh air and you switch off, you do switch off…. It’s probably one of the best things that I ever got into, yes very good for you, so yes, that’s a really good de-stressor, for want of a better word … I think also some feeling of pride in the sense of what you have done, you know."  **Article 332** "Sometimes I want to do some gardening, water the plants with my father, but my mother would not allow it and wants my father to do it, thinking I may get hurt. I do not like these restrictions." |
| Ability to exercise alone | **Article 169** "I think … if I run with my husband, then, uh … yeah, I guess I feel a bit more supported. If somebody’s with me, you know, running with me, then I have that added security in the case that if something did happen, then that’s fine. But it doesn’t stop me from doing it on my own. If I did an hour’s run or an hour and a half’s run with him, if it’s particularly strenuous, I don’t think it would stop me necessarily from having a seizure the following day, just because I had someone with me at the time … he doesn’t offer [to go on a run] in terms of feeling insecure about me going on my own, no … not at all. No, he knows I can go off on my own. He has no problems at all. He has got confidence in me. Knows I′ll be fine, I′ll be okay. In fact, if things were to happen, it’s usually post-exercise anyway." "You’re doing this, and you shouldn’t really be doing that. It makes me think, 'Yeah, maybe if I do go swimming or the gym on my own, maybe I should go tell someone?' But I don’t want to tell someone that I′m swimming because then I have got to have some idiot watch me swim, and I don’t want that."  **Article 332** "With him as much as possible because I must keep my focus and attention on him. It is difficult! He does not go hiking and swimming often, and the last time he went swimming with his father, he had a fit in the pool, and his father did not notice it. Another time we went hiking together, as we often do, we did not let him climb the mountain alone." |
| Ability to go shopping | **Article 074** "If it was back in the day it would be a whole lot harder, but now you can do everything online, so the groceries get delivered online." |
| Ability to take part in team sports | **Article 169** "Umm, like everyone has told me to stop. And it’s annoying. It’s like, I can’t [long pause]. I′ve loved sport since I was a kid, and I′d always played football, and always played tennis, and always been part of team sports, and if it’s a new thing, I′d want to take part in it. And being told not to isn’t [laughs], just isn’t … [something I′m prepared to do]." "You could get annoyed by this, blah, blah, blah. Umm, football I had to give up because it’s in * and traveling there and back is dangerous, so she said, 'Give that up.' Two things, sporty things in my life that I really looked forward to each week, three sessions a week, that I really liked, have completely disappeared." |
| Ability to go to dance classes | **Article 384** "I have always had a great passion for dancing and I still have. But when the epilepsy took over and I had seizures at dance class… I felt that I couldn’t go on, so I dropped out." |
| Ability to ride bicycle | **Article 286** "I couldn’t go swimming; I couldn’t play computer games, bike riding. I couldn’t go too far because my parents were worried about where I was going and if I had a fit." |
| Ability to go hiking / mountaineering | **Article 310** "Well in terms of quality of life, one of my great outlets as a hobby was mountaineering… So I then got this sort of list of things that I couldn’t do, mountaineering was ruled out straight away… so I would say that a major, major part of my quality of life was stripped out almost straight away because that was a sort of major outlet." |

E. EMOTIONAL FUNCTIONING OUTOCOMES

| OUTCOME CODE | VERBATIM PARTICIPANT TEXT |
| --- | --- |
| Perceived stigma or discrimination | The file is quite extensive. I will start by processing the first few articles with their references in the requested format. Let me know if you want me to continue for the entire file or target specific sections.  **Article 062** "Once, I was standing in front of my house when a neighbour passed by. ‘You’re lucky,’ he said with irony, ‘you’re at home all day.’ I got really mad. I insulted him. It came from my guts, all my anger. So, I said to him, ‘stay away. I can’t work because of this [my seizures],’ and I tried to explain to him. So he apologized: ‘I’m sorry, I didn’t know.’ People talk without knowing…" "They saw I had seizures. And the kids who saw me, the day after ran away from me: ‘Here comes the crazy lady,’ they said."  **Article 069** "Anytime I had a seizure outside my home, people came around to have a look at me and ridiculed me. None of them offered assistance until I became conscious… for me, this is stigma and discrimination." "Sometimes, I experience seizures outside home, and people around tell me, after recovery, how dangerous it was. I feel ashamed and uncomfortable when I pass by the same route of the incident the following day. What makes the situation worse is that I hear people talk and point fingers at me in reference to the seizure incident." "Besides, they believe I would not perform up to expectations or that being an epileptic, I am incompetent." "I did not disclose my health status before I got the job and I still have not. If I did, they would not have employed me." "No lady would marry me with such a burden. I had a girlfriend who did not know I had epilepsy. When she became aware, she ended the relationship. I don’t think I would get married in the future." "I don’t disclose my condition to people. If they get to know, they will shun my company." "I have decided not to get married again to avoid troubles from any man who might marry me and divorce me later upon realizing that I have epilepsy."  **Article 074** "I always find it awkward telling people, I’m very embarrassed about it […] And then you think will they treat you differently once they know." "I’m not ashamed of it anymore, so I will tell them that I’m epileptic. […] I suppose it was just fear when I was younger." "At first I found it quite hard that I’d been diagnosed as epileptic. I know when I was a kid it was considered […] something to be regretted and not talked about."  **Article 115 Supplement** "There has always been this question bothering me, ‘What will people think?’"  **Article 120** "Co-workers had doubted his capacity and ‘disagreed when management gave him the worker of the month award.’" "Supervisors had brushed off Lameck in favor of ‘the next guy.’"  **Article 136** "Felt I might be ridiculed, and I felt people might laugh at me, people might treat me differently."  **Article 137** "They think you’re (laughs), you know, crazy and eh, that’s awful."  **Article 139** "[You] get shut in because the outside world doesn’t want to see you anymore. That hurts." "A lot of people are going to have bad thoughts."  **Article 140** "I can sense it. With some of them, yes. I notice that they get away from me. When they realize I’ve got it, they feel suspicious and get away from me."  **Article 151** "Other terms like 'epileptic' and 'epileptic person' create a stigma as people are labelled with having a condition and are seen as the same as everyone else that has that same condition even though we all have different personalities and experiences in life which shape who we are."  **Article 169** "You can see a few people are worried by it and don’t want to talk to me and don’t want to be next to me ’cause, in the circuit training, we go round and round from point to point. And you can see their hesitance being in front of me or being behind me ’cause they will be the person, the first person to come to blows, so to … and … [laughs as he says the sentence] they won’t speak to me for the rest of the evening."  **Article 185** "You see the others look at you as if you are a grave… they just glance at you… even when you talk to her."  **Article 204** "People have a series of false beliefs in their minds. Some people are afraid of epileptic seizures, they think these patients have risk for them, or their disease is contagious, people avoid us because they don’t have enough information."  **Article 205** "My mother and father tried to hide my disease a lot; it is not good for a girl to have any defects."  **Article 208** "As epilepsy is a shameful disease, the epileptic individual is marginalized. He cannot enjoy the same rights as healthy people." "I am not respected. I do not have the right to go to public places. I have been given a separate cup and plate. They do not want me to get married…"  **Article 238** "When a person is having convulsion or fit on the street, people think he is acting or asking for money like a beggar." "Epilepsy should be called a hidden disease. I do not have as much problem with my disease as the community portrays."  **Article 253** "We keep our epilepsy hidden ... as much as possible."  **Article 280** "[The epilepsy is] still pretty much not spoken about... I was really surprised! I don’t know why, there’s obviously quite a stigma out there."  **Article 286** "The shame on me, it was too much [on her] and the name on the family…. Another family doesn’t want anything to do with your family." "If I keep having fits, my dad or my step-mother, they put me in a kind of small cage… they closed the door. I would stay there for two days. Without eating, without nothing because… they don’t want to see me having the fit." "A girl of your age doesn’t want to come around you. She believes, 'If I go around, playing with her, then I’m going get what she’s having.' Parents do tell their kids, 'Don’t go there.'" "Nobody want to come you, you can stay there whole day hungry and no one in the community want to come and run to you because they’re always say that you’re going to give them, you’re going to transfer the illness to them." "There’s certain times when I concealed it for a time…. For example, going for a job… I would conceal it up until the point where they make that decision. Because although… you’ve got the disability discrimination act…"  **Article 310** "I have had loads of fits in night clubs, and they kick you out like you’re a drunk or have been taking drugs … I haven’t done anything wrong like, they’ve just dumped me out here."  **Article 317** "But they do have fear, all the time! And that is why they reject me, they prefer me not to be there." "Some people come close to try to help 'Do you need something, here’s some water, do we call an ambulance? … to give support, to help'; but most of them get away, because they get scared I suppose, they feel strange and they just move away with disgust. People must think that he is dangerous, mad or maybe that he is dying! Or possessed by some demon! Sometimes I feel that they think that he can infect them with some disease."  **Article 332** "My whole world crashed and I knew that for the rest of my life I have to live in shame with a social stigma." "With my first seizure attack, my whole world crashed because people look at an epileptic person very differently." "Others which could bring me shame and disgrace. This could make me feel ashamed every time I see them." "Before I was diagnosed with epilepsy, I had the same view of the disease and considered patients with epilepsy as insane. I thought having fits happened to a group of insane people locked up in a room breaking doors, shattering windows, and screaming." "This is how they looked at me, as a weak or incapable person. My friends and those around me had an attitude of pity, and their behavior conveyed that I was a weak person." "They do not look at us like any other patient. They think that we are helpless, unable to cope with our disease or control the situation we live in. I do not know (pause)… for example, my maternal uncle knows about my condition and my sister’s problem, but he still behaves badly or in a crowd would say, 'because you have epilepsy, you wouldn’t be able to do this and that.' It is very degrading, and I want him to know I can do many things just as good as others."  **Article 384** "[A]nd then I had feelings of déjà vu at a crossroads, and I didn’t dare tell my husband in case he’d think I was an idiot." "Say you meet some new people that you haven’t met before and they find out you have epilepsy, well then they take three steps back."  **Article 402** "At school sometimes classmates are unwilling to be with me because they are afraid of witnessing a seizure." "I am afraid because people say if you are epileptic, you will in the later stages become mad." "My neighbors look at me like a mad man. They think I am dying, and they don’t want to be close or to work with me. They say I am taahira 'mentally unwell.'" "My working relationships are not good because my workmates always say I have mental problems." "I feel ashamed because men have forcibly impregnated me three times, but no one will marry me, and they don’t want to support their children." "Several times men have approached me for marriage, but they never come back again. I have come to realize that the source of the problem is our neighbors who tell them that I am epileptic and they should not marry me. I really feel very bad."  **Article 440** "People said it [epilepsy] was because we lived in a campo santo, an unsafe environment."  **Article 449** "Most of my problems are due to people talking about me. I would like to marry again, but whenever someone shows interest, people talk to that man and tell him about my problem, and so I haven’t had any offers of marriage." "My husband supports me. But since he took a second wife, he never lies with me. My husband’s family and the second wife are afraid she will get this condition from me. They think my condition shames the family. People tell my husband he should abandon me. Sometimes I drink." "In town there was too much talk, and even the children ridiculed me there. Then the children in town disrespected me, and I cried a lot. I became so sad that I could barely force myself out of bed to do my duties."  **Article 455** "Don’t mention fits or disability to other Asians. Yes, you only have to be, say, a disabled child or something, they think, you know, they’re sort of outcast." "I think … this disease got a bad reputation in our community. If you got epilepsy, you know, you’re not a normal person. … You can have a heart attack, 'Oh, you had a heart attack.' But, if you’ve got epilepsy, there is something—how can I put it?—abnormal. Because heart attack, you don’t call yourself disabled but, if you’ve got epilepsy, people say, 'Oh, something wrong with him.'" "With the epilepsy, I’ve been turned down by so many places. ... It might be because of my qualifications or whatever, might have a criminal record or might be this, might be that but, God knows, how I see it: bloody epilepsy! Who wants to employ anybody with epilepsy?"  **Article 456** "The spiritual burden is great to me because I have had to give up a lot of aspirations and leave my fate to chance. In fact, I want to work, which can make me feel better – but no one would like to employ me as I have the disease. Look – my hand is very strong, but it is difficult to find a job." "I mainly wonder if my daughter’s sense of self-respect will be hurt and if some insensible person will laugh and tease her, so that she thinks she is worse than other people. Secondly, I wonder whether others will look down on me and say we bore and brought up an idiot child."  **Article 460** "Viewed it as a 'mental illness' associated with 'shame' and 'ugliness' and that people with epilepsy were seen as 'dangerous' or 'strange.'" "They looked at me as a weirdo." "In interviews for work, I don’t tell. I know that people have pre-conceived notions. After a few months, this doesn’t scare them as much."  **Article 494** "If the church is supposed to be the backbone of our community, then why can’t I go to them for this problem [referring to epilepsy] without them thinking that I’m on drugs or something?"  **Article 501** "And this stigma, there are still too many silly myths that are connected not necessarily in people’s conscious awareness but in the background of their minds, this idea of witchcraft etc., is still there floating around in the atmosphere, in the air you know." "Well, no one has sat me down and said, 'This is what epilepsy is'… It still seems very mysterious. It still has a stigma attached. There are still people out there who think it’s the mark of the devil, and so on." "Well, why did it begin? I’ve had people tell me I’ve got the devil in me, and I think some people really believe that, and it’s absolutely shocking as far as I’m concerned. It’s preposterous. It’s ridiculous. It’s just a chemical imbalance in my brain."  **Article 502** "Other people, sometimes you sense, they just keep away because they don’t want to get it [epilepsy]. They think they might catch it. Sometimes you shake hands with other women if you’re greeting them, but they don’t want to put their hand forward, they don’t want to shake hands, so I just think 'leave it.' I mean, I know it’s not an infectious condition." |
| Acceptance of epilepsy | **Article 062** "When they told me, I tried to be calm. Some people get nervous, I did not. (…) I would like not to have it, see? But, as I told you, I have to face it."  **Article 063** "I suppose I don’t want to accept that it is a part of me. I can’t… I don’t feel ready to do that yet." "No, it’s just, it’s something that I have, not something that I am."  **Article 115 Supplement** "I believe I have then reached a point where […] maybe I can even say: Good, I can live with this." "I am positive […] that someday I can even have a fit while passing by. What a beautiful prospect." "I can accept it now. I am not pleased that it is there, but my approach is now a different one."  **Article 120** "In most cases, I think it starts with you, the person with epilepsy. You should accept your condition and educate people around you."  **Article 136** "I suffer from epilepsy, and I kind of very much felt that it’s part of who I am, but it’s not who I am…" "That it didn’t make me who I was. I was much bigger than it—and if I could accept it, it would be fine."  **Article 140** "Nowadays, I can live with it, but I don’t accept it. It’s a very sad illness, I don’t accept it. I’ve had a seizure in the street and nobody helped me, people run away, scared. I don’t accept being chosen for this kind of thing."  **Article 146** "I felt that I wanted to discuss my story... When I said it out loud, it was a way of admitting how bad I have been over the years." "I am a bit more comfortable now from just talking about it with a class full of other people who are going through similar types of experience." "To give advice to whoever is still in the confused world of ‘Oh why me, I don’t deserve epilepsy.’" "Well, actually, do you know what? It’s what a lot of other people deal with, you know."  **Article 151** "I prefer 'They’re epileptic.' Epilepsy is a part of me, no big deal. It’s nothing to be ashamed of."  **Article 158** "I get on with it. It’s not use sitting down and crying. It’s like you, if you look at it, could be somebody that’s worse off than what I am, so it’s like we’ve learned to live with it. It’s not nice."  **Article 169** "But in the end, what I think, it’s [epilepsy] always going to be there. Regardless of how long it’s been, it’s [seizure] always going to be a possibility."  **Article 188** "If you, as with in my case, accept you are epileptic and nothing can be done about it, accept it and get on with life is what I done … I decided, well, I am epileptic, so what. That doesn’t mean I am different from anybody else. Just means I have got epilepsy." "I sometimes talk about it to friends and they kind of say you know, your epilepsy is not a big deal, we never notice it, and if we do it’s no big deal. If anybody else was to notice it they probably wouldn’t think anything of it, and you shouldn’t let it constrict you in terms of where you want to go in the future, because things are always achievable… and then you kind of put it into perspective."  **Article 221** "I am what I am. If people don’t like me, I don’t care whether they do."  **Article 238** "I also try to accept that my disease will always be with me, and I trust in God that someday I may recover."  **Article 253** "I was so psychologically destabilised by the grief of losing who I was (and) then coming to grips with the degenerating condition."  **Article 262** "It’s learning to live with epilepsy, the initial shock, finding a voice, positive steps about epilepsy." "Because I know a bit more and before [the course] it was actually having acceptance of epilepsy [that was a problem]."  **Article 276** "I have had epilepsy for 16 years and yet still find it hard and difficult to accept. It has an effect on my life, especially work and social life, which is why sometimes I get angry and find it difficult to accept."  **Article 320** "Instead of trying to make everything seem like it was the same… I finally accepted that things are different."  **Article 339** "I learned to accept my disease, and I learned how to feel about myself—to not hate myself … 'Ah ha! I’m not different. I’m an ordinary person.' In other words, anybody can have this disease."  **Article 351** "I’m confident now… she made me into someone with the confidence to talk about it. It’s really helped me talking about it… [T]he shame of myself as an epileptic patient has also drastically reduced."  **Article 358** "Cos I feel like it’s a fuss cos I’m there. It’s only a fit I’ve had, cos I’ve got used to it now and I’m thinking they don’t need to fuss round me and putting all these things on me when I know I’m OK… don’t need it."  **Article 384** "Well, the seizures are, I know exactly how they work now that time has passed, I know exactly now."  **Article 441** "Oh, I was so relieved it was epilepsy. Everyone thought I was crazy! I knew I wasn’t crazy, and now I could prove that to everyone else. And I’m so glad I have the medicine to treat it … I mean it’s treatable, not like being insane."  **Article 453** "... a little ashamed of it." "I felt embarrassed about being diagnosed with it ..." "Having had three spells where I’ve come off it and had problems, erm ... you just accept it, it’s part of life, you know. But I suppose I’m fortunate, and it is pretty well controlled on medication ..."  **Article 456** "PWE see it as a disgrace and are reluctant to tell others about this condition." "Yes, I did [talk about it], there is nothing to hide. My disease is not a social disease, so I’m not afraid of it."  **Article 532** "I think because all these years I have had to cope with a cardiac problem, it’s just something more that I have taken on board, you know."  **Article 540** "I think because all these years I have had to cope with a cardiac problem, it’s just something more that I have taken on board, you know."  **Article 586** "It ruins your future, it completely ruins your future... I just wish I didn’t have it, you just feel angry, you think, 'Why should it be me?' you know, why do you have to have these things and can’t control them fully?" |
| Fear of seizures - seizure related worry | **Article 008** "I got some fears sometimes that something can happen and then I’m not close to any of the people that I know."  **Article 033** "If the doctor ever said to me that I need to come off it now, then I would be terrified that it happened again… if you had to come off it, I would be just really frightened."  **Article 063** "I, well, I just dread them, I fear them, I’m afraid I do fear them. I am terrified. It’s the experience itself because it’s like some pernicious alien force taking over your body and you have no control over it."  **Article 074** "I’m afraid to be on my own at night, I don’t go out on my own. And it’s really, it’s something that is hard to live with and cope with." "You’re walking home at night, am I going to have a seizure now, will someone find me, is it going to be the right person that finds me, or is it going to be a bad person that finds me." "There was always this… worrying about if and when the next one would happen, what I would do." "I would look to see if there were people behind me, because I would think, 'what happens if something happens to me in the middle of here?'" "There are things that do worry me or get me anxious. Money, I worry about my health a bit too, […] but epilepsy the most gets me worried." "It’s just like you never know when it’s going to happen, and that sucks."  **Article 084** "I think ‘what if I suddenly have a seizure?’ I like to be in control."  **Article 115 Supplement** "I am not afraid […] of epilepsy itself, not even of these seizures. I know that these seizures won’t hurt, […] [i]t is just the fear and the worry: When will it happen? Where will it happen? Who will be with me? Yes, these are the three things that make epilepsy difficult for me." "Now, it could happen: 'I sit, [breathe] and swallow, listen within, it’s nothing, nothing is going to happen.' It is just the fear, and then these words echo in my mind." "These dark clouds (the thought 'What, if now?') that have burdened me have thinned out more and more. […] And then there was one day where I was reflecting in the evening ‘I would not know. Was there anything today?’ And one day would sometimes even become two days." "Earlier, I would have been worried a lot and panicked or something like that, but not anymore."  **Article 137** "Very often I’m looking in the corner and thinking, that feeling I’ve got, is this the beginnings of an event?"  **Article 158** "I think he worries about having seizures. He thinks that nearly anything can set them off. He thinks—he used to say—'oh, don’t want to work on the computer too long, it’ll set off a seizure,' and we had to explain it’s doubtful, so yeah, I think he worries about having them."  **Article 169** "I hate that sort of anxious feeling, of like, 'Am I going to have a fit?' I struggle to get out of it… If I have all that anxiety, it doesn’t help." "It got to June, and I had to say to my rowing people, 'Look I can’t go on … I am almost endangering you by me being so worried all the time.'" |
| Ability to disclose diagnosis to others | **Article 136** "I think that is one of the reasons why I didn’t (tell). I lived in denial for so long because I didn’t know actually how to explain properly to people that I have epilepsy." "Maybe talk to close friends, get used to saying the word out loud, get used to being comfortable with it… And then once you get used to talking to close friends about it and dealing with their reaction… Then maybe you can get ready to tell other people."  **Article 144** "I need to know how best to share with others [family/friends/workplace] the implications of having epilepsy."  **Article 146** "I have never talked about epilepsy to anybody other than the doctor. I’ve never really had a general conversation about it... I suppose I am a bit more comfortable with it now through just talking with a class full of other people who are going through similar types of experiences."  **Article 205** "Some years ago, I was going to marry, but something happened and I decided not to marry. It was better for me. I didn’t know that spouses can cancel the marriage because of this disease. One time, I heard from my friends that my illness can cause dissolution of the marriage. At that time, I loved a boy, and he was in love with me, but I decided to break off the relationship without saying the reason and never marry anyone."  **Article 221** "In the epilepsy chat room, you can talk about more or less everything that goes on as on a day-to-day basis, your medication and everything…" "I didn’t make it public, but I didn’t entirely hide it... I would tell them, but if they didn’t ask, I wouldn’t tell them... I’m only recently trying to be more open about my epilepsy online because, for years, I wasn’t very public about it... It’s a new thing for me to be able to discuss it and, for so long, I couldn’t."  **Article 262** "Oh, the confidence to talk, yeah. Because it has given me more, more confidence, because I know a little bit more… and it was meeting other people as well and being able to talk about it."  **Article 286** "I’m still not 100% comfortable telling somebody. I don’t know what they’ll feel about me."  **Article 320** "It was easier to kind of fade out for a while so I wouldn’t have to tell anybody." "Getting it out there in the open, just talking to [my husband] and acknowledging that things are different, that helped ease tension."  **Article 332** "The fact is, that, over the years, living with epilepsy has shaped my thoughts to the point that even today, if I am asked, I do not admit to having epilepsy, because I feel ashamed."  **Article 397** "There’s no point in keeping it a secret, it is better to tell… That gives you more respect, and they can better understand how damned awful things can be sometimes…"  **Article 456** "PWE see it as a disgrace and are reluctant to tell others about this condition."  **Article 460** "Inside I know. I feel that something is eating away at me because I am keeping a secret."  **Article 586** "At first, they didn’t really know what it was, but I gave them leaflets to read and I said, 'Read these, and they’ll help you out, if ever I’m out and I do have a fit, you know, you know what to do.'" "Recently I moved in with my boyfriend, and obviously I had to tell him, and that’s the one thing that did scare me, when you have a close relationship with someone... He just sat there, and went, 'So?' He was very good about it, unbelievably good. You know, when they say, 'Oh, it doesn’t bother me,' you can’t believe that they’ve said it, and I find that I’m always asking him, 'Does it bother you?' because I can’t believe that it doesn’t, ’cos so many people are bothered by it." |
| Fear of seizure related injury | **Article 062** "The other day I was cooking, and I had a hot pan boiling there. I was about to make milanesas, and I had an attack. Luckily, I fell back, otherwise… The pan didn’t fall on me, otherwise I would have been all burned."  **Article 074** "I could have a seizure walking down the stairs today, and there someone could have left their cup of coffee on the stairs and I could trip over it whilst blacked out and break my neck."  **Article 079** "I didn’t… I feared to work because I thought that I would fall down on wood or other things."  **Article 369** "It scares me because I think something might go wrong. Like I might hit my head or something. Or I might, I don’t know, choke or something, like in the wrong way. But I’ve always got someone with me when I’ve had one, so I’ve been okay." "A vegetable basically. And I wasn’t, wasn’t afraid of dying really, I was afraid of that because I always thought if I have a seizure and I die I don’t know about it, that’s it, but if I’m in a wheelchair or I don’t want my life ruined by it."  **Article 397** "My worst nightmare is that I will have a seizure standing on the platform waiting for a train and then just walk on straight ahead…"  **Article 429** "I constantly worry for my daughter’s welfare, especially on roads or if she fell and banged her head. Unsure what medicines to get to cure condition. Always has a feeling that she will die suddenly."  **Article 440** "Epilepsy is frightening... the accident is easy to occur because you do not know when it [seizure] will attack you."  **Article 449** "This disease has caused me a lot of problems. I can’t walk around freely. I can’t cook because of the fire. I am not free to do things since at any time I could have an attack. I am not even free in my own home. I can’t go to draw water alone. I can’t bathe alone. All diseases hurt, but this one has really affected my heart." |
| Perception of being in control | **Article 074** "I feel like my ability to understand it and manage it, and not feel I have to do anything about it, actually enables me to feel like I’m more in control of it."  **Article 136** "It’s (epilepsy) a part of you, you’re not a part of it!" "If I was letting it control me, I wouldn’t have been swimming, I wouldn’t have done anything like that. So trying not to let it rule everything." "That it didn’t make me who I was, I was much bigger than it—and if I could accept it, it would be fine."  **Article 137** "I think I have total control now I have this Tegrotol pills."  **Article 158** "I know I can keep more control of them now."  **Article 169** "Sometimes I think mentally you can control it anyway."  **Article 285** "It doesn’t matter how carefully I take my medication or that."  **Article 328** "Yeah, I’m sort of a person who likes to be in charge and have a routine and things like that… if a change is going to happen I have to plan it."  **Article 460** "I thought epilepsy was going to complicate my life over the years. I felt anxiety every day and I was afraid to lose control." "The only benefit of the illness is a better coping with life; I definitely control my disease." |
| Sense of embarrassment | **Article 074** "I’d feel really embarrassed about it, intensely. I think they’d think it was something to do with drugs or something else."  **Article 136** "It’s not something you should hide, it’s not something you can be embarrassed about or should be embarrassed about. It’s nothing shameful, and it’s up to them how they accept it."  **Article 204** "Oftentimes, I see my family refusing to talk about my illness in front of others, and they’re embarrassed when I have a seizure and somebody from relatives is in our home."  **Article 238** "I do not admit having epilepsy because I feel ashamed."  **Article 286** "The shame on me, it was too much [on her], and the name on the family…. Another family doesn’t want anything to do with your family." "It’s an embarrassing feeling. Especially when you see children, and my wife told me at one time, that children, they were crying. So it wasn’t a good feeling, this. I didn’t have the confidence, without my wife."  **Article 320** "It was easier to kind of fade out for a while so I wouldn’t have to tell anybody."  **Article 332** "Others which could bring me shame and disgrace. This could make me feel ashamed every time I see them." "The fact is, that, over the years, living with epilepsy has shaped my thoughts to the point that even today, if I am asked, I do not admit to having epilepsy, because I feel ashamed."  **Article 453** "I felt embarrassed about being diagnosed with it." |
| Sense of normality | **Article 008** "Fifty percent of the treatment in my disease is that you should feel that you’re normal." "That this disease isn’t going to prevent me from going out, living my normal life, and doing things I want to do."  **Article 137** "Make sure you can live your life normal, or as normal as you can really."  **Article 207** "Obviously the seizure happened in work you see, that is a big factor. … I wanted to see if I could get a letter from [hospital] saying [name] is now on medication and he can live a normal life and he is fine to do this, he is fine to do that… and then work to say, right, okay, we will tick our box. Carry on like you were before." "I got on the gabapentin, and even though it didn’t stop my fits, it changed my life drastically. I got some sort of what people would call normality because I had never had that before… it gives me longer to be me."  **Article 216** "[Seizure management] has given us a bit of family life back. I can now have holidays. I can have a relationship with my partner. His sibling can have time with me now … It’s been completely life-changing. We are now a functioning family as opposed to a family in a state of constant medical crisis."  **Article 384** "I have tried to live a normal life. There shouldn’t be any obstacles because I have epilepsy. I have always thought that I shall live life, there’s no problem."  **Article 441** "It’s so frustrating to see your friends living all normally, and you can’t."  **Article 453** "I just want it out of the way, so hopefully I don’t have to say, put down on forms you’ve got epilepsy ... Yeah, so I’m normal."  **Article 586** "Cos what I try doing is forget about it and pretend it’s not there, even though it is, I try to get on with life as well as possible." "I try to just forget about it. I go out with my friends a lot, and just feel like a normal person. I don’t feel like a different person to everybody else, ’cos I know I’m normal." |
| Sense of isolation | **Article 136** "Feelings of anxiety, feelings of isolation."  **Article 146** "To see how they [other course members] deal with it makes it more easy to live with your illness because you think, 'I am not alone.'"  **Article 286** "I couldn’t go swimming; I couldn’t play computer games, bike riding. I couldn’t go too far because my parents were worried about where I was going and if I had a fit."  **Article 294** "Well sometimes you feel alone, you know. You’re the only one that’s doing it… she doesn’t want to be a burden on anybody, and, you know, she and I have an agreement that I’m it."  **Article 310** "I used to have three jobs at that time, and as soon as epilepsy hit me I gave them all up — thought no, I can’t work, I am terrified in case I have a fit… because I mean when I fell on the concrete I split all my head open and I had to have stitches inside and outside, I mean, I could have died on that floor... I gave up all my three jobs, I had to give up my bike — and yes it was really… I don’t know really how I did cope really, because I felt so isolated."  **Article 397** "I can’t visit my friends anymore… and if I do, I always have to have someone waiting for me, so I can never relax… in the end I will end up going nowhere in case of having a seizure... the risk is that one becomes… well… kind of isolated."  **Article 402** "I loved my wife very much, but she decided to abandon me because of my epilepsy after living together for about 40 years. Her relatives contributed a lot to our divorce. Now I am alone and I don’t have anyone to take care of me as she used to." |
| Fear of SUDEP | **Article 280** "Yeah it’s not a nice thing, especially when it’s uncontrollable... when it goes on for a minute, a minute and a half, you honestly think you’re gonna die."  **Article 285** "I didn’t look up any of the details cos there’s some things that I just think I don’t want to know more about it because I will just be terrified, and it’s not going to help me, it’ll just make me worse… SUDEP would have been something that I would have just went and looked at, and like, say it was one in a hundred, I’d have went: ‘Oh my God, I will be the one in a hundred... I’m gonna die in my sleep.’" "I felt gutted, like… cos I just found out… that there was a new concept of dying… it was like ‘mind blown.’" "I’m kinda a believer in fate type of thing, so if something’s gonna happen, it’s gonna happen anyway, but… you can’t really live your life in fear of what might happen."  **Article 288** "I didn’t look up any of the details cos there’s some things that I just think I don’t want to know more about it because I will just be terrified, and it’s not going to help me, it’ll just make me worse… SUDEP would have been something that I would have just went and looked at, and like, say it was one in a hundred, I’d have went: ‘Oh my God, I will be the one in a hundred... I’m gonna die in my sleep.’"  **Article 351** "Having epilepsy; you’re going into a fit. You don’t know if you’re going to wake up or die."  **Article 358** "I was afraid I might die, because it could kill." "I don’t want to die. Anything could happen, you see this epilepsy can happen to you, anywhere you know. So happy to call 999 for me."  **Article 429** "I constantly worry for my daughter’s welfare, especially on roads or if she fell and banged her head. Unsure what medicines to get to cure condition. Always has a feeling that she will die suddenly." |
| Perception of lack of control | **Article 063** "What probably makes it worse is there’s nothing I can do to stop it."  **Article 136** "You’re so limited in so many ways; it’s so easy to let it take over your life."  **Article 185** "I wish they could ask me how I am coping with this illness… I am troubled by this illness… It is because when you are not well and being held by an illness… you’re always not happy because I’m always busy thinking of how I can get a plan that can free me."  **Article 397** "When you have had a seizure and wake up at the hospital, you don’t get it at first… that you have had a seizure and then… you think about… how did I look?… what people had gone by and seen me?… and who took care of the whole situation?"  **Article 449** "When I am in an attack, anyone who finds me could just take me somewhere. Sell my organs. Assault me. I am not aware and would not be able to defend myself. It is worse in the city, I think."  **Article 453** "Frightened whether, if it was going to happen again was a big thing to me because losing control of what you’re doing... what your body’s actually doing, you’re not aware of it... it’s quite frightening so..." |
| Self confidence | **Article 136** "I joined Epilepsy Ireland and they explained to me that, okay, it’s a support group that comes in and you don’t have to say anything. Just knowing that there are other people like me, that gave me the confidence and the courage to actually say, okay, let’s do something about this."  **Article 262** "I came away from this feeling more confident and proud."  **Article 310** "And the other thing was getting my driving licence back. That is a confidence thing. That was a huge thing to me… It was the confidence side, yes. I felt almost whole again, you know. I had got my driving licence back. I was as I was before, you know."  **Article 332** "We lose self-confidence to some extent. For instance, I was in charge of planning and had to explain a performance report from the mining industry executives to the board of directors at the company. The entire time I was afraid that a problem could occur during my presentation and expose my secret of having epilepsy. I had a suspicious feeling that some people at the office had noticed my nervousness and wondered why. I was protecting my secret and experiencing low self-confidence." *(60-year-old man)*  **Article 403** "The waiting list is crazy… I was housebound for a year and a half having dropouts and seizures and dropping confidence… in my GP waiting room… in front of people." |
| Sense of being defined by epilepsy | **Article 074** "I will not be defined by these drugs and I will not be defined by this medical condition."  **Article 151** "Epilepsy is a condition you have, but one that doesn’t define you as a person. This expression helps separate the two." "I prefer ‘person has epilepsy’ because the person has more than epilepsy, they have talent, they have a soul, they have a dog… it’s just one part of them and this term reflects that." "I haven’t had a seizure for several years, so I do not like to class myself as being ‘epileptic’ because it is controlled."  **Article 188** "If you always think about being epileptic, you think poorly of yourself. Your quality of life will be poor, no matter how much money you have got."  **Article 221** "I am what I am. If people don’t like me, I don’t care whether they do."  **Article 397** "Ever since my childhood, I have thought that I must be the one ruling things in life, not the epilepsy. This has been my challenge all along…" |
| Self efficacy (regarding uncertainty of seizures) | **Article 074** "You don’t know what’s going to happen, so there’s no point in worrying about it."  **Article 216** "It makes me scared to go out. It is very hard. I don’t know when I am going to have a seizure – they come out of the blue. I don’t even want to go and get a cup of tea."  **Article 451** "I will have a fit if I’m still taking my tablet but, if I miss a tablet, I’m still going to have a fit. If I miss the tablet, I might not have a fit, you don’t know. It’s one of these things, it’ll just come up from nowhere."  **Article 453** "Why, one minute you’re alright and then the next minute you’re bad… They just came on like that (clicks fingers). Quick." |
| Self-resilience or self-efficacy (NOS) | **Article 069** "During our meetings, the advice from colleagues gives me hope that all is not lost and that I am not alone… I am able to live with people."  **Article 115 Supplement** "So, I listen to myself a little more and decide what is good for me and what isn’t, and I think that has at least gotten me where I am today."  **Article 137** "I believe in getting up and getting out and doing what you can."  **Article 158** "No, you just, with epilepsy, yeah it’s hard at first, you get stable, you have your ups and downs and get on with it." |
| Fear of medication side effects | **Article 074** "I’m not taking this. And [the doctor said], ‘Oh don’t worry, they just list all the side effects, just take it,’ you know. […] But by the time I was so alert for side effects that I was a bit of a hypochondriac."  **Article 369** "I found more about [er] sort of side effects of tablets, which made me more [er] paranoid about the side effects, which brought the side effects on more."  **Article 397** "A fellow patient said that some epilepsy medication can cause Alzheimer’s or cancer. I asked my doctor, but he said not to worry… but you know… yet I worry…" |
| Low self esteem | **Article 310** "I think the other thing with the seizures, it’s the side effect is you don’t feel so confident within yourself, it affects your perception of yourself. I was very negative, I got very depressed, well when I say very depressed, I don’t mean so I became a depressive, but I didn’t know when I was going to have a fit, so I was always thinking about that, it was always on my mind."  **Article 397** "Well then I went so completely, yes really completely under… everything just went down… my self-esteem, you know… it was so depressing… building up hope gradually again… and every time it does not happen… If this goes on, I will not feel very well…, in fact, I will feel really terribly rotten."  **Article 453** "In this day and age, it isn’t really, like the medication I take doesn’t make it a problem, I’m able to drive and what have you, but it’s in my mind that, I don’t know, I’m less of a person than what I am." |
| Impact of epilepsy on sense-of self | **Article 063** "It’s very, is very demoralizing because you don’t... you don’t have this control over your body, you know you don’t know, your brain, your epilepsy is controlling you up to a point, yeah." "…We all, I suppose, like to be in control of all things, never mind control of our own bodies, and the nature of epilepsy is that you lose voluntary control, and that’s probably the worst thing about it… If you can’t control your own bodily movements, then what hope have you got of controlling anything else in your life if you can’t even control your own bodily movements." "I think it was my perception of my self-image. I just had to adjust to the fact that I was, I was carrying something else, that there was another part of my personality."  **Article 221** "I don’t wear a little badge saying I’m epileptic... I put it on my Facebook to make it clear to people who support it. So it doesn’t worry me now. Certainly, with the fact that I’m working for myself it doesn’t worry me."  **Article 253** "I was so psychologically destabilized by the grief of losing who I was (and) then coming to grips with the degenerating condition." |
| Sense of not being defined by epilepsy | **Article 188** "[Quality of life] is the same as anybody else’s [quality of life], because, in fact, I don’t even think about it [epilepsy] anymore."  **Article 230** "I actually think it is all right [the fact that there is no routine visit anymore]. Because, I really don’t feel that I suffer from epilepsy. I actually don’t feel like that. I feel well in my everyday life, so I kind of forget that I have epilepsy."  **Article 453** "I just want it out of the way, so hopefully I don’t have to say, put down on forms you’ve got epilepsy... Yeah, so I’m normal." |
| Positive sense of self | **Article 310** "I think the other thing with the seizures, it’s the side effect is you don’t feel so confident within yourself, it affects your perception of yourself. I was very negative, I got very depressed, well when I say very depressed, I don’t mean so I became a depressive, but I didn’t know when I was going to have a fit, so I was always thinking about that, it was always on my mind."  **Article 339** "I learned to accept my disease, and I learned how to feel about myself—to not hate myself… 'Ah ha! I’m not different. I’m an ordinary person.' In other words, anybody can have this disease." |
| Absence of seizure related worry | **Article 169** "I actually just really enjoy doing it. I don’t really think about my seizures per se. I think occasionally it pops into my mind, but I get so invested into my exercise and the effect it’s having on me and how much I’m enjoying it, that it doesn’t really come into the equation." |
| Acceptance of SUDEP risk | **Article 288** "It’s not something that I have to deal with every day... it’s just, it’s not something that concerns me on a daily basis... I don’t take my medication because of SUDEP... yeah, I just do it to get on with my day." "There’s not a lot you can do. If you’re gonna die, you’re gonna die. I’m kinda a believer in fate type of thing, so if something’s gonna happen, it’s gonna happen anyway, but... you can’t really live your life in fear of what might happen." |
| Fear of seizure whilst swimming | **Article 074** "Generally, there are things that I feel reticent about. So I don’t always feel comfortable in water." |
| Feeling of vulnerability | **Article 328** "I still feel vulnerable even on these pills after what happened… I feel very, very vulnerable. I just wanna… it’s weird." |
| Irritability | **Article 429** "Child’s character has changed. He is very irrational and hot-tempered at times, often hitting out at us and then apologizing." |
| Negative sense of self | **Article 074** "There was a time in my life that I was actually intelligent […] I don’t know where I am now, I’m just stupid now." |
| Anger or frustration with seizures | **Article 115 Supplement** "One time, after I had already had 2, 3, I was fed up, let’s say, and said: I don’t want this anymore now." |
| Sense of being helpless | **Article 238** "I got tired, I surrendered, I feel damaged by the disease, it has affected my nervous system and I have lost patience." |

F. SOCIAL AND ROLE FUNCTIONING OUTCOMES

| OUTCOME CODE | VERBATIM PARTICIPANT TEXT |
| --- | --- |
| Work status | **Article 035** "I couldn’t go to work, there is no bus to my job, what am I supposed to do …"  **Article 062** "That I can get a job, and that there are thousands of jobs I can do off the books. They recommended me to look for an illegal job." "They offer job positions I can’t take because I can’t use the shovel and I can’t make any physical efforts. I get angry for that." "They were going to help me to have a job, see. And I couldn’t, because they found out I had this illness." "They said that there was no problem, that I could stay because they were satisfied with my work. So I stayed. But after that I started to have problems with my supervisor."  **Article 069** "Not serious because I was able to continue with my work after the seizure, but I was sacked and I have become jobless since then." "While my educational background is good, ﬁnding a job is difﬁcult; I have tried many times without success, employers do not employ me because of my disease. As I may experience seizures on the job, I disclose my condition to potential employers, but being truthful has affected me negatively." "My supervisor inquired about what was wrong with me, but I did not give him details about my condition. He would have sacked me immediately if he had known my condition." "When I became conscious, my supervisor said I was epileptic. Although I told them I did not know about it, the authorities maintained otherwise and ﬁred me. I did not disclose my condition initially for fear of failing to get the job. Before I was employed at the institution, I had moved from one place to another in search of a job, but to no avail. Wherever I went, I was denied one because of the disease. Now, I am without a job."  **Article 074** "Having to resign from work as a bus driver." "It just makes me really angry […] this is taking my career away, and living away."  **Article 079** "I didn’t…. I feared to work because I thought that I would fall down on wood or other things." "I worked at those times but now I am tired.… I am unable to work. I am tired."  **Article 120** "Get yourself treated, maintain yourself so that you can keep your work."  **Article 188** "I was working as well—which probably helped quite a lot. I was a manager, life was going straight."  **Article 204** "I was a construction worker. Few times at work, I had seizure and for this I was kicked out by my employer. I often get a negative answer for my illness wherever I refer to for employment. I decided to stay at home. Actually, I’ve become a burden on my family."  **Article 221** "I don’t wear a little badge saying I’m epileptic...I put it on my Facebook to make it clear to people who support it. So it doesn’t worry me now. Certainly, with the fact that I’m working for myself it doesn’t worry me."  **Article 238** "Epilepsy is a major barrier to finding and getting a job for most of us. As the saying goes, “you can’t always get what you want.” We lose many things because of unemployment. We are dependent on our families, and cannot become independent. This is a big problem."  **Article 253** "Once they know you have epilepsy and still have fits, they are frightened to employ you on manual jobs."  **Article 286** "There’s certain times when I concealed it for a time…. For example, going for a job… I would conceal it up until the point where they make that decision. Because although…you’ve got the disability discrimination act…"  **Article 310** "The job went and everything else and I ended up for the first time in my life on the Dole [Unemployment Benefit]… Gutted, absolutely gutted, couldn’t believe it, still hurts now you know… really does. You know being out of work, it really, really bothers me." "I mean my main aim in life was working as a [work role], but it [QOL] has certainly changed because they finished me because I couldn’t get control of my fits … I was absolutely devastated… it has took me years to get over it." "And then getting back to normal, going back to work." "I left the job I was in when I had the first seizure, and it was hard to get a job you know, because you have got to put it on the form, things like that—and I felt because it took me a long time to get a job, and that people sort of take that into account and go, oh, we don’t really want you working for us if you are going to be you know sort of…"  **Article 317** "And the attacks, the visits to the doctors, the meds, everything. I became so depressed that I didn’t want to go to high school anymore. At jobs it’s the same…what lowers my mood is to try many times, and again and again, and sooner or later you get ﬁred from all. For me, it’s enough!"  **Article 320** "I went from part-time down to really part-time."  **Article 332** "Employment is a major problem for most of us. We are unemployable in many places because of epilepsy and government jobs are not an option with this diagnosis." "I did not want to have a military draft exemption because I knew it would raise questions and limit me for future employment. I understand that employers look for healthy individuals and are disinterested in sick people with medical exemption."  **Article 384** "These choices you make, like having children, what work to do, how much to work, there’s so much, everything becomes so tough. You can’t just do what everyone else does, nah, but now I want to."  **Article 429** "I can go to work everyday. If I feel tired then I will take a couple of days off from work and return when I feel better." "Also epilepsy can be more difficult for women in terms of getting married, going to school, or finding a job." "Epilepsy is more difficult for men because men are the workforce of the family. If a man does not work, it would be a burden."  **Article 440** "Epilepsy is heavy on the economy, [wife] doesn’t work and I laid off, all my family rely on the subsidy. The drug takes 100 yuans per month, and if unfortunately, we should go to the hospital the subsidy will be used up."  **Article 441** "Because I can’t drive it is so hard for me to get to work—it’s been a huge issue just to get to my job or meet other responsibilities. If I can’t work I can’t pay my rent."  **Article 449** "My marriage has changed. I don’t work now and cannot bring money like before. And because of my epilepsy, I can’t find work… because my condition is known, no one will hire me."  **Article 455** "With the epilepsy, I’ve been turned down by so many places. ... It might be because of my qualifications or whatever, might have a criminal record or might be this, might be that but, God knows, how I see it: bloody epilepsy! Who wants to employ anybody with epilepsy?"  **Article 456** "The spiritual burden is great to me because I have had to give up a lot of aspirations and leave my fate to chance. In fact, I want to work, which can make me feel better – but no-one would like to employ me as I have the disease. Look – my hand is very strong, but it is difficult to find a job."  **Article 460** "If there are seizures, there isn’t work; if there aren’t seizures, there is work." "I’m not worried, I’m in the last year of high school, I work in a coffee shop, and my love life is great." "The few things that remind me and annoy me about this situation are the alcohol and driving license restrictions and the obstacles to serving in the army like anyone else." "I can’t combine studies and work because of my daily routine."  **Article 531** "Yeah, the ﬁrst two years were just absolute, sheer hell, with losing your job, friends stay away... people seem to disappear." "Before I became epileptic, I was the type of person who’d take care of myself... how do I take care of myself when I can’t get a job?" |
| Driving status | **Article 035** "… he was like, ‘leave your car in the car park’… I had kids to collect from school … they literally rip the end out of your world when somebody tells you that …"  **Article 074** "Although I am able to drive now, I wouldn’t want to drive early in the morning if I knew I was a bit tired or had a drink the night before,"  **Article 103** "If I have a seizure and worried about losing my license and house."  **Article 115 supplement** "It is such a task to organise driving services since I cannot drive." "To have it under control to the extent, so that I can find my way back to being with my workplace colleagues, where I then can drive to again by car." "At the same time, nobody sa[id]: 'You do not need to attend the meeting in the neighbouring city'. […] That means I would like my permission back to drive a car." "Considering, I would actually have a fit while driving a car: I don’t have to talk anyway."  **Article 136** "It does kind of dominate your life though, ’cos you can’t drive."  **Article 137** "If we are going out with friends then they have to be the drivers and I have to be passenger."  **Article 140** "I have many dreams for my life, but when I remember this damned epilepsy, it’s all over. And I know that if I try to get my driver’s license I will have a seizure."  **Article 158** "There could be one day where I get really frustrated because of it [epilepsy], and you know, because I can’t do the things that I want to do, like I put up this wall about driving but lately I’ve been saying I want to get, I really do want to get stable so I can drive."  **Article 169** "I hate that I can’t just drive and just get home. The same from swimming…. If I could get to swimming events on my own …. But I don’t, I have to rely on everyone."  **Article 188** "Apart from the definite effects it [epilepsy] has on memory, it really hasn’t made a fantastic difference now that years no driving is up."  **Article 207** "Kind of take most side effects if it means that I can get my licence back and become seizure free and stop that worry… about fitting and, the side effects of tiredness and mood swings and irritability pale into insignificance compared to worrying about making an idiot of yourself in the town centre, by collapsing on the floor, or worrying about your daughters hurting themselves or not being able to drive."  **Article 253** "Loss of jobs, licences, control during fits and friends were pertinent and distressing."  **Article 310** "I was getting up to 11 months [seizure-free] and then I had another one. That was so depressing because I couldn’t drive, that is like, if you can’t drive, well to me that was my independence I couldn’t go nowhere, that is the way I felt you know." "And the other thing was getting my driving licence back that is a confidence thing that, that was a huge thing to me … It was the confidence side yes, I felt almost whole again you know I had got my driving licence back I was as I was before you know."  **Article 332** "Another problem we have is not having a driving permit. Being diagnosed with epilepsy means no driver’s license at all (25-year-old man)."  **Article 384** "Cars and motorbikes you can’t. I can get really angry about that. Just knowing that you can’t ﬂy airplanes, just knowing that you can’t … no, I think it really hinders me terribly."  **Article 397** "When I am out driving… and I drive a lot… I always look for a parking space in front of me… in case a seizure would come… I feel it in advance… I want to be prepared, you know."  **Article 441** "Because I can’t drive it is so hard for me to get to work—it’s been a huge issue just to get to my job or meet other responsibilities. If I can’t work I can’t pay my rent." "When I was ﬁrst diagnosed I had no way to get to work, and that made me so uptight and worried. If I didn’t get to work I couldn’t pay my bills, and my whole family would suffer. So I just drove anyway because I had to so that we could live normally."  **Article 453** "... now we were at the stage of getting him off them before he wants to learn to drive, ‘cos if he had to go back on them, then you’re losing your time."  **Article 460** "The few things that remind me and annoy me about this situation are the alcohol and driving license restrictions and the obstacles to serving in the army like anyone else."  **Article 532** "He didn’t even tell me that I shouldn’t drive or anything like that, so I just carried on (driving)." |
| Impact on relationships and friendships | **Article 062** "[One of my brothers] throws in my face that he has a girlfriend and I do not (…). He says ‘it’s because you don’t look for it’, and I tell him ‘you should be in my shoes, I exchange my illness for your health, and then we’ll see if you could stand a week with this. Not being able to do this or that’…"  **Article 069** "No lady would marry me with such a burden. I had a girlfriend who did not know I had epilepsy. When she became aware, she ended the relationship. I don’t think I would get married in the future." "The disease has become a padlock to my happiness and joy. If not, I would have been a married woman by now. I met an elder of a church, we dated and got married. We had a child, but when he became aware that I had the disease as a result of frequent seizures, he informed his mother who advised him to end the marriage and he did." "I have decided not to get married again to avoid troubles from any man who might marry me and divorce me later upon realizing that I have epilepsy."  **Article 137** "I think I am closer with (husband) because of it than I would be probably in an ordinary marriage kind of thing, you know."  **Article 188** "I told her [wife] about it, and we talked about it …. I said well it’s not going to go away and she has been the biggest help I have ever had really because she has stuck with me and said, well I can take it, so if there is one big, the biggest thing is my wife and the relationship, and the love we have for each other."  **Article 253** "Loss of jobs, licences, control during fits and friends were pertinent and distressing."  **Article 280** "It’s not a nice thing to happen and I would suspect that it could destroy some relationships."  **Article 286** "I had a couple of ﬁts… and I think he (her boyfriend) left me because of that… he beat me up, I was bleeding everywhere and… And all the time he would come to my ﬂat and then go there and telling everybody who knows me and him that ‘oh, she has bad disease, she falls down’."  **Article 294** "I get the sickness and in health thing, and it’s just, I don’t want any part of this … I wouldn’t shirk my responsibilities, because I take it very seriously, because I love her, but this is not what I signed up for. This is extraordinarily taxing on me."  **Article 320** "We’ve been together 40 years… real independent. But now…[my husband] hovers. I went from being wife…to child." "We knew that our marriage got rocky around [my diagnosis]… we ended up talking and realizing that it was hard for both of us… and we were able to stop resenting."  **Article 332** "I lost many friends because of my disease. They were afraid of continuing their friendship with me."  **Article 384** "When one of my daughter’s friends calls and asks if they can play, I say that I’ll tell her to call when she gets back and then I remember in the evening what I promised and it’s very … (makes faces) it also affects the friend who is waiting for her to call." "All of my friends disappeared, so yes, my circle of friends is very small as well." "It’s unbelievable sometimes. You’ve lain there shaking … and you’re exhausted afterwards. You’ve had so many. He’ll have to leave me soon because he’s completely exhausted. I had eight a day. But no … He’s still here."  **Article 402** "At school sometimes classmates are unwilling to be with me because they are afraid of witnessing a seizure."  **Article 441** "Sometimes I feel like a horrible partner ... with all the sleep I require and the fact that I can’t drive I don’t think I’m a very good wife to my husband ... I’m not the same wife I used to be."  **Article 449** "I had a seizure on the way [to clinic]. My children had to go back to the village for help and my family had to come and collect me in a scotch cart. My husband says he won’t divorce me as this problem started after we were already married, but one day he will tire of this and leave." "In 2001, my husband divorced me because of my seizures. My child was only 1 year old at that time and my husband took my child when he left." "My husband supports me. But since he took a second wife, he never lies with me. My husband’s family and the second wife are afraid she will get this condition from me. They think my condition shames the family. People tell my husband he should abandon me. Sometimes I drink." "My marriage has changed. I don’t work now and cannot bring money like before. And because of my epilepsy, I can’t find work … because my condition is known, no one will hire me."  **Article 460** "I have had an open and supportive relationship with my boyfriend for five years. We are now planning our wedding, and my life could not be better."  **Article 494** "Anyway, we went up to the counter and all of a sudden I went into one of my trance-like states, and he just went ballistic because he thought I was staring at the grocery clerk. He thought I was ﬂirting with him. I tried to explain why I had done that [the partial mini-seizure], but he [the ex-boyfriend] never believed it." "My husband left me many years ago. I’m basically all alone now. My parents are no longer living and I have one brother, but I can’t move to Texas with him."  **Article 586** "Recently I moved in with my boyfriend, and obviously I had to tell him, and that’s the one thing that did scare me, when you have a close relationship with someone... He just sat there, and went, ‘So?’ he was very good about it, unbelievably good. You know, when they say, ‘Oh, it doesn’t bother me,’ you can’t believe that they’ve said it, and I find that I’m always asking him, ‘Does it bother you?’ because I can’t believe that it doesn’t, ’cos so many people are bothered by it." |
| Impact on education and schooling | **Article 062** "Then, when I was in primary school, in second grade, they said I had to go to another school because I didn’t have high grades, because I was distracted…"  **Article 074** "Being away from school by having appointments, being admitted to hospital."  **Article 120** "Epilepsy started in 1984 when I was at boarding school, [school name removed]. I stopped going to school … [when I resumed] I did not go back to boarding school. I went to a local school."  **Article 136** "I’d a really bad experience where I was asked to leave the school because of the ﬁts I was having and that was just horrible."  **Article 238** "I left school because of being sick. I had a teacher who was very strict. During an examination, I had a seizure and he thought it was a lie. He took my hand and threw me out, disallowing me to finish the test session. On follow-up, even the school superintendent had a very bad behavior."  **Article 317** "And the attacks, the visits to the doctors, the meds, everything. I became so depressed that I didn’t want to go to high school anymore. At jobs it’s the same… what lowers my mood is to try many times, and again and again, and sooner or later you get ﬁred from all. For me, it’s enough!" "I went to the school, they asked me to go … because there were problems, sometimes she got sick and distracted the others, you know, because having someone ill in a schoolroom or anywhere always distracts the other people, and is a waste of time, to help her or lift her … always distracts … it was a problem in school so I took her out …"  **Article 332** "I ranked ﬁrst in my class until third grade, but in fourth grade I failed four subjects and later passed by re-examination. I fell behind in my lessons and since then I could not follow my lessons. I lost interest in learning and put off any efforts to excel. A sense of fear for having epilepsy contributed to my educational setback. At ﬁrst I did not feel well in a crowd and in the classroom and I used to leave the classroom to avoid mean stares. Unfortunately, I did not attend a university." "Many of them complained about going to school and being told that other children were frightened of their convulsion. Therefore, they could not return to school. They were told to go to a special school."  **Article 339** "That was sort of the tipping-point. I was on sick leave and in a pretty bad way prior to the rehabilitation, but afterwards I was able to go back to school and stuff."  **Article 384** "Studying at university, you are limited ’cos I have to get there and I get up early in the morning and I am tired so I have a seizure, ’cos that has happened—that I have been on the bus and, yes, I have had these seizures as I said before, uh, just stuff like that makes you reluctant to do certain things and, yes, but then I can’t do it, I can’t study right now." "It has had a pretty big inﬂuence, because, as we said about school, it has affected my grades, I haven’t had really bad grades but I know that if I could have studied more I’d have had higher grades and I’d have been able to study at university and had a better education and even had better, yes better, everything would have kind of been much easier."  **Article 402** "I was very bright in school and was selected to go for further studies in Nairobi but I couldn’t manage because of being epileptic."  **Article 429** "I do not let my child to attend at school because she can fall down suddenly." "People with epilepsy have no future and cannot go to school." "Also epilepsy can be more difficult for women in terms of getting married, going to school or finding a job."  **Article 441** "That medication makes me so sleepy ... that’s why I don’t take it if I have to stay up and write a paper or have an early class." "The day after a seizure I just feel so tired that I can’t even keep my responsibilities. I can’t even go to class. So I never plan anything the day after a seizure, and I have someone take notes for me in class since I know I can’t be there."  **Article 531** "I sleep sometimes (at school) ... (when I sleep) they send you to the principal’s oﬃce and send you home... probably like two times a week." "I am concerned about the long-term eﬀects of his medications and his abilities. His school grades have deteriorated over the years... He is either drugged out or exhausted from having seizures." |
| Ability to socialise | **Article 074** "I’ve deﬁnitely withdrawn socially almost completely […]" "I realised I’d prefer to have a seizure on a train than be locked at home with no friends and no social life."  **Article 115 supplement** "And then [after the fit] you don’t necessarily want to have too many people around you." "I really miss socialising with my colleagues at work."  **Article 158** "Monday and Thursday I go to activity centre. We cook for people, we were making jam yesterday. Wednesday I’m at Health Food Gardens down the road and Friday’s my shopping day. Tuesday the Social Worker, she’s trying to sort something out. Always seem to be on the go." "He goes out every other week on a Wednesday and sometimes he goes to the shops and sometimes he’ll see a friend of his, and he seems quite happy most of the time."  **Article 188** "But you do meet people at like the art group that take the place of work colleagues in a way, because you are talking about, you go to exhibitions and you go out for things, all to do with that subject … it’s a common interest, which is really good."  **Article 216** "It makes me scared to go out. It is very hard. I don’t know when I am going to have a seizure – they come out of the blue. I don’t even want to go and get a cup of tea."  **Article 276** "I have had epilepsy for 16 years and yet still ﬁnd it hard and difﬁcult to accept. It has an effect on my life especially work and social life which is why sometimes I get angry and ﬁnd it difﬁcult to accept."  **Article 286** "I couldn’t go swimming; I couldn’t play computer games, bike riding. I couldn’t go too far because my parents were worried about where I was going and if I had a ﬁt."  **Article 310** "I couldn’t go out in the evening and sit in a pub because I didn’t want to have a fit in front of everybody and, if I wet myself, embarrass myself… And that was depressing. Like I said to you, it made so many things go out the window, like … yes, social life, what social life?"  **Article 384** "It’s hard to make friends when you are going to explain, you have to say that you’ve got epilepsy and some people react in a way that you don’t want them to and some of them are like … I’m here for you and I’ll do anything for you … don’t pity me, that’s not what I want, just be a friend."  **Article 397** "I can’t visit my friends any more… and if I do, I always have to have someone waiting for me, so I can never relax…… in the end I will end up going nowhere in case of having a seizure... the risk is that one becomes… well… kind of isolated."  **Article 586** "I try to just forget about it, I go out with my friends a lot, and just feel like a normal person, I don’t feel like a different person to everybody else, ’cos I know I’m normal." |
| Ability to form companionship or get married | **Article 204** "Few times I have decided to get married, but as soon as they’re informed of my illness, my proposal of marriage is rejected. When I asked why? They told me you have a contagious disease or we cannot have children because of your illness."  **Article 205** "Some years ago, I was going to marry, but something happened and I decided not to marry. It was better for me. I didn’t know that spouses can cancel the marriage because of this disease. One time, I heard from my friends that my illness can cause dissolution of the marriage. At that time, I loved a boy, and he was in love with me, but I decided to break off the relationship without saying the reason and never marry anyone."  **Article 208** "I am not respected. I do not have the right to go to public places. I have been given a separate cup and plate. They do not want me to get married…"  **Article 238** "I am often thinking of marriage and the fact that I am suffering from epilepsy, whether I get married or not, whether my secret is exposed or not weighs heavy on my mind. At least three or four times I have attempted suicide. I am very tired of life. I often beat myself."  **Article 286** "And I had, I never had, as a teenager, I never had a boyfriend or anything… Because I was scared of people knowing. 'I don’t know what your feelings about it would be.'"  **Article 384** "My partner says to me 'You are special. You are not like all the rest'… It’s good that he thinks that it’s good that he’s met a girl who isn’t always on the go. So you can stay home and take it easy and not be out and about so much. Yes, it’s a good match."  **Article 402** "No man would want to marry a wife who falls down." "I feel ashamed because men have forcibly impregnated me three times, but no one will marry me, and they don’t want to support their children." "Several times men have approached me for marriage, but they never come back again. I have come to realize that the source of the problem is our neighbors who tell them that I am epileptic and they should not marry me. I really feel very bad." "I loved my wife very much, but she decided to abandon me because of my epilepsy after living together for about 40 years."  **Article 429** "I saw a TV program about epilepsy which said that a girl could not get married because she may genetically transmit epilepsy." "Also epilepsy can be more difficult for women in terms of getting married, go to school or finding a job."  **Article 449** "Most of my problems are due to people talking about me. I would like to marry again, but whenever someone shows interest, people talk to that man and tell him about my problem and so I haven’t had any offers of marriage."  **Article 455** "The first thing that they (family) start thinking about is 'Oh, we won’t be able to get her married off.'"  **Article 456** "It’s harder for women, because no one wants to get married with such women [with epilepsy], because women have to do housework and give birth. Those women cannot do such functions. Therefore men with this disease can still get married, while the women will have much difficulty." "I’m afraid that this disease would affect the whole of his life, especially looking for a girlfriend and marriage." |
| Ability to fulfil parental role | **Article 035** "… he was like, ‘leave your car in the car park’… I had kids to collect from school … they literally rip the end out of your world when somebody tells you that … what will I do with my kids? Do you tell them you’re not going to horse riding or you’re not going to football … you’re a mother."  **Article 115 supplement** "How do we continue to deal with this with the children?"  **Article 188** "I suppose I have been quite lucky because I, I mean with my brother’s family, I do look after the [children] … and they still let me do it, and every now and again I did go thinking, God, they could stop me … and I think if they literally said no you can’t do this anymore, … I think it would have totally made me go off the edge because it would have changed me too much."  **Article 207** "I will be able to look after my kids, just not having to have somebody here all the time, while my kids are here."  **Article 384** "I have seizures fairly often so I simply don’t dare be on my own with my children." "It doesn’t feel like you’re an adult, you can’t really go for a walk with the children … so you are sort of declared legally incompetent, in such cases it’s lucky, maybe one shouldn’t be alone in certain situations, but it can hurt that you can’t and shouldn’t take full responsibility." "God, I’m the world’s worst mum. Here’s me feeling, like, poorly and just, yes, but now I’ve had a seizure so now my kids have to manage by themselves because, like, I’m not well."  **Article 429** "I am afraid of holding my own children in my arms in case I have a seizure and let my child fall many times when I was holding."  **Article 441** "I can’t do that by myself, I have to wait until [my husband] is awake and able to supervise or whatever."  **Article 449** "In 2001, my husband divorced me because of my seizures. My child was only 1 year old at that time and my husband took my child when he left." "And maybe one day I will get angry and drop my baby. What kind of a mother cannot care for her own child? I don’t want relations with my husband and I might hurt my own child during a fit. Maybe one day I will just find my baby dead because I threw her away."  **Article 455** "A couple of times I’ve been feeding my baby daughter and I’ve had a fit whilst feeding her and she’s fallen on the floor and hurt herself." |
| Ability to fulfil work role | **Article 120** "‘Transferred from department to department’ and had been denied responsibilities at the factory where he worked ‘because they thought I would die at work.'"  **Article 185** "I wish they could ask me how I sit here at home, what I eat because I don’t work. Even when I ask for the grant they just say I haven’t reached 60…"  **Article 188** "I am still here and I can still do my job and yes, so for me, the negative diagnosis is still a positive way of living, yes."  **Article 207** "Yes [frustrating] because you are relying – I know it’s not important to most people – but you are relying on public transport…there is always cancellations, I mean it took me 3.5 hours to get to work the other day and I only work in [town] if I had my car it would have took me 20 minutes…I was so late for work….A lot of posts that are coming up that I apply for I can’t have because I have had my driving licence ceased, and that has been a huge impact on my life. Because now I have to walk a mile and half to the train station and get 2 trains to work, and then after work I get 2 trains to the nursery, to pick the kids up and spending 3 hours a day commuting."  **Article 310** "So they [employer] were really good, they explained my position, I couldn’t drive for a year, and the boss said, ok, we will see what we can do for you, and he said, why don’t we move you closer to the office and you give your walk to somebody else, so that’s what they done. Which helped me a lot, they helped me out."  **Article 384** "The hardest thing at work is having to write notes all the time so as to remember things, and as said, what you said, that if you’re taking something with you, you have to write it down or something so as not to forget it." "I’ve found it hard to concentrate. Mmm, if I’m at a meeting for example, I can’t concentrate for more than maybe 5 minutes if it’s something I really have to pay attention to because I have to report back to others and so I sit there with my notepad and I start to write and then just, no, and I start doodling, drawing stuff, ﬂowers."  **Article 402** "My working relationships are not good because my workmates always say I have mental problems."  **Article 441** "The computer screen sometimes causes my seizures, so I can only look at it about 20 min at a time ... so my work is really slowed down because of that." "I ride with a co-worker to work, and since I’ve had to slow down at work a little bit because of the meds, I’ve taken on different jobs that don’t wear me out, you know, to compensate for that."  **Article 456** "He still works. But it is said that we should not let him do heavy work, because if his brain had to work hard, fits of convulsion would happen. This disease is relating to the brain, so let him do light work. Hard work is not good for him." |
| Sense of social exclusion | **Article 137** "Suddenly people in a Christian surrounding, didn’t want to have anything to do with you."  **Article 205** "During childhood at school, when we were going to play football, my classmates said to the teacher, we don’t want him to play with us, and I cried and was angry. At parties, everyone looks at me in ways suggesting I should not be angry and not have a seizure and I don’t like this. So, I don’t like to go to parties."  **Article 208** "People do not actually like the person affected by epilepsy. They do not like to interact with him or her… No consideration for me at all. When I try to take interest in something, they ﬁnd that I am different from other people and should therefore refrain from interfering…"  **Article 332** "When I am on the street or in a market, people avoid me and warn each other to have less contacts or communication with me. They say to others not to talk to me at all because I am possessed by the devil."  **Article 384** "No, but you do such strange things, I have never met anyone with this, so you feel that, no, I’m the only one with this."  **Article 449** "And then my uncle said, 'If someone is bewitching you, better they should just kill you.' [stated before he threw her out of the family compound]." "Because of the epilepsy, people won’t sit next to me. Even my family has rejected me."  **Article 528** "But if you got epilepsy and you go somewhere and you tell them, you get blocked off. I’ve had experience of it."  **Article 531** "Yeah, the ﬁrst two years were just absolute, sheer hell, with losing your job, friends stay away... people seem to disappear." |
| Ability to leave the house | **Article 008** "That this disease isn’t going to prevent me from going out."  **Article 074** "So I have a real problem with my epilepsy in that it gave me agoraphobia […] I’m afraid to be on my own at night, I don’t go out on my own."  **Article 137** "I go out of the house and I meet the rest of the world and that in fact is a therapy in itself." "It’s not stopping me, I am going out."  **Article 158** "It does get to the stage, where sometimes when she has had more than one in a day, that’s it, the days, you can’t go out, you can’t do anything, so it has that impact as well."  **Article 216** "It makes me scared to go out. It is very hard. I don’t know when I am going to have a seizure – they come out of the blue. I don’t even want to go and get a cup of tea."  **Article 310** "They [primary care practice] got me a counsellor and she used to come once a week, and eventually she got me out the house and she would take me shopping and things like that, and then I gradually got out, and then it just progressed from there."  **Article 455** "I was very outgoing, I was into music and I used to be really outgoing, I used to enjoy myself, I was that type. Once I had epilepsy, I used to be worried and scared somebody might see me, so I used to stay in then, so I never used to go out in case somebody saw me." |
| Dependence on others | **Article 062** "My mom says to me ‘you have to have someone [near] to [help you] with your medication." "… I lived in a bubble all my life. I was not able to move on my own. I can’t go out because I depend on other people’s disposition, not on myself. I can’t go where I want."  **Article 137** "I usually do most of my heavy baking with the oven on when (husband) is around."  **Article 216** "He is with me every day as no one can ﬁnd anything for him to do. (Olivia, mother of 25 years old with TSC)."  **Article 238** "Epilepsy is a major barrier to finding and getting a job for most of us. As the saying goes, 'you can’t always get what you want.' We lose many things because of unemployment. We are dependent on our families, and cannot become independent. This is a big problem."  **Article 294** "Sometimes it makes me feel … as if I’m being doubted as far as my competency of doing certain tasks. I know she’s trying to be helpful and remind me of certain things, but sometimes it gets to me as far as making me feel as if I’m too dependent on her and I’m incapable of being on my own." "I get aggravated because [my mother is] always around, helping me, and I just want to do everything on my own. But I understand that I can’t now, and she’s there for me. But, yeah, I get aggravated sometimes. I get tired of her always helping me, but I know it will get better."  **Article 320** "We’ve been together 40 years… real independent. But now… [my husband] hovers. I went from being wife… to child."  **Article 384** "Wherever I go, if I get in the shower, I text my mother, like, I’m getting in the shower now. Because she, she panics if she calls my house and I don’t answer." |
| Ability to have children | **Article 188** "I have had everything there is to have, kids that I have done a lot with, sport, everything else, and I don’t really think you can have a better quality of life to be quite honest."  **Article 204** "Few times I have decided to get married, but as soon as they’re informed of my illness, my proposal of marriage is rejected. When I asked why? They told me you have a contagious disease or we cannot have children because of your illness."  **Article 280** "If there was any more than say a 40% chance for instance… then I would have to… make a decision on whether that would be a chance I would be willing to take. If it was a very high possibility, unfortunately it would probably sway my mind not to, which would be very hard cos I’d love to have kids."  **Article 384** "These choices you make, like having children, what work to do, how much to work, there’s so much, everything becomes so tough. You can’t just do what everyone else does, nah, but now I want to."  **Article 439** "Because I was actually planning to have a kid, or try and get pregnant and if I did I wouldn’t want the Epilim to do anything, because I know there’s a high chance that the Epilim would have done something to my child if I had got pregnant."  **Article 449** "We [meaning WWE] should not have children. The seizures can get worse when we are pregnant and the work of being a mother can also worsen this problem." |
| Change in social activities due to seizures | **Article 074** "Say I’m going from home to the shops, I always walk one of two or three routes, so if anything was to happen, say I was to lose something as a result of the seizure, well, it’s got to be in that direction. Other things like, so if I’m crossing the road I always use a pedestrian crossing, cross at the lights, always on the green light, so if anything was to happen, I’m covered."  **Article 200** "But I am troubled with nausea and cannot eat a meal with my family which affects my daily social life…"  **Article 397** "…at school, in the classroom, I always sit in the back row, so I won’t panic …when it becomes clear that it would be difﬁcult to get out – that is when I panic… thinking I can’t hurry out if I feel a seizure might come."  **Article 429** "My parents restrict me from going into the kitchen or going outside to play. I am not allowed to go near water or to go fishing."  **Article 455** "I was very outgoing, I was into music and I used to be really outgoing, I used to enjoy myself, I was that type. Once I had epilepsy, I used to be worried and scared somebody might see me, so I used to stay in then, so I never used to go out in case somebody saw me." |
| Psychosocial burden on others | **Article 062** "The thing is, I think of my daughters and I don’t like them to see me like this. I think of them, in my family, in my whole family. Because I know I’m not the only one who suffers, but… in fact, I think my family suffers more than me."  **Article 074** "My mum was a really big worrier and I think when she used to worry so much about things like, about the seizures, that sort of made me feel kind of worried." "It scares my parents as well. […] Mum’s gone out of her way, I guess, to try and keep me here."  **Article 115 supplement** "No, you don’t burden them now, they have enough on their plate."  **Article 137** "It’s frightening for them, he (husband) hadn’t experienced anything like this before so he was anxious."  **Article 216** "His sibling has missed out on so much because I have had to pass them around from pillar to post for other people to look after them - I had to be so focused on him."  **Article 320** "Because we are way out here in the country… we are at the mercy of neighbors to pick things up for us."  **Article 384** "My husband has been unemployed for a long time and it’s really difﬁcult for him to ﬁnd a job. Because he feels he has to explain our family situation every time he’s called to an interview … I feel really guilty. He’d have a job if things weren’t like this."  **Article 397** "They worry all the time……for example if something falls down hitting the ﬂoor, at once they come running, asking me what is happening, am I alright."  **Article 403** "Psychological stress, as in the case of F-2, whose most recent 'frightening seizure' activity required hospitalization and was witnessed by her 5-year-old son." "I think you know there is not enough awareness out there for kids or siblings (of PWE) regarding seizures." |
| Acceptance by others (no stigma) | **Article 188** "I sometimes talk about it to friends and they kind of say you know, your epilepsy is not a big deal, we never notice it, and if we do it’s no big deal, if anybody else was to notice it they probably wouldn’t think anything of it, and you shouldn’t let it constrict you in terms of where you want to go in the future, because things are always achievable… and then you kind of put it into perspective." "But in terms of my social life, not at all [impact]. Because obviously my friends are really understanding about it and although I feel quite embarrassed when I have these seizures, they are all kind of like whatever you know it doesn’t matter."  **Article 238** "Constructive cultural changes have to be made to improve the public’s attitude about epilepsy, so that I can find a job somewhere and openly say 'I am an epileptic' without hesitation. We need better understanding of this disease to hire patients, offer them a lighter and less risky assignment, matched with their ability and diagnosis; instead of rejecting them. Public awareness and education is a crucial strategy."  **Article 286** "They seem to be cool with it. Understanding and everything. They’ve got no problem with it."  **Article 310** "But they [employers] have been absolutely fantastic, when I went back to work, they said take all the time you need to recover you know from the seizures, and when I went back in and going on the medication, I explained to them look this is what is happening and you know there are warnings that I might become very, very tired, and they said fine, just work until whenever, when you feel tired just go home, you know and there was no sort of like issues." |
| Exclusion by family | **Article 140** "At home, they treat me well, but my other relatives treat me badly. My father and my brothers no longer care about me, they don’t want to help me with the health treatment anymore."  **Article 208** "I am excluded from what happens in the family. I have even been forbidden from managing my income on the ground that I am unable to do it."  **Article 449** "And then my uncle said, 'If someone is bewitching you, better they should just kill you.' [stated before he threw her out of the family compound]." "Because of the epilepsy, people won’t sit next to me. Even my family has rejected me." |
| Ability to develop a career / profession | **Article 384** "These choices you make, like having children, what work to do, how much to work, there’s so much, everything becomes so tough. You can’t just do what everyone else does, nah, but now I want to." "I really wanted to become a policeman but that doesn’t work and that kind of things really makes me mad." "Yes, many times I’ve thought now that things have gone so badly for me. At school if I, if I were to be free of this. What would it be like for me with a job if I were to become completely healthy and normal, how would things be then? What should I do about work and stuff like that."  **Article 402** "I am not happy because I can’t fulﬁll my vision. I want to be a doctor but I can’t attend school on a regular basis due to my condition."  **Article 429** "People with epilepsy have no future and cannot go to school." |
| Sense of belonging (others have epilepsy too) | **Article 144** "So it’s suddenly all of an ‘eye-opener’ and talking here you realise we are not on our own."  **Article 146** "I felt like I was the only one dealing with it really, and it was getting me down... But when I heard that other people have got it, and I met the other people who have got it, I kind of changed my mind... I look at it in a diﬀerent way."  **Article 501** "But with websites, they’ve always been very informative. The thing I’ve enjoyed the most with websites have been chat rooms really, because they’ve always [been] very interesting when you can actually get out there and speak to people that have got the same condition as you have. Especially when you have the opportunity to speak to people that maybe are on the same drugs that you are, and they speak about the conditions and the side effects that they may have." |
| Ability to go on holiday | **Article 074** "I loved travelling, I was planning to go back overseas again, and now I don’t think I should do that in case I have a seizure."  **Article 216** "We have been on holiday for the ﬁrst time in years … and it was a great break for us all." "[Seizure management] has given us a bit of family life back. I can now have holidays. I can have a relationship with my partner. His sibling can have time with me now … It’s been completely life-changing. We are now a functioning family as opposed to a family in a state of constant medical crisis." |
| Impact on gender identify | **Article 084** "It also means something to me. To feel like a man."  **Article 332** "Indeed I worry about my ability as a man and feel responsible to provide for my wife and fulﬁll her needs. This is very hard for me and any man." |
| Ability to make new friends | **Article 286** "A girl of your age doesn’t want to come around you. She believes, 'If I go around, playing with her, then I’m going to get what she’s having.' Parents do tell their kids, 'Don’t go there.'"  **Article 384** "Say you meet some new people that you haven’t met before and they ﬁnd out you have epilepsy, well then they take 3 steps back, sort of. If you’ve met them 5 times before you tell them you’ve got epilepsy, well then some of them don’t back away." "It’s hard to make friends when you are going to explain, you have to say that you’ve got epilepsy and some people react in a way that you don’t want them to and some of them are like … I’m here for you and I’ll do anything for you … don’t pity me, that’s not what I want, just be a friend." |
| Burden from not driving | **Article 074** "Other people don’t feel like a burden but I feel like a burden […] Most of the time the car thing is the biggest thing that causes me to be a burden on others."  **Article 169** "I hate that I can’t just drive and just get home. The same from swimming… If I could get to swimming events on my own… But I don’t, I have to rely on everyone."  **Article 207** "Yes [frustrating] because you are relying – I know it’s not important to most people – but you are relying on public transport… there is always cancellations, I mean it took me 3.5 hours to get to work the other day and I only work in [town], if I had my car it would have took me 20 minutes… I was so late for work…. A lot of posts that are coming up that I apply for I can’t have because I have had my driving licence ceased, and that has been a huge impact on my life. Because now I have to walk a mile and a half to the train station and get 2 trains to work, and then after work I get 2 trains to the nursery, to pick the kids up and spending 3 hours a day commuting." "I might try without medication but if another seizure results then I can’t drive again for at least 12 months, and if I have got a young child [husband] works away a lot, and I haven’t got a vehicle how does that impact me? You know, my family don’t live here, they all live far away so I can’t rely on family to help me get around— so it sounds daft, but one sort of small thing like that has so many ramifications… certainly from my point of view it’s not a hugely difficult decision because I don’t really have any side effects on the medication."  **Article 453** "It means giving up my car for six months, and that means a lot socially, how am I gonna... get about and everything." |
| Burden on caregivers | **Article 079** "He was not willing to come here today at first. But I begged him and he came. This is because he has work and he has children…"  **Article 144** "It can be quite overwhelming I think for your partners. It’s like ‘all on their shoulders’ what happens. I think your carer needs a lot of support too."  **Article 216** "He is with me every day as no one can ﬁnd anything for him to do. (Olivia, mother of 25 years old with TSC)." "It’s ongoing. There isn’t any end date where we can stand back and think well we’ve done it now. The impact is huge."  **Article 429** "Family has suffered a lot of hardship looking after my daughter who has had condition from birth and is now 41 years old. My daughter requires total care - toileting, meal preparation etc. She is totally dependent on other adults." |
| Burden on friends and family | **Article 074** "I worry about my family, […] and I think I annoy [my family] and my friends because they have to take care of me and things like that." "But I’ll certainly have someone to watch me; I’ll have a spotter, and so if I truly feel it’s not safe I’ll get somebody else to do it."  **Article 115 supplement** "I myself am someone who always does everything for others immediately […], but I never demand this from others." "Initially, it was really difficult for me to accept help or ask for help."  **Article 139** "I know for me I wouldn’t have a neurologist realistically, or wouldn’t’ve ended up going if it weren’t for the support of my mom and grandmother […] it can be a really difﬁcult thing to do alone." "I just feel like an inconvenience constantly."  **Article 169** "You’re doing this, and you shouldn’t really be doing that. It makes me think, 'Yeah, maybe if I do go swimming or the gym on my own, maybe I should go tell someone?' But I don’t want to tell someone that I’m swimming because then I have got to have some idiot watch me swim, and I don’t want that."  **Article 204** "I was a construction worker. Few times at work, I had seizure and for this I was kicked out by my employer. I often get a negative answer for my illness wherever I refer to for employment. I decided to stay at home. Actually, I’ve become a burden on my family."  **Article 207** "I will be able to look after my kids, just not having to have somebody here all the time, while my kids are here."  **Article 216** "He is with me every day as no one can ﬁnd anything for him to do. (Olivia, mother of 25 years old with TSC)." "His sibling has missed out on so much because I have had to pass them around from pillar to post for other people to look after them - I had to be so focused on him."  **Article 294** "How thankful I am that God provided me with somebody that he knew I was going to need and that has the heart to do this … It has its plusses and weaknesses at times. We all get tired and she gets tired, not only with me, but her job … So that’s hard on me to see her being tired about anything and can’t step up to the plate and just take care of it because of this unfortunate illness." "Well sometimes you feel alone, you know. You’re the only one that’s doing it … she doesn’t want to be a burden on anybody, and, you know, she and I have an agreement that I’m it."  **Article 320** "It’s a burden."  **Article 429** "I am a burden to my family because they have to take care of me and they worry about me."  **Article 455** "My husband, he’ll kind of stay awake during the night to make sure I’m okay. So during the day he gets tired so he goes to sleep and he can’t work because of that."  **Article 494** "I mostly depend on my in-laws who live here to get me where I need to go. But they have things to do and I can’t ask them all the time. They’re struggling, too, so they can’t help me get what medicine I need either. So, I do what I can do, like getting what I can get and going where I can go. That’s about all I can do." |
| Inability to drink alcohol socially | **Article 460** "The few things that remind me and annoy me about this situation are the alcohol and driving license restrictions and the obstacles to serving in the army like anyone else." "I really want to get drunk just once in my life." |
| Career progression | **Article 074** "I’m reluctant to get involved in big things where you’ve got to stand up and, you know, when I’ve got to speak in a group and all that." "So I don’t think I’d be going for any higher jobs and I put it down to the epilepsy." |
| Involvement with law enforcement agency due to seizure related behaviour | **Article 074** "I’ve been asked to leave places because they thought I’ve been intoxicated. Once, coming out of a ﬁt, that’s like, I’ve had dealings with police on New Year’s Eve, when you go to a pub." |

G. LIFE IMPACT OUTCOMES, INCLUDING GLOBAL QUALITY OF LIFE

| OUTCOME CODE | VERBATIM PARTICIPANT TEXT |
| --- | --- |
| HRQOL not otherwise specified | **Article 276** "My access to epilepsy services and the services I receive are excellent. I can see my consultant when it is needed and my GP is both understanding and informed. My consultant is excellent. My epilepsy is difﬁcult to control and all efforts I feel are being made to get the best quality of life for me."  **Article 310** "I mean my main aim in life was working as a [work role], but it [QOL] has certainly changed because they finished me because I couldn’t get control of my fits … I was absolutely devastated… it has took me years to get over it."  **Article 460** "At the age of 17, I considered my quality of life to be a 95 on a scale of 0-100. But at the age of 22, I feel it to be more like an 80 due to my perception of the consequences of epilepsy." |
| Financial impact of medication | **Article 103** "I live on AISH [Assured Income for the Severely Handicapped] and ... when they don’t have [Frisium] I have to drive around everywhere looking for it and it costs me gas, but I don’t have the money ..."  **Article 320** "I’m okay until I get to a donut hole. Then I have to dip into savings." "Sometimes it’s like pay the electric or get my medicine…. I want my lights on." "Taking a half dose of my [AED] helps in terms of it’s better… than not taking any until I have money. But it’s not good."  **Article 332** "I do not have health insurance coverage and a physician’s visit is very expensive. Drugs are expensive and hard to ﬁnd. I do not go to the specialist and instead go to a general practitioner who is less expensive and ask for a reﬁll on my previous prescription given by the specialist. I do not go to my physician very often because of the cost. For my prescription I go to the specialist only once a year. I ask him to explain how to change my dosage every month and try to learn how to adjust my medications myself every month and save money."  **Article 440** "[Epilepsy] is heavy on the economy, [wife] doesn’t work and I laid off, all my family rely on the subsidy. The drug takes 100 yuans per month, and if unfortunately we should go to the hospital the subsidy will be used up."  **Article 494** "I went to the pharmacy to buy the medication. After ﬁlling the prescription, the pharmacist or pharmacy tech, I’m not sure which one she was, said 'that will be five-ninety-one [$5.91].' So, I gave her a ten-dollar bill to cover the charge. But she said, 'No, that will be $591.00.' I was really in shock when she said that, but I told her that I couldn’t afford it. I just went home and cried."  **Article 532** "She sent me a form through, as my doctor didn’t tell me that I didn’t have to pay for them (medication)."  **Article 540** "She sent me a form through, as my doctor didn’t tell me that I didn’t have to pay for them (medication)." |
| Financial security | **Article 084** "We won’t have to go on disability or sell the house, you know, when we know we will survive, money, security and so on."  **Article 185** "I wish they could ask me how I sit here at home, what I eat because I don’t work. Even when I ask for the grant they just say I haven’t reached 60…"  **Article 188** "I would describe it as being comfortable, erm, happy, or at least contented, having no money worries." "I was on a real downer…, there was everything, one on top of the other, these forms, and money worries, I had just fallen out with my buddy up there."  **Article 339** "And I did receive help dealing with government agencies—Help with work training, for example, and help with the many contacts I had with the employment agency and the insurance ofﬁce, which can be pretty challenging—all the certiﬁcates and so on."  **Article 455** "So I sat down with my older brother and said to him, 'Look, this is the situation, I can’t stay on income support all my life. I want to save some money and, you know, buy my own house.' And he goes to me, 'Just forget it, forget work, don’t worry about it, we’ll buy you a house.'"  **Article 494** "Getting epilepsy has put me in poverty." |
| Financial impact of not working | **Article 185** "I wish they could ask me how I sit here at home, what I eat because I don’t work. Even when I ask for the grant they just say I haven’t reached 60…"  **Article 238** "Epilepsy is a major barrier to finding and getting a job for most of us. As the saying goes, 'you can’t always get what you want.' We lose many things because of unemployment. We are dependent on our families, and cannot become independent. This is a big problem."  **Article 429** "Epilepsy is more difficult for men because men are the workforce of the family. If a man does not work, it would be a burden."  **Article 441** "When I was ﬁrst diagnosed I had no way to get to work, and that made me so uptight and worried. If I didn’t get to work I couldn’t pay my bills, and my whole family would suffer. So I just drove anyway because I had to so that we could live normally."  **Article 449** "My marriage has changed. I don’t work now and cannot bring money like before. And because of my epilepsy, I can’t find work . . . because my condition is known, no one will hire me." |
| Financial impact of hospital attendance | **Article 440** "[Epilepsy] is heavy on the economy, [wife] doesn’t work and I laid off, all my family rely on the subsidy. The drug takes 100 yuans per month, and if unfortunately we should go to the hospital the subsidy will be used up." |

H. HEALTH RESOURCE USE OUTCOMES

| OUTCOME CODE | VERBATIM PARTICIPANT TEXT |
| --- | --- |
| Need for hospital or emergency department attendance | **Article 276** "My access to epilepsy services and the services I receive are excellent. I can see my consultant when it is needed and my GP is both understanding and informed. My consultant is excellent. My epilepsy is difﬁcult to control and all efforts I feel are being made to get the best quality of life for me."  **Article 310** "I mean my main aim in life was working as a [work role], but it [QOL] has certainly changed because they finished me because I couldn’t get control of my fits … I was absolutely devastated… it has took me years to get over it."  **Article 460** "At the age of 17, I considered my quality of life to be a 95 on a scale of 0-100. But at the age of 22, I feel it to be more like an 80 due to my perception of the consequences of epilepsy." |
| Unnecessary hospital attendance | **Article 058** "I didn’t have a cluster of seizures or that my heart slowed down (it has previously after big seizures). My husband and I told the ambulance and the ED doctor I just needed to sleep it off."  **Article 358** "There’s not a lot they can actually do. Most times on arrival, you’re aware, and they cannot offer you anymore; wasting space for somebody else who can go ahead and use that." "Injury is the only cause I see for medical attention really to be checked over. If it’s something minor, it doesn’t need medical attention, it wastes hospital time." |
| Need for emergency ambulance | **Article 358** "Because it had been quite a while and because I hadn’t presented with vomiting before. So it was obviously the new dimension of it that led me to call the ambulance." |

I. SIDE EFFECT OUTCOMES AND DRUG MONITORING

| OUTCOME CODE | VERBATIM PARTICIPANT TEXT |
| --- | --- |
| Medication side effect (general) | **Article 008** "I feel sick, like that time when I felt bad from the Keppra."  **Article 035** "My previous neurologists … just kept on medicating, medicating, medicating until I said to him, ‘I can’t do this, I can’t talk [as a side-effect of AEDs].’" "So, I said an increase of medication would have more serious consequences than one more seizure, so I would wait… so I can make my own choices regarding my condition… I then didn’t have to cope with adjusting to a higher dosage."  **Article 185** "I am still feeling dizzy when I take these pills and I have no strength… I would really like that they explain about why the medication doesn’t get used to me…"  **Article 253** "Experiencing 'the shakes' as a side effect of a particular medication saying, 'I preferred to have the seizures.'"  **Article 276** "After 18 years of having epilepsy I’ve just got my ﬁrst 12 months seizure-free but feel the constant increase and switching of medication made little difference except bring on side effects."  **Article 310** "And actually I would rather in some respects just get rid of the side effects and accept the seizures, because I have got sort of used to the seizures, as have friends and all this."  **Article 369** "I found more about [er] sort of side effects of tablets, which made me more [er] paranoid about the side effects which brought the side effects on more."  **Article 453** "… there is a lot of side effects ... tremors, shaking ... poor concentration ... erm, mood swings ... erm ... change in appetite, change your erm sleeping patterns." "… what I found was the side effect of the medication was worse than the seizures."  **Article 531** "Side effects of the medications."  **Article 567** "One of the things that made me so irritated at the beginning was that I would tell my doctor all these side-effects and he said ‘well what do you want, ﬁts or side effects?’ And you almost want to walk out; ‘well, excuse me, I don’t want to have either here. This is hard enough as it is.'" |
| Medication related somnolence | **Article 033** "To me it was a greater risk for me falling asleep, like going on the train and visiting people ‘cos if I go on a train I’d sleep past the stop, I know it would… I mean yes I still need a sleep but I’ve got more control, it’s like I can have a sleep when I want to have a sleep and so now I can plan it, as opposed to this awful like drop sleep… it enables me to plan more and do more in my life." "I said no, I’m not going to take that because it knocks me out enough already, I don’t want to be a zombie."  **Article 062** "I had to take the medication in the afternoon during my class; then, at a particular moment, I started feeling a little sleepy. After the first half an hour I began to feel sleepy."  **Article 207** "Side effects of tiredness and mood swings and irritability pale into insignificance compared to worrying about making an idiot of yourself in the town centre." "Try and introduce one more [tablet], I literally have tried but I would be in bed by 6.30 before the kids actually go to bed… I don’t think that’s a life really… so the best medication is the one that is going to have less impact on your life."  **Article 310** "The worse thing about the tablets themselves that I am on, and they do stop a lot of the big fits, is they just make you so tired, you cannot live a normal life on them, to a degree I find that it’s very, very difficult because you are tired all the time. You know… that is the big depression."  **Article 320** "I’m an avid reader… but on that medication I fall asleep."  **Article 328** "I was just so ill, two years I was [on topiramate]. I lost so much weight and I’m small anyway, my hair was falling out, spots and everything… but it must have been the medication that’s all I can think of. I was so tired all of the time I was just really ill… I was very reluctant because of the experience in the past it frightened me and put me off I was reluctant."  **Article 384** "Then there’s energy when you’re working, there’s less when working like this. I only work 50% because I get really tired from all the medicine I have to take." "The medication gave me lots of side effects, so I was tired, I’d start crying at school, I couldn’t read."  **Article 441** "That medication makes me so sleepy... that’s why I don’t take it if I have to stay up and write a paper or have an early class."  **Article 531** "Here I am, falling asleep at work with this new medication." "I don’t like opening my body. No I don’t like having surgery. I would rather be drowsy." |
| Medication related weight gain | **Article 033** "Once I was told I’d put on weight, and it was either well, take your medication and keep all your weight, or stop your medication and have your seizures…"  **Article 062** "I don’t know if it’s because my body is adapting to Logical (Valproate), but it also makes me gain a lot of weight. I can’t see myself anymore. Because this pill has something that makes you fat. I don’t remember the name. I don’t remember. And if I’m not under constant physical training it makes me fat and lets me down. I eat a lot because I’m big."  **Article 200** "I gained a lot of weight with valproate alone, but with the addition of topiramate, which may lead to a decrease in body weight, this is unstable and goes up and down." "I gained weight with valproate and lost weight with topiramate."  **Article 207** "The side effects of the Epilim has just knocked me for six… because the weight gain… it is just so depressing, it really is, and it’s unbelievable what it does to you. It does, you know what I mean, it knocks your confidence."  **Article 230** "When you write that you have lost your libido and you have gained weight, then what happens?"  **Article 328** "Tiredness and obviously the increase in the seizures… again it’s tiredness but… I gained a lot of weight." |
| Medication related mood changes | **Article 200** "I used lamotrigine previously but had to withdraw it due to many adverse effects. I was indifferent to everything, my emotional life was flat, and even my friends noticed it. When I stopped everything, I was normal again."  **Article 221** "I found myself getting angry, so I was finding out about the whole Keppra rage thing and seeing whether other people got it."  **Article 384** "The medication gave me lots of side effects, so I was tired, I’d start crying at school, I couldn’t read."  **Article 453** "There is a lot of side effects... tremors, shaking... poor concentration... erm, mood swings... erm... change in appetite, change your erm sleeping patterns."  **Article 567** "A lot of my fits are caused by depression, which [is] caused by the amount of drugs I am taking, which counterreact with antidepressants." |
| Medication related cognitive disturbance | **Article 207** "Then problems remembering things… I have to say doesn’t really affect me at all but, I would start to get very worried if I started not being able to remember things… it would just make me feel like my brain was going really. If that started happening, I would seriously have to consider… what is going on to be honest."  **Article 384** "Because I think the medication is worse for my memory than the seizures are. I’ve noticed the difference when changing from Tegretol to more advanced stuff. It just got worse and worse then, I had more and more syrup in my head."  **Article 440** "Now his memory is slow and he stutters."  **Article 453** "There is a lot of side effects... tremors, shaking... poor concentration... erm, mood swings... erm... change in appetite, change your erm sleeping patterns."  **Article 531** "I am concerned about the long-term effects of his medications and his abilities. His school grades have deteriorated over the years... He is either drugged out or exhausted from having seizures." |
| Medication related fatigue | **Article 033** "I didn’t want to be overdosed. I’m feeling that tired all the time."  **Article 079** "I became tired… I become exhausted. That is it."  **Article 185** "I tell the doctor about my fatigue… but I don’t know if they take note of that or if they don’t."  **Article 441** "I have absolutely zero energy in the mornings after I take my medication at night." "If I don’t take my pills for a few days, the fatigue goes away completely and I have three days of energy where I can get work done, work on my house or whatever, and then go back on them to keep from having a seizure. I admit sometimes I don’t take my pills for that reason." "It helps with dealing with the fatigue from the meds, and I read online that it helps prevent seizures." |
| Impact of medications on offspring health | **Article 074** "And I’ve had my kids, so I would be worried about being on medications that would affect your baby."  **Article 439** "Because I was actually planning to have a kid, or try and get pregnant and if I did I wouldn’t want the Epilim to do anything, because I know there’s a high chance that the Epilim would have done something to my child if I had got pregnant."  **Article 451** "No one knew about it. ... I was having cluster fits and the medication was put up and up and up. I was really scared that there would be some effect on the baby. I just thought, 'I’m having the fits anyway, I’ll have them but at least I won’t be taking the tablets; the tablets won’t be doing anything to the baby even if the fits are.'"  **Article 567** "I saw Dr X when I was pregnant with my little girl and he said to me I was on the best drug for pregnant women – that’s Epilim. He said, 'anybody who is wanting to become pregnant, we’re putting them all on sodium valproate.' L: Everybody is on Epilim that’s pregnant. S: And when L said to me she was on Tegretol I was a little bit surprised, but obviously there is a reason, perhaps that’s the one that controls her fits, I don’t know. L: I’ve asked him why and, I think it’s like, they don’t want to get into too much detail. You know someone [to S] who was taking Tegretol who had a blind, deaf and dumb child." |
| Medication related weight loss | **Article 062** "They all were beautiful, with beautiful bodies; I was like a stick, horrible. I was living under medication."  **Article 200** "I gained a lot of weight with valproate alone, but with the addition of topiramate, which may lead to a decrease in body weight, this is unstable and goes up and down." "I gained weight with valproate and lost weight with topiramate."  **Article 328** "I was just so ill, two years I was [on topiramate]. I lost so much weight and I’m small anyway, my hair was falling out, spots and everything… but it must have been the medication, that’s all I can think of. I was so tired all of the time I was just really ill… I was very reluctant because of the experience in the past it frightened me and put me off. I was reluctant." |
| Medication related irritability | **Article 200** "I was very annoyed when I used levetiracetam but feel better now as I use valproate, even if I feel blue periodically. I experience seizures from time to time, but usually this happens only when I have not taken my medication for some days."  **Article 207** "Side effects of tiredness and mood swings and irritability pale into insignificance compared to worrying about making an idiot of yourself in the town centre."  **Article 221** "I found myself getting angry, so I was finding out about the whole Keppra rage thing and seeing whether other people got it." |
| Medication related aggression | **Article 008** "Every time I took the medicine I became aggressive. Fighting with my friends... Then I told them, ‘It’s not me, it’s the medicine that does this.’"  **Article 207** "I was taking lamotrigine and it wasn’t working by itself, so they put me on another drug…. I had no idea that I was so — I think aggressive would be the word, until… [business partner] turned round and said I can’t deal with you snapping at me anymore... I said right ok I will come off it… I felt absolutely awful, as you can imagine, which is why I came off the drugs so I have been a lot more ill… but I don’t care, quite frankly as long as we are ok."  "Feelings of anger or aggression — I think that maybe I wouldn’t want to have that problem… it wouldn’t be a bit fair on my wife, she would take the brunt of it wouldn’t she." |
| Medication related excess sweating | **Article 053** "I am finding that I am sweating a lot” |
| Medication interactions | **Article 501** "And it was only that I found out about that that the dose was increased. So potentially I could have become pregnant at a time which wouldn’t have suited me... I would have liked more information on that."  **Article 567** "[It] just stopped working. I could have got pregnant so easily within the ﬁrst couple of months [of starting AED medication] because I had no idea. I kept bleeding in between times, I thought what is going on, I have never had this before." "A lot of my ﬁts are caused by depression, which [is] caused by the amount of drugs I am taking, which counterreact with antidepressants." |
| Medication related anxiety | **Article 200** "I am struggling with anxiety and cannot look people in the eyes. I feel like I lost my spark I used to have and suspect it might be due to the medication."  **Article 207** "Obviously the side effect of this tablet now, it is an increased dosage but it’s stopped, well, it’s decreased my seizures — but obviously the result of that is obviously the anxiety… you will get that with the Keppra and it’s a known side effect… it’s not a nice thing in general, anxiety… you want to get on with things and you want to do things, and you just, but it’s building up." |
| Medication related osteopenia / osteoporosis | **Article 216** "He has osteoporosis due to long-term use of epileptic drugs and has fractures during seizures. He has broken vertebrae and ribs during seizure-related falls."  **Article 501** "It was only when I changed my doctor, and got to see the consultant I’m seeing now, did anyone bother telling me that actually phenobarbitone withdraws calcium from the bloodstream... and especially after the menopause there’s a strong risk of osteoporosis. And I was furious that information hadn’t been given to me sooner." |
| Medication related dizziness | **Article 185** "When I told him I feel weak. I just become dizzy sometimes when I take these tablets… He said that it is something that is common."  "I am still feeling dizzy when I take these pills and I have no strength… I would really like that they explain about why the medication doesn’t get used to me…"  "I have been taking these medicines since I was young… I noticed this thing that every time I take these pills I feel dizzy… I would feel like someone who had taken an alcoholic drink and I don’t even touch that… I want to know why I don’t get better…" |
| Medication related alopecia | **Article 328** "I was just so ill, two years I was [topiramate]. I lost so much weight and I’m small anyway, my hair was falling out, spots and everything… but it must have been the medication that’s all I can think of. I was so tired all of the time I was just really ill… I was very reluctant because of the experience in the past it frightened me and put me off I was reluctant." |
| Medication related appetite change | **Article 453** "… there is a lot of side effects ... erm ... change in appetite” |
| Medication related headaches | **Article 441** "One of the medicines I take gives me headaches and makes me vomit sometimes" |
| Medication related nausea | **Article 200** "I have been seizure free for some months with lamotrigine and topiramate. But I am troubled with nausea and cannot eat a meal with my family which affects my daily social life…" |
| Medication related personality change | **Article 531** "The medication doesn’t make me feel like myself. It makes me schizophrenic, basically. Taking the medication did not stop me from having the seizures. I am having just as many seizures then as I am now." |
| Medication related seizures (paradoxical) | **Article 567** "A: I have to say that now they seem to be willing to ﬁddle rather than just pump more and more drugs into you. R: Fiddling in terms of up or down you mean? A: Yes, and also taking you off one drug and putting you on another. I mean at one time I was on about 5 drugs. Fiddling is much better than just pumping in more, because I was having side-effects with the drugs and also having seizures because of the drugs rather than the epilepsy." |
| Medication related sleep disturbance | **Article 453**  "… there is a lot of side effects ... change your erm sleeping patterns."​ |
| Medication related vomiting | **Article 441**  "One of the medicines I take gives me headaches and makes me vomit sometimes, so I always take it with crackers."​ |

J. PREGNANCY AND OFFSPRING OUTCOMES

| OUTCOME CODE | VERBATIM PARTICIPANT TEXT |
| --- | --- |
| Ability to get pregnant | **Article 185**  "I asked the doctor if a person with epilepsy [meaning a female person who has epilepsy] can get pregnant. That doctor said yes and it ended there…"  **Article 449**  "Every time I am pregnant the seizures get worse. If I fit when I am pregnant, then maybe my child will have this problem, too."  **Article 567**  "I saw Dr X when I was pregnant with my little girl and he said to me I was on the best drug for pregnant women – that’s Epilim. He said, ‘anybody who is wanting to become pregnant, we’re putting them all on sodium valproate’."  "Everybody is on Epilim that’s pregnant."  "And when L said to me she was on Tegretol I was a little bit surprised, but obviously there is a reason, perhaps that’s the one that controls her ﬁts, I don’t know."  "I’ve asked him why and, I think it’s like, they don’t want to get into too much detail. You know someone [to S] who was taking Tegretol who had a blind, deaf and dumb child." |
| Impact of seizure on foetal health and development | **Article 207**  "Wanted to reduce the risk of anything happening to the baby. But I didn’t want to, you know, like double my chance of having a fit and losing the baby altogether, if you know what I mean, so I sort of had to balance baby over me."  **Article 449**  "Every time I am pregnant the seizures get worse. If I fit when I am pregnant, then maybe my child will have this problem, too." |
| Unintended pregnancy | **Article 501**  "And it was only that I found out about that that the dose was increased. So potentially I could have become pregnant at a time which wouldn’t have suited me...I would have liked more information on that." |
| Ability to breastfeed | **Article 449**  "Maybe if I breastfeed, there will be problems." |

K. DEATH AS AN OUTCOME

| OUTCOME CODE | VERBATIM PARTICIPANT TEXT |
| --- | --- |
| Mortality general | **Article 079**  "I was very dreadful because I felt like the illness might push me into a fire and it might kill me and I still feel dreadful."  **Article 084**  "When the shock of diagnosis has eased and we know we’re not going to die."  **Article 310**  "I used to have three jobs at that time, and as soon as epilepsy hit me I gave them all up — thought no, I can’t work, I am terrified in case I have a fit…because I mean when I fell on the concrete I split all my head open and I had to have stitches inside and outside, I mean, I could have died on that floor.... I gave up all my three jobs, I had to give up my bike — and yes it was really… I don’t know really how I did cope really, because I felt so isolated."  **Article 501**  "It’s just quite scary to think that one night we, any one of us, might just go to sleep and never wake up [due to the epilepsy]" |
| SUDEP specifically | **Article 358**  "I was afraid I might die, because [the epilepsy] could kill."  **Article 501**  "It’s just quite scary to think that one night we, any one of us, might just go to sleep and never wake up [due to the epilepsy]." |

**Additional references of included studies**

1 Andersson K, Shadman A, Strang S. Trustful communication in the medical encounter: Perspectives of immigrated people with epilepsy. *Chronic Illn* 2019. DOI:10.1177/1742395319846254.

2 Kılınç S, Campbell C, Guy A, van Wersch A. Negotiating the boundaries of the medical model: Experiences of people with epilepsy. *Epilepsy and Behavior* 2020; 102: 106674.

3 Power R, Byrne JP, Kiersey R, *et al.* Are patients ready for integrated person-centered care? A qualitative study of people with epilepsy in Ireland. *Epilepsy and Behavior* 2020. DOI:10.1016/j.yebeh.2019.106668.

4 Ninnoni JPK. A qualitative study of the communication and information needs of people with learning disabilities and epilepsy with physicians, nurses and carers. *BMC Neurol* 2019. DOI:10.1186/s12883-018-1235-9.

5 Peterson CL, Walker C, Coleman H. ‘I hate wasting the hospital’s time’: Experiences of emergency department admissions of Australian people with epilepsy. *Epilepsy and Behavior* 2019. DOI:10.1016/j.yebeh.2018.11.018.

6 Sarudiansky M, Korman GP, Scévola L, Oddo S, Kochen S, D’alessio L. A life with seizures: Argentine patients’ perspectives about the impact of drug-resistant epilepsy on their lives. 2018. DOI:10.1016/j.seizure.2018.10.008.

7 Kılınç S, Campbell C, Guy A, van Wersch A. Epilepsy, identity, and the experience of the body. *Epilepsy and Behavior* 2018; 89: 42–7.

8 Dako-Gyeke M, Donkor MD. Experiences and perspectives of stigmatization and discrimination against people with epilepsy in Accra, Ghana. *Epilepsy and Behavior* 2018. DOI:10.1016/j.yebeh.2018.07.025.

9 Scott AJ, Sharpe L, Thayer Z, *et al.* A qualitative examination and theoretical model of anxiety in adults with epilepsy. *Epilepsy and Behavior* 2018. DOI:10.1016/j.yebeh.2018.05.023.

10 Catalao R, Eshetu T, Tsigebrhan R, Medhin G, Fekadu A, Hanlon C. Implementing integrated services for people with epilepsy in primary care in Ethiopia: A qualitative study. *BMC Health Serv Res* 2018. DOI:10.1186/s12913-018-3190-y.

11 Egerod I, Wulff K, Petersen MC. Experiences and informational needs on sexual health in people with epilepsy or multiple sclerosis: A focus group investigation. *J Clin Nurs* 2018; 27: 2868–76.

12 Lukmanji S, Sauro KM, Josephson CB, Altura KC, Wiebe S, Jetté N. A longitudinal cohort study on the impact of the clobazam shortage on patients with epilepsy. *Epilepsia* 2018. DOI:10.1111/epi.13974.

13 Michaelis R, Niedermann C, Berger B. How Can We Enhance the Sense of Self-Efficacy in Epilepsy Individual Responses from 2 Qualitative Case Reports. *Complement Med Res* 2017; 24: 215–24.

14 Mugumbate J, Gray M. Individual resilience as a strategy to counter employment barriers for people with epilepsy in Zimbabwe. *Epilepsy and Behavior* 2017. DOI:10.1016/j.yebeh.2017.06.018.

15 Pembroke S, Higgins A, Pender N, Elliott N. Becoming comfortable with “my” epilepsy: Strategies that patients use in the journey from diagnosis to acceptance and disclosure. *Epilepsy and Behavior* 2017. DOI:10.1016/j.yebeh.2017.02.001.

16 Yennadiou H, Wolverson E. The experience of epilepsy in later life: A qualitative exploration of illness representations. *Epilepsy and Behavior* 2017. DOI:10.1016/j.yebeh.2017.01.033.

17 Crooks RE, Bell M, Patten SB, *et al.* Mind the gap: Exploring information gaps for the development of an online resource hub for epilepsy and depression. *Epilepsy and Behavior* 2017; 70: 18–23.

18 Hopker CDC, Berberian AP, Massi G, Willig MH, Tonocchi R. The individual with epilepsy: perceptions about the disease and implications on quality of life. *Codas* 2017. DOI:10.1590/2317-1782/20172015236.

19 Snape DA, Morgan M, Ridsdale L, Goodacre S, Marson AG, Noble AJ. Developing and assessing the acceptability of an epilepsy first aid training intervention for patients who visit UK emergency departments: A multi-method study of patients and professionals. *Epilepsy Behav* 2017; 68: 177–85.

20 Ridsdale L, Philpott SJ, Krooupa AM, Morgan M. People with epilepsy obtain added value from education in groups: results of a qualitative study. *Eur J Neurol* 2017; 24: 609–16.

21 Noble AJ, Robinson A, Snape D, Marson AG. ‘Epileptic’, ‘epileptic person’ or ‘person with epilepsy’? Bringing quantitative and qualitative evidence on the views of UK patients and carers to the terminology debate. *Epilepsy & Behavior* 2017; 67: 20–7.

22 RamachandranNair R, Jack SM. SUDEP: What do adult patients want to know? *Epilepsy Behav* 2016; 64: 195–9.

23 Mengoni SE, Gates B, Parkes G, *et al.* “Sometimes, it just stops me from doing anything”: A qualitative exploration of epilepsy management in people with intellectual disabilities and their carers. *Epilepsy & Behavior* 2016; 64: 133.

24 Collard SS, Marlow C. The psychosocial impact of exercising with epilepsy: A narrative analysis. *Epilepsy Behav* 2016; 61: 199–205.

25 Keikelame MJ, Swartz L. “The others look at you as if you are a grave”: a qualitative study of subjective experiences of patients with epilepsy regarding their treatment and care in Cape Town, South Africa. *BMC Int Health Hum Rights* 2016; 16: 1–9.

26 Ring A, Jacoby A, Baker GA, Marson A, Whitehead MM. Does the concept of resilience contribute to understanding good quality of life in the context of epilepsy? *Epilepsy Behav* 2016; 56: 153–64.

27 Johannessen Landmark C, Fløgstad I, Syvertsen M, *et al.* Treatment and challenges with antiepileptic drugs in patients with juvenile myoclonic epilepsy. *Epilepsy & Behavior* 2019; 98: 110–6.

28 Shamsalinia A, Masoudi R, Rad RE, Ghaffari F. Development and psychometric evaluation of the Perceived Social Stigma Questionnaire (PSSQ-for adults with epilepsy): A mixed method study. *Epilepsy Behav* 2019; 96: 141–9.

29 Molavi P, Sadeghie-Ahary S, Fattahzadeh-Ardalani G, Almasi S, Karimollahi M. The experiences of Iranian patients with epilepsy from their disease: A content analysis. *Epilepsy & Behavior* 2019; 96: 109–13.

30 Ring A, Jacoby A, Baker G, *et al.* What really matters? A mixed methods study of treatment preferences and priorities among people with epilepsy in the UK. *Epilepsy & Behavior* 2019; 95: 181–91.

31 Millogo A, Ngowi AH, Carabin H, Ganaba R, Da A, Preux PM. Knowledge, attitudes, and practices related to epilepsy in rural Burkina Faso. *Epilepsy Behav* 2019; 95: 70.

32 McDonald A, Goodwin J, Roberts S, *et al.* ‘We’ve made the best of it. But we do not have a normal life’: families’ experiences of tuberous sclerosis complex and seizure management. *J Intellect Disabil Res* 2019; 63: 947–56.

33 McKinlay AR, Ridsdale LL. Views of People With Epilepsy About Web-Based Self-Presentation: A Qualitative Study. *Interact J Med Res* 2018; 7: e10349.

34 Mejdahl CT, Schougaard LMV, Hjollund NH, Riiskjær E, Thorne S, Lomborg K. PRO-based follow-up as a means of self-management support – an interpretive description of the patient perspective. *J Patient Rep Outcomes* 2018; 2. DOI:10.1186/S41687-018-0067-0.

35 Hosseini N, Sharif F, Ahmadi F, Zare M. Determining the disease management process for epileptic patients: A qualitative study. *Iran J Nurs Midwifery Res* 2016; 21: 54.

36 Martin CM, Peterson C, Robinson R, Sturmberg JP. Care for chronic illness in Australian general practice - focus groups of chronic disease self-help groups over 10 years: implications for chronic care systems reforms. *Asia Pac Fam Med* 2009; 8. DOI:10.1186/1447-056X-8-1.

37 Laybourne AH, Morgan M, Watkins SH, Lawton R, Ridsdale L, Goldstein LH. Self-management for people with poorly controlled epilepsy: Participants’ views of the UK Self-Management in epILEpsy (SMILE) program. *Epilepsy & Behavior* 2015; 52: 159–64.

38 Bennett L, Bergin M, Gooney M, Doherty CP, Synnott C, Wells JSG. Epilepsy services in Ireland: ‘A survey of people with epilepsy in relation to satisfaction, preferences and information provision’. *Epilepsy Res* 2015; 113: 11–8.

39 Vears DF, Dunn KL, Wake SA, Scheffer IE. ‘It’s good to know’: experiences of gene identification and result disclosure in familial epilepsies. *Epilepsy Res* 2015; 112: 64–71.

40 Tonberg A, Harden J, McLellan A, Chin RFM, Duncan S. A qualitative study of the reactions of young adults with epilepsy to SUDEP disclosure, perceptions of risks, views on the timing of disclosure, and behavioural change. *Epilepsy and Behavior* 2015; 42: 98–106.

41 Sonecha S, Noble AJ, Morgan M, Ridsdale L. Perceptions and experiences of epilepsy among patients from black ethnic groups in South London. *Prim Health Care Res Dev* 2015; 16: 450–60.

42 Harden J, Tonberg A, Chin RF, McLellan A, Duncan S. ‘If you’re gonna die, you’re gonna die’: Young adults’ perceptions of sudden unexpected death in epilepsy. *Chronic Illn* 2015; 11: 230–41.

43 Walker ER, Barmon C, McGee RE, *et al.* Perspectives of Adults With Epilepsy and Their Support Persons on Self-Management Support. *http://dx.doi.org/101177/1049732314548880* 2014; 24: 1553–66.

44 Jacoby A, Ring A, Whitehead M, Marson A, Baker GA. Exploring loss and replacement of loss for understanding the impacts of epilepsy onset: a qualitative investigation. *Epilepsy Behav* 2014; 33: 59–68.

45 Espínola-Nadurille M, Crail-Melendez D, Sánchez-Guzmán MA. Stigma experience of people with epilepsy in Mexico and views of health care providers. *Epilepsy and Behavior* 2014; 32: 162–9.

46 Miller WR, Bakas T, Buelow JM. Problems, needs, and useful strategies in older adults self-managing epilepsy: Implications for patient education and future intervention programs. *Epilepsy and Behavior* 2014; 31: 25–30.

47 Thomas RH, Mullins JM, Hammond CL, Smith PEM, Kerr MP. The importance of the experiences of initial diagnosis and treatment failure when switching antiepileptic drugs. *Epilepsy & Behavior* 2013; 29: 492–6.

48 Hosseini N, Sharif F, Ahmadi F, Zare M. Patients’ perception of epilepsy and threat to self-identity: A qualitative approach. *Epilepsy & Behavior* 2013; 29: 228–33.

49 Wedlund EW, Nilsson L, Tomson T, Erdner A. What is important in rehabilitation for persons with epilepsy? Experiences from focus group interviews with patients and staff. *Epilepsy Behav* 2013; 28: 347–53.

50 Noble AJ, Morgan M, Virdi C, Ridsdale L. A nurse-led self-management intervention for people who attend emergency departments with epilepsy: the patients’ view. *J Neurol* 2013; 260: 1022–30.

51 Ridsdale L, Virdi C, Noble A, Morgan M. Explanations given by people with epilepsy for using emergency medical services: A qualitative study. *Epilepsy & Behavior* 2012; 25: 529–33.

52 Ryan S, Räisänen U. ‘The brain is such a delicate thing’: an exploration of fear and seizures among young people with epilepsy. *Chronic Illn* 2012; 8: 214–24.

53 Gauffin H, Flensner G, Landtblom AM. Living with epilepsy accompanied by cognitive difficulties: young adults’ experiences. *Epilepsy Behav* 2011; 22: 750–8.

54 Räty LK, Wilde-Larsson BM. Patients’ perceptions of living with epilepsy: a phenomenographic study. *J Clin Nurs* 2011; 20: 1993–2002.

55 Mushi D, Hunter E, Mtuya C, Mshana G, Behavior EA-E&, 2011 undefined. Social–cultural aspects of epilepsy in Kilimanjaro Region, Tanzania: knowledge and experience among patients and carers. *Elsevier* https://www.sciencedirect.com/science/article/pii/S1525505010007353 (accessed Nov 21, 2024).

56 Varley J, Delanty N, Normand C, Fitzsimons M. The health care journeys experienced by people with epilepsy in Ireland: what are the implications for future service reform and development? *Epilepsy Behav* 2011; 20: 299–307.

57 Aydemir N, Trung DV, Snape D, Baker GA, Jacoby A. Multiple impacts of epilepsy and contributing factors: Findings from an ethnographic study in Vietnam. *Epilepsy & Behavior* 2009; 16: 512–20.

58 McCorry D, Marson T, Jacoby A. Understanding routine antiepileptic drug decisions: A qualitative analysis of patients’ accounts of hospital consultations. *Epilepsy & Behavior* 2009; 14: 210–4.

59 Snape D, Wang W, Wu J, Jacoby A, Baker GA. Knowledge gaps and uncertainties about epilepsy: findings from an ethnographic study in China. *Epilepsy Behav* 2009; 14: 172–8.

60 Unger WR, Buelow JM. Hybrid concept analysis of self-management in adults newly diagnosed with epilepsy. *Epilepsy Behav* 2009; 14: 89–95.

61 Birbeck GL, Chomba E, Atadzhanov M, Mbewe E, Haworth A. Women’s Experiences Living with Epilepsy in Zambia. *Am J Trop Med Hyg* 2008; 79: 168.

62 Rhodes PJ, Small N, Ismail H, Wright JP. The use of biomedicine, complementary and alternative medicine, and ethnomedicine for the treatment of epilepsy among people of South Asian origin in the UK. *BMC Complement Altern Med* 2008; 8: 7.

63 Kilinç S, Campbell C. The experience of discontinuing antiepileptic drug treatment: an exploratory investigation. *Seizure* 2008; 17: 505–13.

64 Rhodes PJ, Small NA, Ismail H, Wright JP. ‘What really annoys me is people take it like it’s a disability’, epilepsy, disability and identity among people of Pakistani origin living in the UK. *Ethn Health* 2008; 13: 1–21.

65 Jacoby A, Wang W, Vu TD, *et al.* Meanings of epilepsy in its sociocultural context and implications for stigma: findings from ethnographic studies in local communities in China and Vietnam. *Epilepsy Behav* 2008; 12: 286–97.

66 Admi H, Shaham B. Living with epilepsy: ordinary people coping with extraordinary situations. *Qual Health Res* 2007; 17: 1178–87.

67 Paschal AM, Ablah E, Wetta-Hall R, Molgaard CA, Liow K. Stigma and safe havens: a medical sociological perspective on African-American female epilepsy patients. *Epilepsy Behav* 2005; 7: 106–15.

68 Prinjha S, Chapple A, Herxheimer A, McPherson A. Many people with epilepsy want to know more: a qualitative study. *Fam Pract* 2005; 22: 435–41.

69 Ismail H, Wright J, Rhodes P, Small N. Religious beliefs about causes and treatment of epilepsy. *British Journal of General Practice* 2005; 55.

70 Elwyn G, Todd S, Hibbs R, *et al.* A ‘real puzzle’: The views of patients with epilepsy about the organisation of care. *BMC Fam Pract* 2003; 4: 1–6.

71 Swarztrauber K, Dewar S, Behavior JEJ-E&, 2003 undefined. Patient attitudes about treatments for intractable epilepsy. *Elsevier* https://www.sciencedirect.com/science/article/pii/S152550500200687X (accessed Nov 21, 2024).

72 Ridsdale L, Kwan I, Morgan M. How can a nurse intervention help people with newly diagnosed epilepsy? A qualitative study (of patients’ views). *Seizure* 2002; 11: 1–5.

73 Wallace HK, Solomon JK. Quality of epilepsy treatment and services: the views of women with epilepsy. *Seizure* 1999; 8: 81–7.

74 Wilde M, Haslam C. Living with epilepsy: a qualitative study investigating the experiences of young people attending outpatients clinics in Leicester. *Seizure* 1996; 5: 63–72.
